# Endothelial progenitor cells stimulate neonatal lung angiogenesis through FOXF1-mediated activation of BMP9/ACVRL1 signaling

**DOI:** 10.1038/s41467-022-29746-y

**Published:** 2022-04-19

**Authors:** Guolun Wang, Bingqiang Wen, Zicheng Deng, Yufang Zhang, Olena A. Kolesnichenko, Vladimir Ustiyan, Arun Pradhan, Tanya V. Kalin, Vladimir V. Kalinichenko

**Affiliations:** 1 Center for Lung Regenerative Medicine, Perinatal Institute, Cincinnati Children’s Hospital Medical Center, Cincinnati, OH USA; 2 The Materials Science and Engineering Program, College of Engineering and Applied Science, University of Cincinnati, Cincinnati, OH USA; 3 Division of Pulmonary Biology, Cincinnati Children’s Hospital Medical Center, Cincinnati, OH USA; 4 Department of Pediatrics, University of Cincinnati, Cincinnati Children’s Hospital Medical Center, Cincinnati, OH USA; 5 Division of Developmental Biology, Cincinnati Children’s Hospital Medical Center, Cincinnati, OH USA

**Keywords:** Disease model, Respiratory tract diseases, Angiogenesis

## Abstract

Pulmonary endothelial progenitor cells (EPCs) are critical for neonatal lung angiogenesis and represent a subset of general capillary cells (gCAPs). Molecular mechanisms through which EPCs stimulate lung angiogenesis are unknown. Herein, we used single-cell RNA sequencing to identify the BMP9/ACVRL1/SMAD1 pathway signature in pulmonary EPCs. BMP9 receptor, ACVRL1, and its downstream target genes were inhibited in EPCs from *Foxf1*^*WT/S52F*^ mutant mice, a model of alveolar capillary dysplasia with misalignment of pulmonary veins (ACDMPV). Expression of *ACVRL1* and its targets were reduced in lungs of ACDMPV subjects. Inhibition of FOXF1 transcription factor reduced BMP9/ACVRL1 signaling and decreased angiogenesis in vitro. FOXF1 synergized with ETS transcription factor FLI1 to activate *ACVRL1* promoter. Nanoparticle-mediated silencing of ACVRL1 in newborn mice decreased neonatal lung angiogenesis and alveolarization. Treatment with BMP9 restored lung angiogenesis and alveolarization in ACVRL1-deficient and *Foxf1*^*WT/S52F*^ mice. Altogether, EPCs promote neonatal lung angiogenesis and alveolarization through FOXF1-mediated activation of BMP9/ACVRL1 signaling.

## Abstract (web summary)

The molecular mechanisms through which pulmonary endothelial progenitor cells stimulate lung angiogenesis are not clear. Here, authors show that these cells stimulate the growth of alveolar capillaries and alveoli of newborn mice through FOXF1 and FLI1 nuclear protein-activation of the BMP9/ACVRL1/SMAD1 signaling pathway.

## Introduction

Lung morphogenesis is a highly conserved developmental process, which requires specific signaling events and cell-to-cell interactions among a diversity of cells derived from endodermal, ectodermal, and mesodermal progenitors^1,2^. Formation of alveolus, a gas-exchange unit mediating transport of gases between inhaled air and the blood, occurs prior to and after birth, when respiratory epithelial progenitors differentiate into alveolar type I (AT1) and AT2 cells which are located in close proximity to capillary endothelial cells^1,3^. Defects in formation of alveoli and pulmonary capillaries are associated with severe pediatric lung diseases, such as Bronchopulmonary Dysplasia (BPD) and Alveolar Capillary Dysplasia with Misalignment of Pulmonary Veins (ACDMPV)^4–7^. Recent single cell RNA sequencing of human and mouse neonatal lungs identified two types of alveolar endothelial cells: general capillary cells (gCAPs) and alveolar capillary cells (aCAPs or aerocytes)^8^. aCAPs express APLN and CAR4, form a majority of alveolar gas-exchange surface area, and require signaling through Vascular Endothelial Growth Factor (VEGF) produced by AT1 cells^8–10^. gCAPs express APLNR and consist of several cell types, including mature gCAPs, cells of the embryonic vascular plexus and other endothelial cells with progenitor properties that are critical for pulmonary vascular development and lung repair after injury^8,11^. Endothelial progenitor cells (EPCs) expressing FOXF1 transcription factor and cKIT receptor (FOXF1^+^ EPCs) exhibit high colony-forming capacity and are capable of engraftment into the neonatal lung tissue of ACDMPV mice to form alveolar capillaries^11,12^. While EPCs hold promise for cell therapies in ACDMPV and other pediatric pulmonary disorders^13^, molecular mechanisms through which EPCs stimulate neonatal lung angiogenesis are unknown.

FOXF1 is a member of Forkhead Box (FOX) family of transcription factors, which is required for formation of pulmonary capillaries in mice and humans^1,3^. Heterozygous loss-of-function mutations in *FOXF1* are linked to ACDMPV, a severe congenital disorder which is characterized by diminished lung angiogenesis leading to scarcity of the alveolar capillary network^4^. ACDMPV causes respiratory distress and mortality within the first month of birth in spite of intensive care^4,5,14^. Global deletion of the *Foxf1* gene in mice is embryonic lethal^15^, whereas haploinsufficiency of *Foxf1* (*Foxf1*^*+/-*^) or heterozygous *S52F Foxf1 knock-in* mutation (*Foxf1*^*WT/S52F*^) disrupts development of alveolar capillaries, recapitulating human ACDMPV^14,16^. FOXF1 stimulates lung angiogenesis and vascular repair after injury by accelerating endothelial proliferation while maintaining endothelial cell junctions to prevent hemorrhage^17,18^. FOXF1 regulates expression of genes critical for VEGF, PDGF, NOTCH and TIE2 signaling pathways during lung development and regeneration^19–22^. While genetic and histological evidence strongly suggest that endothelial cells play a key role in ACDMPV pathogenesis^4,14^, endothelial cell diversity and key signaling pathways in ACDMPV remain unclear.

The TGFβ/BMP signaling pathway has been implicated in vascular development. Abnormalities in TGFβ/BMP signaling in endothelium lead to developmental defects in the vasculature^23^. Hereditary hemorrhagic telangiectasia (HHT) is an autosomal dominant disorder which is associated with severe abnormalities in the microvascular network and linked to mutations in *ACVRL1* and *ENG* genes^24–26^. ACVRL1 forms a receptor complex with BMPR2 and ENG to mediate TGFβ/BMP signaling in endothelial cells through phosphorylation of SMAD1/5/8. BMP9, a high-affinity ligand for the ACVRL1/BMPR2/ENG receptor^27^, is produced by hepatocytes and delivered by the blood to other organs^28^. BMP9 promotes proliferation of endothelial cells in vitro and in vivo^27^, and ameliorates pulmonary arterial hypertension (PAH) through activation of endothelial BMPR2 signaling^29^. While circulating BMP9 prevents alveolar simplification after neonatal hyperoxia^30^ and protects pulmonary endothelium after LPS-mediated lung injury^31^, it is unclear which endothelial cell lineages require BMP9 signaling to promote lung repair and regeneration.

In the present study, we used single cell RNA sequencing to identify a genomic signature of the BMP9/ACVRL1/SMAD1signaling pathway in pulmonary FOXF1^+^ EPCs. We also used a mouse model of ACDMPV to demonstrate that FOXF1^+^ EPCs stimulate neonatal lung angiogenesis through FOXF1-mediated activation of BMP9/ACVRL1 signaling.

## Results

### The *S52F Foxf1* mutation alters cellular composition of the mouse lung prior to birth

**Fig. 1:**
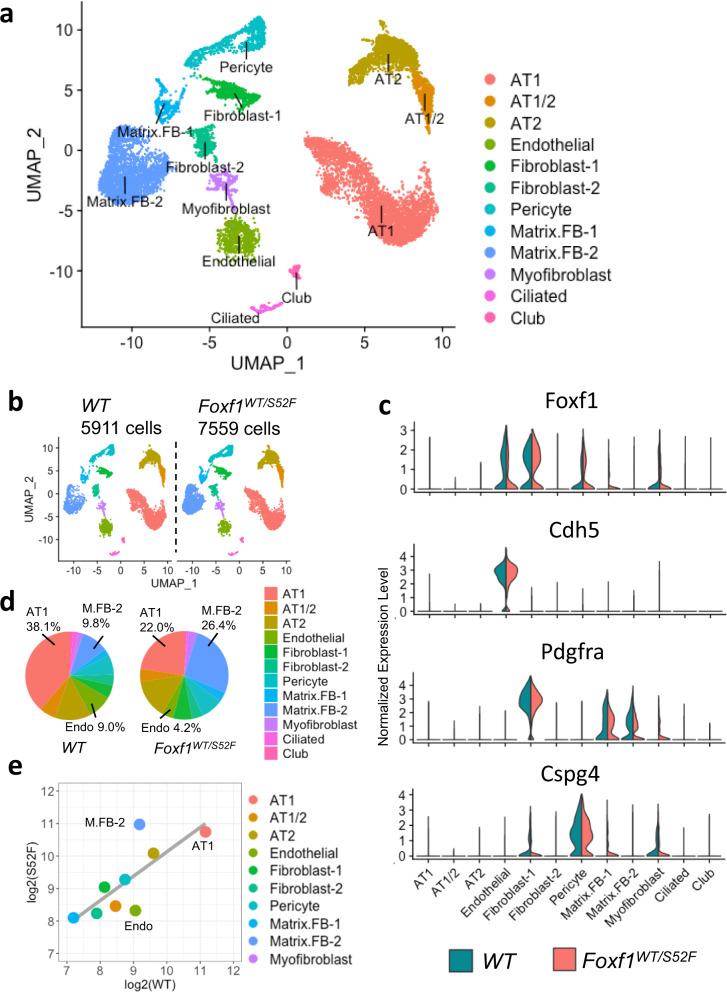
Single cell RNAseq analysis identifies differences in cellular composition of the *Foxf1*^*WT/S52F*^ lung. **a** The integrated projection of CD45-depleted pulmonary cells from *WT* and *Foxf1*^*WT/S52F*^ lungs. Lungs were harvested from E18.5 embryos and enzymatically digested to obtain single cell suspensions. CD45-positive cells were depleted using immunomagnetic beads. Three embryos from each genotype were pooled together prior to the scRNAseq analysis. 12 cell clusters were identified using the Uniform Manifold Approximation and Projection (UMAP) clustering. **b** A K-nearest neighbor (KNN) graph-based clustering approach was used to identify similar cell clusters in *WT* (*n* = 5911 cells) and *Foxf1*^*WT/S52F*^ lungs (*n* = 7559 cells). **c** Violin plots show the presence of *Foxf1* mRNA in endothelial cells, fibroblast-1, pericytes and myofibroblasts based on expression of selective cell markers *Cdh5, Pdgfra* and *Cspg4*. **d** Proportions of cells in each of the 12 clusters are compared in *WT* and *Foxf1*^*WT/S52F*^ lungs. Percentages of endothelial and AT1 cells are decreased in *Foxf1*^*WT/S52F*^ lungs. The percentage of matrix FB2 is increased in *Foxf1*^*WT/S52F*^ lungs compared to lungs of WT littermates. **e** Linear regression analysis shows changes in distribution of endothelial, AT1 and matrix FB2 clusters between *WT* and *Foxf1*^*WT/S52F*^ lungs.

We previously generated *Foxf1*^*WT/S52F*^ mice in which the evolutionary conserved *serine 52* in the FOXF1 DNA binding domain was replaced with *phenylalanine* in one of the *Foxf1* alleles, recapitulating the *S52F FOXF1* mutation found in ACDMPV patients^14^. Since *Foxf1*^*WT/S52F*^ mice developed alveolar capillary dysplasia phenotypically similar to human ACDMPV^14^, we used single cell RNA sequencing (scRNAseq) of lung tissue to identify molecular mechanisms through which the *S52F FOXF1* mutation causes ACDMPV. Single cell transcriptomes from lungs of *Foxf1*^*WT/S52F*^ and control *WT* E18.5 embryos were compared. Since FOXF1 is not expressed in hematopoietic cell lineages^1,19^, CD45^+^ cells were depleted from lung cell suspensions using immunomagnetic beads. Efficient depletion of CD45^+^ cells was confirmed by FACS analysis (Supplementary Fig. 1a, b). Cells from three embryos were combined in each group prior to single cell RNA sequencing. After confirming RNA quality and filtering outlier cells and cell doublets at both cell and feature levels (Supplementary Fig. 1c), 7559 cells from *Foxf1*^*WT/S52F*^ and 5911 cells from *WT* lungs were subjected to bioinformatic analysis using the anchor-based integrative pipeline from Seurat 3.0^32^. Twelve major cell clusters were identified by unsupervised clustering (Fig. 1a, b). These include epithelial AT1, AT2, AT1/AT2, ciliated and club cells, endothelial cells and six mesenchymal cell clusters: fibroblasts 1 (FB-1), FB-2, pericytes, matrix FB-1, matrix FB-2 and myofibroblasts. The integrated cell clustering of E18.5 mouse lungs was performed using cell-specific gene signatures of each cluster (Supplementary Figs. 1d, e and 2a–d) that were identified in published studies^1,33,34^. *Foxf1* mRNA was detected in endothelial cells, FB-1, pericytes and myofibroblasts, but not in *Nkx2-1*-expressing epithelial cells (Fig. 1c and Supplementary Fig. 2e).

While all twelve major cell clusters were present in *Foxf1*^*WT/S52F*^ lungs, cellular composition of the mutant lung was changed compared to *WT* controls (Fig. 1d). Percentages of endothelial and AT1 cells were decreased, whereas the percentage of matrix FB-2 was increased in *Foxf1*^*WT/S52F*^ lungs compared to lungs of *WT* littermates (Fig. 1d). These findings were supported by the linear regression analysis of cell distribution across the clusters (Fig. 1e), and by immunostaining of lung tissue sections using antibodies specific to ERG, HOPX and FN1 (Supplementary Fig. 3). Thus, the *S52F Foxf1* mutation alters cellular composition in the mouse lung prior to birth. Since neither AT1 nor matrix FB-2 cells express *Foxf1* (Fig. 1c) and gene expression signatures in these cell types were similar between *Foxf1*^*WT/S52F*^ and *WT* lungs (Supplementary Fig. 4a–d), we focused our next studies on pulmonary endothelial cells.

### The *S52F Foxf1* mutation disrupts ACVRL1 signaling in gCAPs

**Fig. 2:**
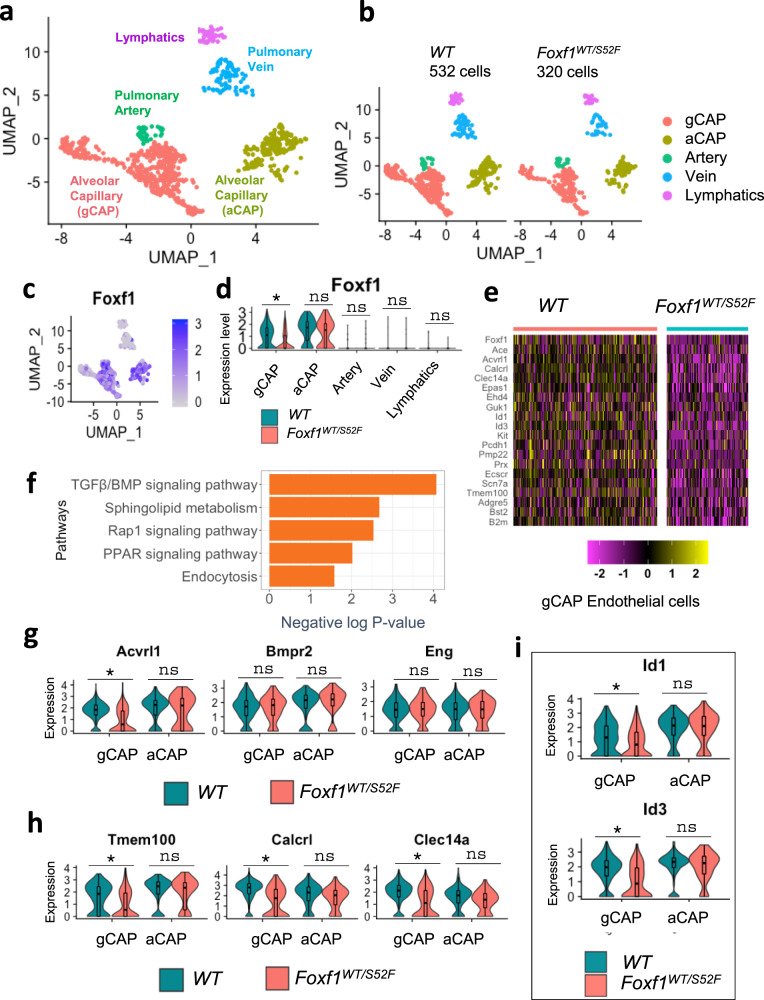
Single cell RNAseq analysis reveals decreased ACVRL1 expression in gCAPs of *Foxf1*^*WT/S52F*^ mice. **a** Dimensional reduction plot with UMAP shows five subclusters of pulmonary endothelial cells in E18.5 lungs. Lungs were harvested from E18.5 mouse embryos, enzymatically digested and then incubated with CD45^+^ immunobeads to deplete hematopoietic cells. Three embryos from each genotype were pooled together prior to the scRNAseq analysis. **b** Split dimensional reduction plots of pulmonary endothelial cells from *WT* (*n* = 532 cells) and *Foxf1*^*WT/S52F*^ embryos (*n* = 320 cells). **c**, **d** Violin and scatter plots show an enrichment of *Foxf1* mRNA in pulmonary capillary cells. *Foxf1* expression is selectively decreased in gCAPs but not aCAPs from *Foxf1*^*WT/S52F*^ embryos. *T*-test (two-tailed) analyses were performed, and pvalue for Foxf1 is 0.01257. *p < 0.05, *p* > 0.05 is ns. **e** Heatmap shows representative gCAP genes, expression of which is decreased in *Foxf1*^*WT/S52F*^ embryos compared to *WT* embryos. **f** Analysis of signaling pathways demonstrates that gCAPs of *Foxf1*^*WT/S52F*^ embryos exhibit decreased expression of genes associated with the TGFβ/BMP signaling pathway, sphingolipid metabolism, endocytosis, Rap1 and PPAR pathways. Fisher’s Exact (one-sided) test was used via DAVID bioinformatic resource to measure gene-enrichment *p*-values. **g** Violin plots show decreased expression of *Acvrl1* in gCAPs but not aCAPs. Expression of *Bmpr2* and *Eng* is unchanged *T*-test (two-tailed) analyses were performed, and p-value for Acvrl1 is 0.01993. *p* < 0.05 is *p > 0.05 is ns. **h**, **i** Violin plots show decreased expression of downstream target genes of the TGFβ/BMP signaling pathway in pulmonary gCAPs, including *Tmem100, Calcrl, Clec14a*, *Id1* and *Id3*. *T*-test (two-tailed) analyses were performed, and pvalue for Tmem100 is 0.01688, for Calcrl is 0.02132, for Clec14a is 0.0213, for Id1 is 0.03623, for Id3 is 0.037811. *p < 0.05, *p* > 0.05 is ns. The data for box plots in **d** and **g**–**i** were retrieved from single cell normalized counts (WT: *n* = 532; *Foxf1*^*WT/S52F*^: *n* = 320) Boxplots: center line, median; box boundary, first and third quartiles; whiskers denote 5th–95th percentile. Expression of these genes in pulmonary aCAPs was unchanged. ns is not significant.

Consistent with recently described gene signatures of pulmonary endothelial cell lineages^8,35^, 320 endothelial cells from *Foxf1*^*WT/S52F*^ and 532 endothelial cells from *WT* lungs were subdivided into 5 sub-clusters (Fig. 2a, and Supplementary Fig. 5a, b). These included arterial, venous, lymphatic, general capillary cells (gCAPs) and aerocytes (aCAPs). *Foxf1* mRNA was mainly expressed in gCAPs and aCAPs (Fig. 2c, d). *Foxf1*-expressing cells were also detected among arterial and venous clusters but were absent in the lymphatics (Fig. 2c).

*Foxf1* mRNA was selectively decreased in *Foxf1*^*WT/S52F*^ gCAPs but not in other endothelial cell types (Fig. 2d). Using the likelihood-ratio test, we generated a heatmap and identified gene expression differences between gCAPs from *Foxf1*^*WT/S52F*^ and *WT* lungs. Expression of 93 genes was decreased in *Foxf1*^*WT/S52F*^ gCAPs compared to controls (Fig. 2e and Supplementary Table 1), whereas expression of 49 genes was increased (Supplementary Table 2). Bioinformatic analysis identified several functional categories that were impaired in *Foxf1*^*WT/S52F*^ gCAPs (Fig. 2f). Expression of genes critical for the TGFβ/BMP signaling pathway was decreased in *Foxf1*^*WT/S52F*^ gCAPs compared to control (Fig. 2f and Supplementary Table 3). A systematic profiling of mRNAs encoding TGFβ/BMP receptors among pulmonary cell types showed that *Acvrl1* (also known as ALK1) and *Bmpr2* are the main TGFβ type I and type II receptors in endothelial cells (Supplementary Fig. 6a, b). Decreased expression of *Foxf1* in *Foxf1*^*WT/S52F*^ gCAPs (Fig. 2d) was associated with reduced *Acvrl1* mRNA (Fig. 2g). Neither *Foxf1* nor *Acvrl1* mRNAs were changed in aCAPs of *Foxf1*^*WT/S52F*^ mice (Fig. 2d–g). We also used scRNAseq to examine expression of downstream target genes of the ACVRL1 signaling pathway, such as *Tmem100, Calcrl, Clec14a, Id1 and Id3*^26,36,37^. mRNAs of these genes were enriched in pulmonary endothelial cells compared to other cell types (Supplementary Fig. 6a). Expression of *Tmem100, Calcrl, Clec14a, Id1 and Id3* was decreased in *Foxf1*^*WT/S52F*^ gCAPs compared to gCAPs from *WT* lungs (Fig. 2h, i), a finding consistent with impaired ACVRL1 signaling. Decreased expression of *Acvrl1* and *Tmem100* in *Foxf1*^*WT/S52F*^ gCAPs was also confirmed by RNAscope (Supplementary Fig. 7). In contrast, expression of ACVRL1 target genes was unchanged in aCAPs (Fig. 2h, i). The *Foxf1*^*WT/S52F*^ mutation did not alter *Bmpr2* and *Eng* mRNAs in either gCAPs or aCAPs (Fig. 2g).

### Single cell RNAseq and RNAscope identify heterogeneity of pulmonary gCAPs

**Fig. 3:**
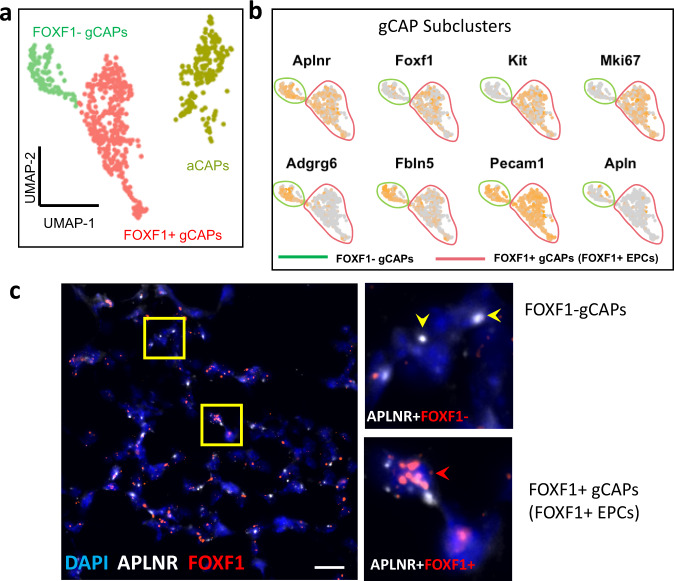
FOXF1 is expressed in a subset of gCAPs. **a**, **b** Dimensional reduction plot with UMAP was generated using scRNAseq dataset from *WT* E18.5 lungs and shows gCAP and aCAP clusters of microvascular endothelial cells. Three embryos were pooled together prior to the scRNAseq analysis. gCAPs consist of two distinct subclusters: FOXF1^+^ gCAPs and FOXF1^–^ gCAPs. **c** RNAscope of lung sections from *WT* E18.5 embryos shows APLNR-expressing gCAPs in the alveolar region. High magnification images of area in yellow box are shown on the right. Lung sections were counterstained with DAPI. APLNR co-localizes with FOXF1 in FOXF1^+^ gCAPs (red arrowheads). FOXF1 is not expressed in FOXF1^–^ gCAPs (yellow arrowheads). The RNAscope assays were performed twice with consistent results. Scale bars are 10 μm.

Based on scRNAseq analysis of murine *WT* E18.5 lungs, *Aplnr*-expressing gCAPs consisted of two distinct cell subsets: FOXF1^–^ gCAPs and FOXF1^+^ gCAPs (Fig. 3a, b). Both gCAP subsets resided in the alveolar region as demonstrated by RNAscope using riboprobes specific to *Aplnr* and *Foxf1* (Fig. 3c). FOXF1^+^ gCAPs expressed *Kit* (Fig. 3b) and were similar to recently described FOXF1^+^cKIT^+^ lung endothelial progenitor cells (FOXF1^+^ EPCs)^11^. The presence of *Mki67* in FOXF1^+^ EPCs (Fig. 3b and Supplementary Table 4) indicates that these cells are highly proliferative. In contrast, FOXF1^–^ gCAPs did not expressed *Mki67* but were positive for *Adgrg6* and *Fbln5* (Fig. 3b and Supplementary Table 5).

**Fig. 4:**
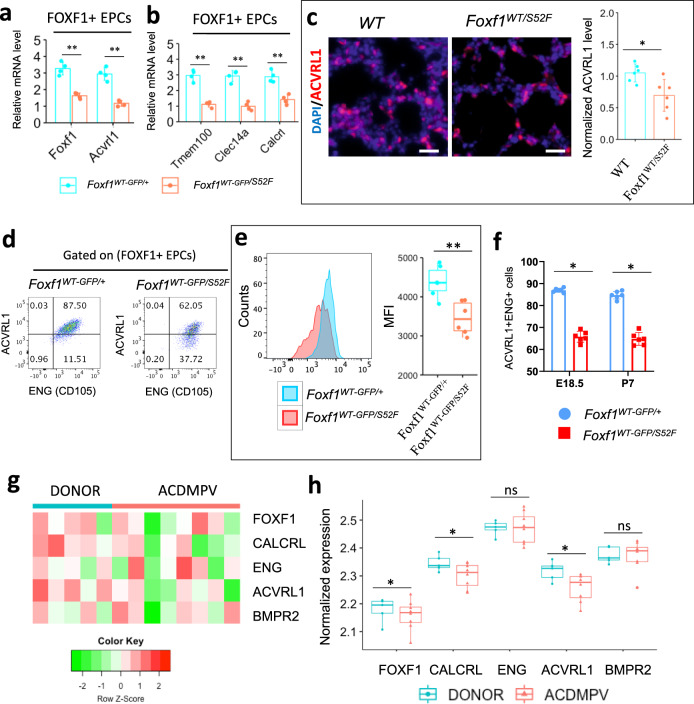
Expression of ACVRL1 is decreased in mouse and human ACDMPV lungs. **a** *Foxf1* and *Acvrl1* mRNAs are decreased in FACS-sorted gCAPs of *Foxf1*^*WT-GFP/S52F*^ embryos. FOXF1^+^ gCAPs (FOXF1^+^cKIT^+^CD31^+^CD45^–^) were purified from enzymatically digested E18.5 mouse lungs (*n* = 4 embryos in each group). FOXF1-positive gCAPs were identified using the *Foxf1-GFP* transgene, which contains *GFP* knocked into the endogenous *Foxf1* gene locus. *Foxf1*^*WT-GFP/+*^ embryos were used as controls. Data were shown as mean ± SD. Nonparametric Mann-Whitney U test were performed, and *p* value for Foxf1 is 0.00022, for Acvrl1 is 0.00015. **b** Expression of ACVRL1 downstream target genes is decreased in FACS-sorted gCAPs of *Foxf1*^*WT-GFP/S52F*^ embryos (*n* = 4 embryos in each group). Data were shown as mean ± SD, and nonparametric Mann-Whitney U test were performed were performed, and *p* value for Tmem100 is 0.00009, for Calcrl is 0.00098, for Clec14a is 0.00011. **c** Immunostaining of E18.5 lungs shows reduced expression of ACVRL1 protein in *Foxf1*^*WT/S52F*^ lungs compared to *WT* controls (*n* = 6). Scale bars are 10 μm. Data were presented as mean ± SD, and *T*-test (two-tailed) were performed, *p* value = 0.01293. **d** The percentage of ACVRL1^+^ENG^+^ cells among pulmonary FOXF1^+^ gCAPs is decreased in *Foxf1*^*WT-GFP/S52F*^ E18.5 embryos. **e** Measurements of mean fluorescence intensity (MFI) by FACS analysis show decreased ACVRL1 cell surface expression in FOXF1^+^ gCAPs of *Foxf1*^*WT-GFP/S52F*^ embryos compared to *Foxf1*^*WT-GFP/+*^ controls. (*n* = 6 mice in each group) Boxplots: center line, median; box boundary, first and third quartiles; whiskers denote 5th–95th percentile, *p* value = 0.00298 **f** Percentages of ACVRL1^+^ENG^+^ gCAPs are lower in *Foxf1*^*WT-GFP/S52F*^ mice at E18.5 and P7 (*n* = 6 mice in each group). Data were presented as mean ± SD. *T*-test (two-tailed) were performed, *p* value for E18.5 is 6.20943E-07, and *p* value for P7 is 3.38604E-06. Source data are provided as a Source Data file. **g**, **h**, Microarray analysis shows decreased expression of *FOXF1*, *ACVRL1* and *CALCRL* mRNAs in human ACDMPV lungs (*n* = 8) compared to donor lungs (*n* = 5). *BMPR2* and *ENG* mRNAs were unaltered. Boxplots: center line, median; box boundary, first and third quartiles; whiskers denote 5th–95th percentile, and student’s *T*-test (two-tailed) were performed. **p < 0.01, *p < 0.05 is, ns is not significant.

FOXF1 deficiency disrupts ACVRL1 signaling in mouse and human lungs. We used FACS analysis and fluorescent microscopy to confirm that ACVRL1 and ENG proteins are co-expressed in pulmonary endothelial cells (Supplementary Fig. 8a, b). Using cell sorting, FOXF1^+^ EPCs (FOXF1^+^cKIT^+^CD31^+^CD45^–^) were purified from lungs of *Foxf1*^*WT-GFP/S52F*^ E18.5 reporter embryos (Supplementary Fig. 9a), in which the first *Foxf1* allele contains the *S52F Foxf1* mutation and the second *Foxf1* allele produces two separate *WT-Foxf1* and *GFP* reporter transcripts^11^. Expression of *Foxf1, Acvrl1* and its downstream target genes was lower in FACS-sorted *Foxf1*^*WT-GFP/S52F*^ EPCs compared to EPCs from *Foxf1*^*WT-GFP/+*^ controls (Fig. 4a, b and Supplementary Fig. 9b). In contrast, expression of *Bmpr2*, *Eng* and other TGFβ/BMP receptors was unchanged (Supplementary Fig. 9b, c). ACVRL1 staining was decreased in *Foxf1*^*WT/S52F*^ lungs (Fig. 4c). Furthermore, *Foxf1*^*WT-GFP/S52F*^ EPCs exhibited decreased cell surface expression of ACVRL1 (Fig. 4d, e) and reduced numbers of EPCs co-expressing ACVRL1 and ENG (ACVRL1^+^ENG^+^FOXF1^+^cKIT^+^CD31^+^CD45^–^) (Fig. 4f). Finally, we used publicly available RNAseq datasets from lungs of patients with ACDMPV^38^ to examine expression of genes critical for the ACVRL1 signaling pathway. mRNAs of *ACVRL1* and its target gene *CALCRL* were decreased in ACDMPV lungs (*n* = 8) compared to donor lungs (*n* = 5) (Fig. 4g, h). Consistent with murine *Foxf1*^*WT/S52F*^ datasets, mRNAs of *BMPR2*, *ENG* and other TGFβ/BMP receptors were unchanged in human ACDMPV lungs (Fig. 4g, h and Supplementary Fig. 10a, b). Altogether, FOXF1 deficiency decreases ACVRL1 and expression of its downstream target genes in human and mouse lungs.

### FOXF1 transcriptionally stimulates *Acvrl1* through an evolutionarily conserved ACE80 promoter region

**Fig. 5:**
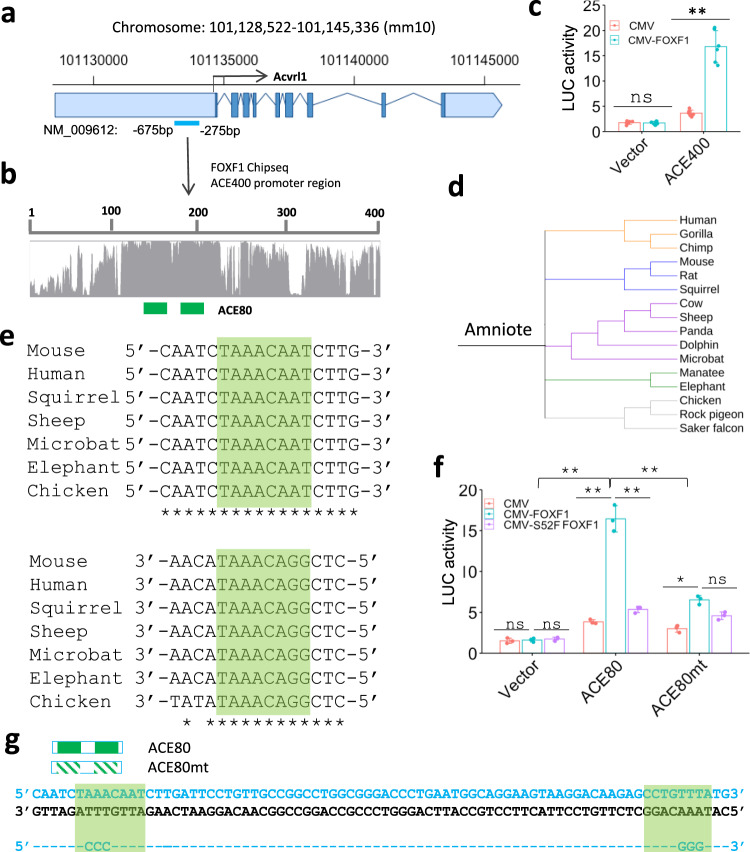
FOXF1 activates *Acvrl1* gene expression through the ACE80 promoter region. **a**, **b** Schematic diagram of the mouse *Acvrl1* gene locus which contains the evolutionarily conserved −675/−275 promoter region (ACE400). Light blue boxes show untranslated DNA regions. Dark blue boxes indicate exons in the *Acvrl1* gene. Two FOXF1-binding sites are indicated by green boxes. **c** Dual luciferase assay shows that *CMV-FOXF1* expression plasmid stimulates the ACE400 activity in co-transfection experiments in MFLM-91U cells (*n* = 6). Data were shown as mean ± SD. *T*-test (two-tailed) were performed, pvalue for ACE is 0.00000158. *p* > 0.05 is not significant. *CMV-empty* expression plasmid (pCMV) and an empty LUC plasmid (vector) were used as controls. **d** Schematic shows the evolutionary conservation of ACE80 promoter region in amniote vertebrates. Amniotes were classified using UCSC genome browser into five groups: primates (orange), *Afrotheria* mammals (green), *Laurasiatheria* mammals (purple), *Euarchontoglires* mammals (blue) and birds (grey). **e** Schematic shows sequences of two FOXF1-binding sites in ACE80 region in several species. **f** Dual luciferase assay shows that *CMV-FOXF1* expression vector stimulates the ACE80 activity in co-transfection experiments in MFLM-91U cells (*n* = 3). Data were shown as mean ± SD. The data were analyzed by one-way ANOVA followed by Tukey’s test (two-tailed). *CMV-empty* vector (CMV) and transcriptionally inactive S52F FOXF1 mutant plasmid (*CMV- S52F-FOXF1*) are used as controls. LUC activity is decreased after co-transfection of *CMV-FOXF1* plasmid with ACE80mt LUC reporter. **p < 0.01, *p < 0.05, ns is not significant. **g** Location of FOXF1-binding sites in ACE80 region is indicated by green boxes. FOXF1-binding sites in ACE80mt construct were inactivated by disrupting the FOX core motif.

Next, we determined whether *Acvrl1* is a direct transcriptional target of FOXF1. Based on publicly available FOXF1 ChIPseq datasets from murine E18.5 lungs^39^, FOXF1 directly bound to DNA in the *Acvrl1* promoter region (Supplementary Fig. 11a, b). Using a comparative genomic analysis of eutherian mammals, we identified an evolutionarily conserved 400 bp DNA region located in the *Acvrl1* gene (ACE400; −675/−275 bp) (Fig. 5a, b and Supplementary Fig. 11a, b). The −675/−275 bp *Acvrl1* promoter region was amplified from mouse genomic DNA and cloned into the luciferase (LUC) reporter plasmid. In co-transfection experiments with fetal lung endothelial MFLM-91U cells, *CMV-FOXF1* expression vector increased LUC activity driven by the ACE400 promoter region (Fig. 5c). Thus, FOXF1 directly activates the ACE400 *Acvrl1* promoter region in endothelial cells. Interestingly, the ACE400 region contains two FOXF1-binding sites within the 80 bp sub-fragment (ACE80; −538/−458 bp) (Supplementary Fig. 11c). The ACE80 DNA region of the *Acvrl1* promoter is evolutionarily conserved between mammals and birds (Fig. 5d, e and Supplementary Fig. 12). *CMV-FOXF1* expression vector increased LUC activity driven by the ACE80 promoter region, whereas CMV vector with *S52F FOXF1* mutant was transcriptionally inactive (Fig. 5f). Site-directed mutagenesis was used to inactivate both FOXF1-binding sites in the ACE80 region and generate the ACE80 mutant LUC construct (Fig. 4g). Inactivation of FOXF1-binding sites decreased the ability of *CMV-FOXF1* vector to stimulate transcriptional activity of the ACE80 promoter region (Fig. 5f). Taken together, FOXF1 transcriptionally activates the *Acvrl1* gene through FOXF1-binding sites located in the evolutionarily conserved –538/−458 bp *Acvrl1* promoter region.

### FOXF1 synergizes with ETS transcription factor FLI1 to stimulate *Acvrl1* promoter activity

**Fig. 6:**
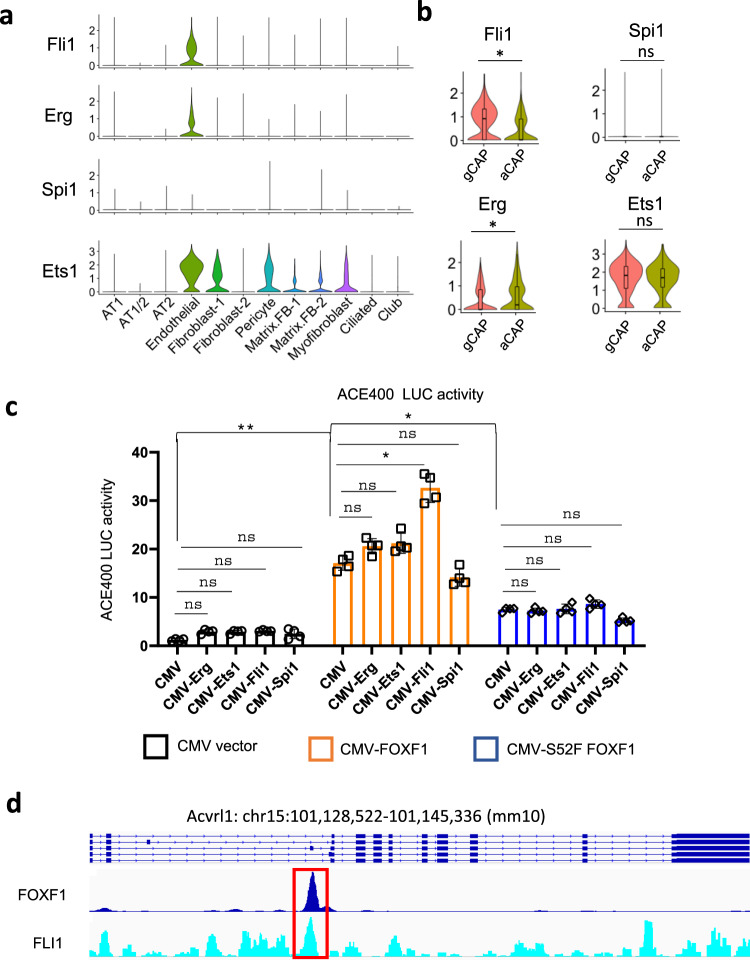
FOXF1 synergizes with FLI1 to stimulate *Acvrl1* promoter activity. **a** Violin plots were generated using a combined pool of cells from *WT* and *Foxf1*^*WT/S52F*^ E18.5 lungs and show endothelial-enriched expression of ETS transcription factors *Fli1*, *Erg* and *Ets1*. ETS transcription factor *Spi1* is not expressed in endothelial, epithelial, and mesenchymal cell lineages in the lung tissue. **b** Violin plots show that *Fli1* is enriched in gCAPs, whereas *Erg* is enriched in aCAPs. *Ets1* expression is similar in aCAPs and gCAPs. The data for box plots were from single cell normalized counts (WT: *n* = 532; *Foxf1*^*WT/S52F*^: *n* = 320). Boxplots: center line, median; box boundary, first and third quartiles; whiskers denote 5th–95th percentile. *T*-test (two-tailed) were performed, pvalue for Fli1 is 0.01677, and for Erg is 0.02791. **c** Dual luciferase (LUC) assay shows transcriptional synergy between CMV-FOXF1 and CMV-FLI1 expression vectors to activate the ACE400 LUC reporter. Co-transfection experiments were performed using fetal lung MFLM-91U cells (*n* = 4). Data were shown as mean ± SD, and one-way ANOVA followed by Tukey’s test (two-tailed) were performed. **p < 0.01, *p < 0.05, ns is not significant. **d** ChIPseq shows that FOXF1 and FLI1 proteins bind to the ACE400 region of *Acvrl1* gene (red box).

Since both aCAPs and gCAPs express FOXF1 but *Acvrl1* mRNA was only reduced in *Foxf1*^*WT/S52F*^ gCAPs (Fig. 2c–g), we tested the possibility that regulation of *Acvrl1* promoter requires other transcription factors in addition to FOXF1. Comparative genomic analysis identified two conserved ETS binding sites near FOXF1 sites in the −538/−458 bp *Acvrl1* promoter region (Supplementary Fig. 13a, c). Based on scRNAseq data from *WT* E18.5 lungs, endothelial cells expressed several ETS transcription factors, including *Fli1*, *Erg* and *Ets1* (Fig. 6a). *Fli1* mRNA was enriched in gCAPs compared to aCAPs (Fig. 6b). Both FOXF1^–^ and FOXF1^+^ subsets of gCAPs expressed high levels of *Fli1* (Supplementary Fig. 13d). *Erg* was enriched in aCAPs (Fig. 6b) and FOXF1^–^ gCAPs (Supplementary Fig. 13d), whereas *Ets1* expression was high in both aCAPs and gCAPs (Fig. 6b). Next, we performed co-transfection experiments, testing whether FOXF1 synergizes with FLI1, ERG and ETS1 to regulate *Acvrl1* promoter activity in fetal lung endothelial MFLM-91U cells. ETS transcription factor SPI1, which is not expressed in lung endothelial cells (Fig. 6a, b and Supplementary Fig. 13d) was also used in these experiments. FOXF1 synergized with FLI1 but not with other ETS transcription factors to stimulate *Acvrl1* promoter activity in vitro (Fig. 6c). Endogenous FLI1 and FOXF1 proteins directly bound to DNA in the −538/−458 bp *Acvrl1* promoter region as demonstrated by ChIPseq (Fig. 6d). The *S52F FOXF1* mutant, which is deficient in DNA binding^14^, did not synergize with FLI1 to activate the *Acvrl1* promoter (Fig. 6c). Thus, FOXF1 synergizes with ETS transcription factor FLI1 to stimulate *Acvrl1* promoter activity in FOXF1^+^ EPCs. Interestingly, FOXF1 and ETS binding sites were also found in the −1170/−1118 bp *Foxf1* promoter region (FARE350), which is conserved in mammals (Supplementary Fig. 14a–c). The −1170/−1118 bp *Foxf1* promoter region was amplified from mouse genomic DNA and cloned into the LUC reporter plasmid. Similar to the *Acvrl1* promoter, FOXF1 synergized with FLI1 to stimulate its own promoter activity, whereas the *S52F FOXF1* mutant had no effect (Supplementary Fig. 15).

### FOXF1 stimulates BMP9/ACVRL1 signaling in vitro

**Fig. 7:**
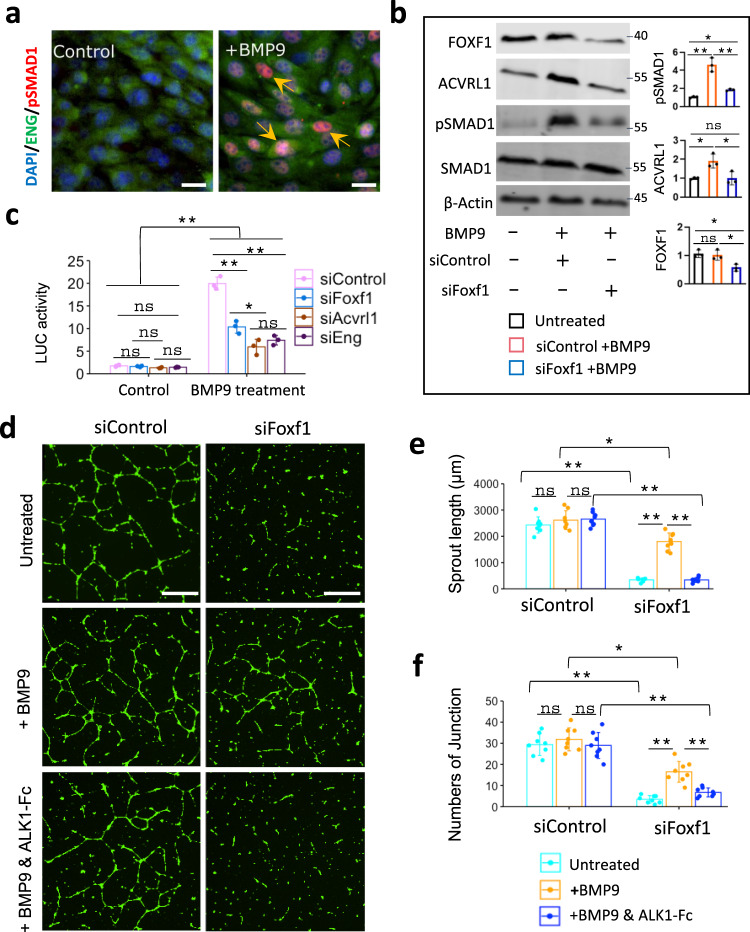
BMP9 stimulates angiogenesis in FOXF1-deficient endothelial cells in vitro. **a** Immunostaining shows an increase in phosphorylated SMAD1 (pSMAD1) after treatment of endothelial MFLM-91U cells with BMP9. Nuclear pSMAD1 staining (red) is indicated by arrows. Endothelial cells are counterstained with DAPI (blue) and ENG (green). The experiment was repeated 3 times with similar results. Scale bars are 10 μm. **b** Western blot shows that BMP9 treatment increases pSMAD1 and ACVRL1 protein levels but does not affect FOXF1 or total SMAD1 in MFLM-91U cells (*n* = 3). Inhibition of FOXF1 by siRNA decreases pSMAD1 and ACVRL1 proteins in BMP9-treated cells. **c** Dual luciferase assay shows that BMP9 increases activity of the BRE-LUC reporter plasmid in MFLM-91U cells (*n* = 3). siRNA-mediated inhibition of FOXF1, ACVRL1 or ENG decreases BRE-LUC reporter activity in BMP9-treated cells. **b**, **c** Data were shown as mean ± SD, and one-way ANOVA followed by Tukey’s test (two-tailed) were performed. **p < 0.01, *p < 0.05 ns is not significant. **d**–**f** In vitro angiogenesis assay shows that inhibition of FOXF1 by siRNA decreases the formation of endothelial sprouts in MFLM-91U cells. BMP9 treatment partially restores the in vitro angiogenesis in FOXF1-deficient endothelial cells. Treatment of cells with ALK1-Fc chimeric protein inhibits the effect of BMP9. Scale bars are 20 μm. The complexity of the vascular network in Matrigel is quantitated by measurements of sprout length **e** and counts of the sprout junctions **f** (*n* = 8 per each group). **e**, **f** Data were shown as mean ± SD, and one-way ANOVA (two-tailed) followed by Tukey’s multiple comparison test was performed. **p < 0.01, *p < 0.05, ns is not significant.

BMP9 is the main ligand of the ACVRL1/BMPR2/ENG receptor complex in endothelial cells which mediates downstream signaling through phosphorylation of SMAD1/5/8^27,28^. Consistent with published studies, BMP9 increased phosphorylation of SMAD1 in fetal lung endothelial MFLM-91U cells as demonstrated by immunostaining for pSMAD1 (Fig. 7a) and Western blot (Fig. 7b). Furthermore, BMP9 increased ACVRL1 protein levels but did not change the expression of FOXF1 (Fig. 7b). To examine the effect of FOXF1 on BMP9/ACVRL1 signaling in vitro, FOXF1 was inhibited by siRNA prior to BMP9 treatment of MFLM-91U cells (Supplementary Fig. 16a). Inhibition of FOXF1 decreased ACVRL1 and pSMAD1 in BMP9-treated cells but did not affect total SMAD1 (Fig. 7b). There were no changes in pSMAD2 or total SMAD2 (Supplementary Fig. 16b). To examine the consequences of FOXF1 inhibition on transcriptional activity of pSMAD1, we used the BRE-LUC reporter plasmid, which contains a synthetic enhancer element capable of binding to activated pSMAD1^40^. Treatment with BMP9 increased activity of the BRE-LUC reporter (Fig. 7c), consistent with transcriptional activation of pSMAD1. Inhibition of either FOXF1, ACVRL1 or the ACVRL1 co-receptor ENG decreased transcriptional activity of pSMAD1 in BMP9-treated cells (Fig. 7c and Supplementary Fig. 16c). Thus, FOXF1 is required for BMP9/ACVRL1/pSMAD1 signaling in fetal lung endothelial cells in vitro.

Next, we used the in vitro angiogenesis assay to determine whether BMP9 can increase angiogenesis in FOXF1-deficient endothelial cells. Consistent with published studies^19^, siRNA-mediated depletion of FOXF1 inhibited angiogenesis in Matrigel as shown by measurements of the length of endothelial sprouts (Fig. 7d, e) and the number of branching points in the endothelial network (Fig. 7f). Treatment with BMP9 partially restored the ability of FOXF1-deficient endothelial cells to form sprouts in Matrigel (Fig. 7d–f). The effect of BMP9 was completely inhibited by pre-treatment of endothelial cells with ALK1-Fc (Fig. 7d–f), a soluble chimeric protein containing the ACVRL1 ligand-binding domain which functions as a ligand trap for BMP9^28^. Thus, BMP9 increases angiogenesis in FOXF1-deficient lung endothelial cells through the ACVRL1 receptor.

**Fig. 8:**
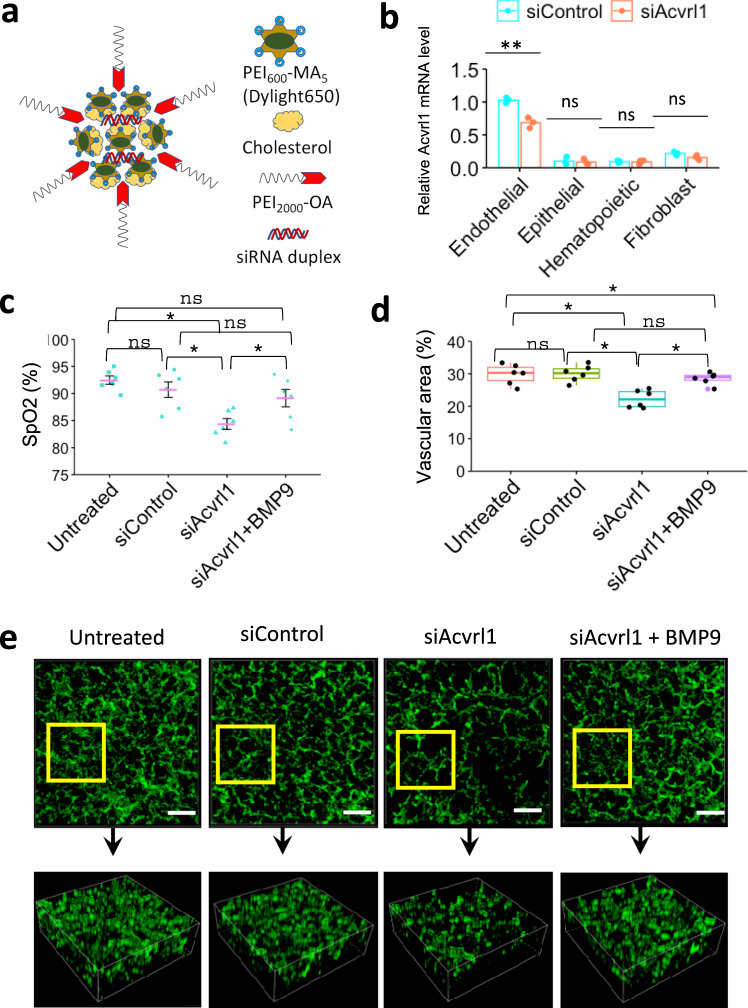
Nanoparticle-mediated inhibition of ACVRL1 decreases neonatal pulmonary angiogenesis. **a** Schematic diagram shows structure of PEI_600_-MA_5_/PEG-OA/Cho nanoparticles containing duplex siRNA, cholesterol, PEI_600_-MA_5_ and PEG_2000_-OA. **b** Real-time PCR analysis shows decreased amounts of *Acvrl1* mRNA in FACS-sorted pulmonary endothelial cells (CD31^+^CD45^–^) from mice treated with nanoparticles containing *Acvrl1*-specific siRNA (siAcvrl1) (*n* = 3). Nanoparticles with scrambled siRNA were used as a control (siControl). Nanoparticles were delivered i.v. to P2 mice. Lungs were harvested at P4. *Acvrl1* mRNA is not changed in FACS-sorted epithelial cells (CD326^+^CD31^–^CD45^–^), fibroblasts (CD140a^+^CD31^–^CD45^–^) and hematopoietic cells (CD45^+^CD31^–^). **c** Arterial oxygen saturation (SpO_2_) is decreased in mice treated with nanoparticles containing *Acvrl1* siRNA compared to control siRNA (*n* = 6). Intravenous BMP9 delivery increases arterial oxygenation in siAcvrl1-treated mice (*n* = 6). Nanoparticles were delivered at P2, BMP9 treatment was performed at P4, arterial oxygenation was measured at P18. **b**, **c** Data were presented as mean ± SD. **d**, **e** Alveolar capillary density is decreased after nanoparticle delivery of *Acvrl1* siRNA but restored after administration of BMP9. Immunostaining lung sections for endothelial marker endomucin (green) and confocal 3D imaging of cleared lung lobes were performed using P18 mouse lungs (*n* = 6). Boxplots: center line, median; box boundary, first and third quartiles; whiskers denote 5th–95th percentile. **b**–**d** Data were shown as mean ± SD, and one-way ANOVA (two-tailed) followed by Tukey’s multiple comparison test was performed. **p < 0.01, *p < 0.05, ns is not significant. Scale bars are 50 μm.

Nanoparticle-mediated inhibition of ACVRL1 in endothelial cells disrupts angiogenesis and alveolarization in the neonatal lung. To determine whether BMP9/ACVRL1 signaling is important for development of pulmonary vasculature after birth, we used Dylight 650-labeled PEI_600_-MA_5_/PEG-OA/Cho nanoparticles (Fig. 8a), which were developed by our group to deliver non-integrating DNA expression plasmids, siRNAs and stabilized mRNAs into the pulmonary microvasculature in vivo^41–44^. Delivery of the nanoparticles containing either *Acvrl1*-specific (siAcvrl1) or control siRNA was performed intravenously in *WT* mice at postnatal day 2 (P2). Forty-eight hours later, the nanoparticles were detected by FACS analysis in approximately 65% of pulmonary endothelial cells (CD31^+^CD45^−^) and 30% of CD140^+^ fibroblasts (CD140^+^CD31^–^CD45^–^CD326^–^) but not in epithelial (CD326^+^CD31^–^CD45^–^CD140^–^) or hematopoietic cells (CD45^+^CD31^–^) (Supplementary Fig. 17a–c). Compared to CD140^+^ fibroblasts, the nanoparticle uptake was significantly higher in pulmonary endothelial cells as demonstrated by the mean fluorescence intensity for Dylight 650 (Supplementary Fig. 17d). Forty-eight hours after nanoparticle delivery of *Acvrl1* siRNA, endogenous *Acvrl1* mRNA was decreased only in endothelial cells but not in other cell types as shown by qRT-PCR analysis of FACS-sorted cells (Fig. 8b). The decrease in endothelial *Acvrl1* mRNA in nanoparticle-treated *WT* mice was approximately similar to that of *Acvrl1* mRNA levels in endothelial cells of *Foxf1*^*WT/S52F*^ mutant mice (Fig. 4a–c), which exhibit impaired lung angiogenesis and ACDMPV^14^. Phosphorylation of SMAD1 was decreased in lungs of mice treated with *Acvrl1* siRNA (Supplementary Fig. 18a), supporting reduced ACVRL1 signaling. Two weeks after the nanoparticle delivery of *Acvrl1* siRNA, arterial oxygenation was decreased (Fig. 8c). Reduced arterial oxygenation in *Acvrl1*-deficient mice was associated with decreased capillary density (Fig. 8d, e and Supplementary Fig. 19a, b) and increased sizes of alveoli (Supplementary Fig. 19c, d), findings consistent with impaired lung angiogenesis and alveolarization. Interestingly, intravenous administration of BMP9 ligand to *siAcvrl1*-treated *WT* mice (Supplementary Fig. 18b) improved arterial oxygenation, increased capillary density, and partially decreased alveolar simplification (Fig. 8c–e and Supplementary Fig. 19a–d). Furthermore, BMP9 treatment restored expression of *Acvrl1* and its downstream target gene *Tmem100* in purified pulmonary endothelial cells from *siAcvrl1*-treated mice (Supplementary Fig. 18c). BPM9 treatment did not change *Foxf1* mRNA in lung endothelial cells (Supplementary Fig. 18c). Thus, BMP9 increases ACVRL1 expression and stimulates endothelial ACVRL1 signaling to promote neonatal lung angiogenesis and alveolarization.

**Fig. 9:**
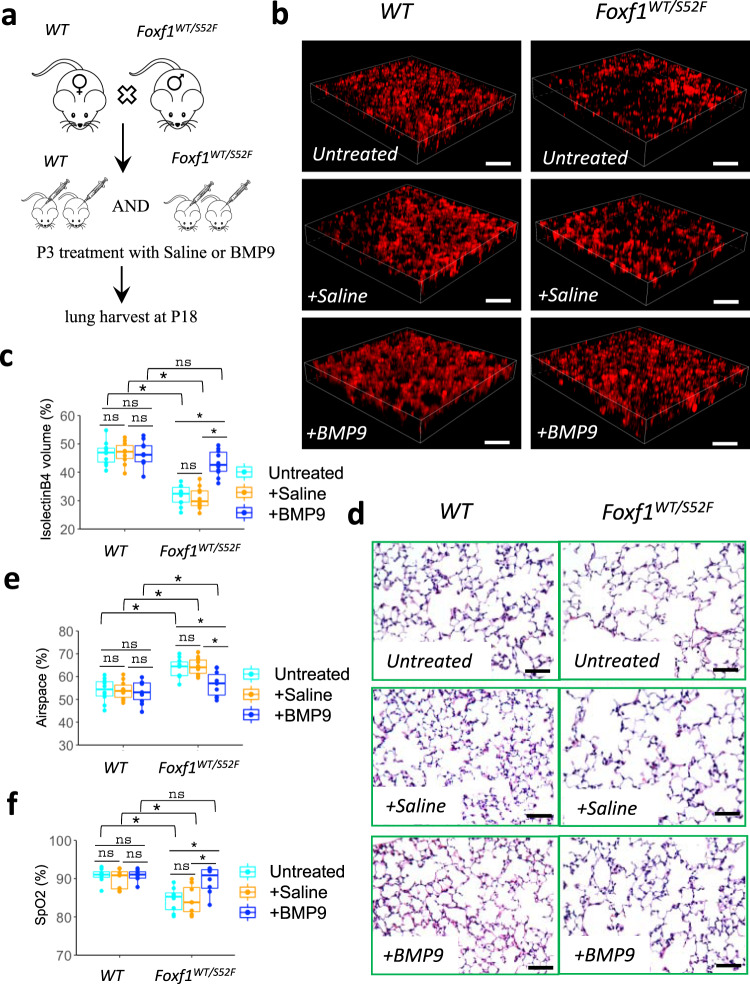
BMP9 treatment increases alveolar capillary density and improves arterial oxygenation in *Foxf1*^*WT/S52F*^ mice. **a** Schematic shows BMP9 treatment in *WT* and *Foxf1*^*WT/S52F*^ littermates. BMP9 was delivered i.v. at P3. Mice treated with saline were used as controls. Lungs were examined at P18. **b**, **c** Delivery of BMP9 improves the capillary density in *Foxf1*^*WT/S52F*^ lungs but have no effect in *WT* controls. Capillary density was measured using the in vivo labeling with isolectin B4 (red) which binds to the luminal surface of perfused blood vessels. Scale bars are 50 μm. Capillary density was quantified using high–resolution confocal microscopy. Ten random images were quantified using *n* = 10 mice in each group. **d**–**e** H&E staining shows that BMP9 treatment protects *Foxf1*^*WT/S52F*^ mice from alveolar simplification. Scale bars are 50 μm. Ten random images were quantified using *n* = 10 mice in each group. **f** BMP9 treatment increases arterial oxygen saturation (SpO_2_) in *Foxf1*^*WT/S52F*^ mice compared to *WT* littermates. Arterial oxygenation was measured at P18 (*n* = 8 mice per group). Boxplots in **c**, **e**, and **f**: center line, median; box boundary, first and third quartiles; whiskers denote 5th–95th percentile. Data were shown as mean ± SD, and one–way ANOVA (two–tailed) followed by Tukey’s multiple comparison test was performed. *p < 0.05,**p < 0.01 ns is not significant.

BMP9 improves neonatal lung angiogenesis and alveolarization in mouse ACDMPV model. Since BMP9 increases angiogenesis in FOXF1-deficient endothelial cells in vitro (Fig. 7d–f), we tested the efficacy of BMP9 treatment on neonatal lung angiogenesis in *Foxf1*^*WT/S52F*^ mouse model of ACDMPV, which is characterized by congenital defects in lung angiogenesis and alveolarization^14^. Since *Foxf1*^*WT/S52F*^ mutant mice with the most severe phenotype die at birth^14^, we used surviving *Foxf1*^*WT/S52F*^ mice with non-lethal congenital defects for BMP9 rescue experiments (Supplementary Fig. 20a, b and^41^). *Foxf1*^*WT/S52F*^ and *WT* littermates were treated with BMP9 ligand or saline at P3 (Fig. 9a). Two weeks later, the mice were used for measurements of arterial oxygenation, histological evaluation, and RNA analysis of the lung tissue. To visualize perfused alveolar capillaries, we performed intravascular labeling with isolectin B4 before the mouse harvest. Compared to either untreated or saline-treated *Foxf1*^*WT/S52F*^ mice, treatment with BMP9 effectively restored the capillary density (Fig. 9b, c), decreased alveolar simplification (Fig. 9d, e), increased arterial oxygenation (Fig. 9f), increased *Acvrl1* expression (Supplementary Fig. 21a), and improved survival in the ACDMPV mouse model (Supplementary Table 6). Treatment with BMP9 had no effect on neonatal lung angiogenesis and alveolarization in *WT* controls (Fig. 9b–f). BMP9 did not change *Foxf1* mRNA in either *WT* or *Foxf1*^*WT/S52F*^ lungs (Supplementary Fig. 21b). Finally, BMP9 treatment did not cause abnormal ossification or ectopic bone formation in the developing mice (Supplementary Fig. 21c). Altogether, administration of BMP9 improves survival and alleviates histological and vascular impairments in lungs of ACDMPV mice.

## Discussion

Recent advances in single cell RNA sequencing identified heterogeneity of endothelial cells that form the alveolar microvasculature^8^. These include aCAPs and gCAPs, the latter of which include EPCs critical for neonatal lung angiogenesis and lung regeneration after injury^8,11^. EPCs are dependent upon expression of FOXF1 transcription factor in these cells^11,12^. Inactivating mutations in the *FOXF1* gene locus are linked to ACDMPV^5,14^, a severe congenital disorder associated with paucity of alveolar capillaries^4^, indicating that FOXF1 is required for development of the alveolar microvasculature. While endothelial cells play a key role in ACDMPV pathogenesis^14^, endothelial cell diversity and key signaling pathways in ACDMPV remain uncharacterized. In this study, we used *Foxf1*^*WT/S52F*^ mice which recapitulate both genetic and pathological findings in ACDMPV patients^14^, to identify the impairment of BMP9/ACVRL1 signaling in *Foxf1*^*WT/S52F*^ EPCs. FOXF1 regulates BMP9/ACVRL1 signaling in EPCs through activation of *ACVRL1* gene expression as evidenced by direct binding of the FOXF1 protein to *ACVRL1* promoter DNA and by transcriptional activation of the *ACVRL1* promoter by *CMV–FOXF1* expression vector. While *ACVRL1* is an evolutionarily conserved transcriptional target of FOXF1 in higher vertebrates, the activation of *ACVRL1* promoter is dependent on transcriptional synergy between FOXF1 and ETS transcription factor FLI1. FOXF1 also synergizes with FLI1 to activate its own promoter region. The presence of functional FOXF1 and FLI1 binding sites in evolutionarily conserved regions of *FOXF1* and *ACVRL1* promoters is consistent with published studies that identified FOX–ETS DNA–binding motifs as a transcriptional signature of endothelial–specific gene expression^45^. Since FLI1 is enriched in FOXF1^+^ EPCs, it is possible that the loss of FOXF1/FLI1 transcriptional synergy (due to the *S52F Foxf1* mutation) is more detrimental for the maintenance of proper *FOXF1* and *ACVRL1* gene expression in FOXF1^+^ EPCs compared to either aCAPs or FOXF1^–^ gCAPs. Our studies suggest a model in which FOXF1 synergizes with FLI1 to increase its own expression and activate the *ACVRL1* promoter, stimulating proangiogenic BMP9/ACVRL1 signaling in pulmonary EPCs (Supplementary Fig. 22). Since decreased expression of *ACVRL1* is found in human ACDMPV, impaired BMP9/ACVRL1 signaling in pulmonary EPCs may contribute to diminished angiogenesis in ACDMPV lungs. Our findings that FOXF1 regulates its own expression through a transcriptional auto–regulatory loop are consistent with published studies demonstrating that FOXF1 functions as a “pioneer” transcription factor in gastrointestinal and myogenic tumor cells^46,47^.

Hereditary hemorrhagic telangiectasia (HHT) is a genetic disorder characterized by mucocutaneous telangiectases, visceral arteriovenous malformations and hemorrhage^26^. HHT is caused by abnormal TGFβ/BMP9 signaling in endothelial cells through ACVRL1 and its co–receptor ENG^24,25^. Vascular abnormalities and hemorrhage in HHT type 1 result from inactivating mutations in *ENG*, whereas vascular defects in HHT type 2 are caused by mutations in *ACVRL1*^24,25^. ACVRL1 binds to ENG and BMPR2 to form a receptor complex, which promotes TGFβ/BMP signaling through phosphorylation of SMAD1, SMAD5 or SMAD8 that translocate into the nucleus to regulate gene expression^27^. Heterozygous germline mutations in BMPR2 are linked to a heritable form of pulmonary arterial hypertension (PAH)^48^. Since ACDMPV is associated with PAH and lung hemorrhage^4^, it is not surprising that BMP9/ACVRL1/ENG signaling is impaired in ACDMPV. However, obvious differences in pathological and clinical findings between HHT, PAH and ACDMPV suggest that mutations in human *ACVRL1*, *BMPR2, ENG* and *FOXF1* have unique molecular mechanisms, and perhaps, affect different cell types in addition to regulating BMP9/ACVRL1/ENG signaling in mature endothelial cells and EPCs. Identification of common and unique molecular mechanisms for each mutation will require single cell genomic, transcriptomic, and proteomic approaches using human lung biopsies. Our scRNAseq analysis of murine embryonic lungs demonstrates that *Acvrl1*, *Bmpr2, Eng* and *Foxf1* are expressed in multiple pulmonary cell types, supporting the potential involvement of these genes in many cellular processes and cell–to–cell interactions.

An important contribution of our study is that i.v. administration of BMP9 ligand stimulates neonatal lung angiogenesis and alveolarization in ACDMPV mice, suggesting that BMP9 agonists may be considered for treatment of human ACDMPV. BMP9 overcomes FOXF1-mediated defects in angiogenesis in *Foxf1*^*WT/S52F*^ mutant mice and in vitro by increasing ACVRL1 expression and activating BMP9/ACVRL1/SMAD1 signaling in pulmonary EPCs. Our results are consistent with published studies demonstrating that administration of BMP9 protects the lung from alveolar simplification caused by neonatal hyperoxic injury^30^, and alleviates pulmonary hypertension in *Bmpr2*-mutant mice^29^. Interestingly, BMP9 treatment did not cause aberrant angiogenesis in *WT* lungs, findings consistent with published studies in which administration of BMP9 had no adverse effects in adult *WT* mice and rats^29^. It is possible that short duration of BMP9 stimulation is insufficient to cause aberrant angiogenesis during normal lung development. It is also possible that *Foxf1*^*WT/S52F*^ mice are more sensitive to BMP9 stimulation in EPCs due to the presence of a “vascular niche” created by microvascular deficiency in FOXF1-deficient lungs.

In summary, the *S52F Foxf1* mutation disrupts BMP9/ACVRL1 signaling in pulmonary EPCs, causing decreased neonatal lung angiogenesis and alveolarization in *Foxf1*^*WT/S52F*^ mice. BMP9/ACVRL1 signaling is decreased in human ACDMPV lungs. FOXF1 synergizes with FLI1 to stimulate *ACVRL1* gene transcription through an evolutionarily conserved −538/−458 bp promoter region. Administration of BMP9 ligand to either ACVRL1-deficient or *Foxf1*^*WT/S52F*^ mice restores neonatal lung angiogenesis and alveolarization. Our studies demonstrate that pulmonary EPCs stimulate neonatal lung angiogenesis and alveolarization through FOXF1-mediated activation of BMP9/ACVRL1 signaling.

## Methods

### Ethics Statement

The data, analytic methods, and study materials will be made available upon request from the corresponding author of this manuscript to other researchers for purposes of reproducing the results or replicating the procedures. All animal studies were approved by the Institutional Animal Care and Use Committee of the Cincinnati Children’s Hospital. Newborn mouse pups delivered on the same day were randomized at day of birth at P0 and divided to equal–sized litters of 4. Pups were euthanized by injection of pentobarbital sodium at 10 μl/kg. All mice were kept under SPF (specific-pathogen free) conditions in 12/12 light/dark cycle, 18–23 ^o^C and 40–60% humidity. The NIH guidelines for laboratory animal care were strictly followed.

### Integrative analysis of single cell RNA sequencing of the mouse lung

Single cell RNAseq libraries were generated from *Foxf1*^*WT/S52F*^ and *WT* E18.5 lungs using the GemCode Single-Cell Instrument and Single Cell 3’ Library & Gel Bead Kit v3 and Chip Kit (10x Genomics) according to manufacturer’s protocol. The lungs were finely minced into small pieces on ice in RPMI-1640 medium containing 0.2 mg/ml Liberase TM (Roche) and 100U/ml Deoxyribonuclease I (Sigma). Lung tissues were digested for 30 min at 37 ^o^C. After digestion, cell suspensions were passed through a 70 μm cell strainer (Corning). Red blood cells were lysed in Ammonium-Chloride-Potassium (ACK) lysis buffer (Thermofisher). Hematopoietic cells were depleted from the cell suspensions using mouse CD45 magnetic beads in MS columns. Cell viability was determined in hemocytometer chambers using trypan blue stain. Equal numbers of cells from three *Foxf1*^*WT/S52F*^ and *WT* E18.5 lungs were combined to prepare sequencing libraries. Approximately 11,000 cells were loaded for each sample with a targeted cell recovery estimate of 7,000 cells. The libraries were sequenced with Novaseq 6000 at a sequencing depth of ~50,000 reads per cell. Starting with BCL files obtained from Illumina sequencing, we used *cellranger mkfastq* to extract sequence reads in FASTQ format. The *cellranger count* with mm10 reference genome (as provided by 10X) and the Cell Ranger software (v3.0.0, 10X Genomics) were used to generate gene-count matrices from the FASTQ files. Reads were processed by counting Unique Molecular Identifiers (UMI). Cells with unique feature counts less than 500 and more than 8000 were excluded from the analysis. We also excluded cells that had more than 20% mitochondrial counts. Filtering, normalization criteria and principal component analysis of scRNAseq datasets are described in^12,32^.

For integrative analysis of *WT* and *Foxf1*^*WT/S52F*^ cells, the combined dataset was analyzed by the Seurat software (version 3.0 in R3.5 environment). For both *WT* and *Foxf1*^*WT/S52F*^ cells, the gene-feature matrices were pre-processed using the same procedures and criteria. The data were log-normalized with a scale factor of 10^4^. The outlier cells were regressed out based on the percentage of mitochondrial reads and the number of UMIs in a negative binomial model. Variable features were determined by the *FindVariableGenes* function using the Seurat package. *FindIntegrationAnchors* and *IntegrateData* functions were used to integrate *WT* and *Foxf1*^*WT/S52F*^ datasets. Next, highly variable features from the integrated matrix were used as input for linear dimensionality reduction via principal component analysis (PCA). Principal components (PCs) were examined using functions *ElbowPlot* and *DimHeatmap* to determine the number of PCs for downstream analysis. PCs with standard deviation > 3.5 were used as input for unsupervised clustering with a resolution set to 0.6, using the nonlinear UMAP down dimensional algorithm.

For differential analysis of gene expression in gCAPs, *WT* and *Foxf1*^*WT/S52F*^ gCAPs were defined using the *Idents* function in the Seurat package. Next, the *FindMarkers* function was used with the *logfc* threshold set to 0.4 to identify genes downregulated or upregulated between *WT* and *Foxf1*^*WT/S52F*^ gCAPs. For regression analysis, cells from the cluster AT1 and Matrix. FB -2 were selected and separated based on the *orig.ident* function from the meta.data. The heatmaps were generated using the function *DoHeatmap* in the same package. The gene expression signatures were separately produced for *WT* and *Foxf1*^*WT/S52F*^ datasets using the *AverageExrpression* function and normalized using the *log1p* function. The linear regression plot was generated using the *ggplot2* package. For molecular pathway analysis, the functional annotation clustering was performed using *DAVID* Bioinformatics Resources Ver.6.8. The KEGG (Kyoto Encyclopedia of Genes and Genomes) pathway enrichment analysis was used to produce the annotation data. Functional clusters with Benjamini-corrected *P* value <  0.05 were considered statistically significant. Heatmap were generated using the *pheatmap* package.

### FACS analysis and cell sorting

Lungs were perfused through the right ventricle with 5 ml of PBS to remove blood. The lung tissue was enzymatically digested using 0.2 mg/ml Liberase TM (Roche) and 100U/ml Deoxyribonuclease I (Sigma, DN25) for 45 mins, and then passed through nylon mesh (70 µm pores) to obtain a single cell suspension as described^42,49,50^. Red blood cells were lysed in ACK lysis buffer. To remove cell debris, single cell suspensions from the lung tissue were passed through cell strainer snap cap (Corning life sciences). Single cell suspensions were stained with fixable viability dye (Biolegend) followed by the incubation with TruStain FcX (Biolegend). To identify cell surface antigens, cells were stained with a mixture of fluorochrome-conjugated antibodies (Supplementary Table 7). Data were acquired using BD LSR II cytometer and FACSDiva 8.0 software. Compensation and data analyses were performed using FlowJo software (TreeStar version 10.5.1). The *Foxf1-GFP* reporter line containing *GFP* knocked into the endogenous *Foxf1* gene locus was used to purify FOXF1^+^ gCAPs. Stained cells were separated using the five-laser FACS Aria II cell sorter (BD Biosciences). Sorted cells were first collected into EGM-2 medium (Lonza), and then washed once with the medium after centrifugation at 300 *g* for 5 mins.

### RNA preparation and quantitative real-time RT-PCR

RNeasy micro plus kit (Qiagen) was used to prepare total RNA from endothelial cells (CD31^+^CD45^–^) that were purified through either FACS sorting or immunomagnetic beads. For the immunomagnetic method, the purity of CD31^+^CD45^–^ cell enrichment was ~85% as determined by flow cytometry. Quantitative real-time RT-PCR (qRT-PCR) was performed using a StepOnePlus Real-Time PCR system (Thermofisher) as described previously^51,52^. Samples were amplified using inventoried TaqMan primers (Supplementary Table 8). Reactions were analyzed in triplicates, and expression levels were normalized to *β-actin* mRNA.

### DNA constructs and dual luciferase assay

ACE400 and ACE80 *Acvrl1* promoter regions were generated by PCR from mouse genomic DNA and cloned into the pGL4.25 luciferase (LUC) reporter plasmid. PCR primers are provided in Supplementary Table 9. The 400 bp fragment of the *Acvrl1* promoter region (ACE400) was generated using a 5′ primer containing a *KpnI* site, and a 3′ primer containing a *NheI* site. The amplified ACE400 DNA was purified and cloned into the *KpnI-NheI* linearized *pGL4.25* LUC reporter plasmid containing a minimal promoter. The ACE80 LUC reporter was produced by directly annealing two oligos: 5′ oligo containing a *KpnI* site, and a 3′ oligo containing a *NheI* site. The quickchange site-directed mutagenesis was used to disrupt FOXF1-binding sites, and the primers used were listed in Supplementary Table 9. All constructs were verified by DNA sequencing. CMV-FOXF1, CMV-S52F FOXF1 and CMV-empty plasmids were generated previously^14,53,54^. Expression constructs for ETS transcription factors were obtained from Addgene. Dual LUC assay was performed using the standard protocol from Promega in mouse fetal lung endothelial MFLM-91U cells as described^47,55^.

### RNAscope assay

Mouse lungs were fixed in 4% paraformaldehyde for 24 hr at 4 ^o^C. Tissue sections were cut at 12 μm and the staining was performed using the RNAscope Multiplex Fluorescent Reagent Kit (v.2) and Opal Fluorophore reagents (Akoya Biosciences, 1:750 dilution for Opal 570 and 1:500 dilution for 690 dyes). Sections were counterstained with DAPI and mounted in the Prolong Gold antifade reagent (Thermofisher). The following riboprobes from Advanced Cell Diagnostics were used: Mm-Foxf1 (473051), Mm-Acvrl1(319031), Mm-Tmem100 (435201) and Mm-Aplnr (436171-C2).

### Cell culture and immunostaining

The mouse fetal lung mesenchymal MFLM-91U cell line was obtained from Seven Hill Bioreagents. MFLM-91U cells were transfected with siRNAs or treated with recombinant BMP9 (25 ng/ml, R&D) and harvested 48 h after the treatment. After removal of culture medium by aspiration, the cells were washed with PBS and then fixed in 4% paraformaldehyde (PFA) for 15 mins. Cells were permeabilized with 0.1% Triton-X100 and incubated in blocking buffer (10% fetal bovine serum in PBS). The cells were stained with primary antibodies overnight at 4 °C, followed by 1-h incubation with Alexa-488 or Alexa-594-conjugated donkey anti-rabbit IgG (1:1000, Invitrogen, A-21207) and donkey anti-rat IgG (1:1000, Invitrogen A-21208). Cell nuclei were counterstained with Hoechst 33258 (Thermofisher). Images were acquired using Nikon Eclipse Ti2 inverted microscope. Lung cryosections were either stained with Hematoxylin and Eosin (H&E) or used for immunostaining as described^55–58^. Antibodies for immunostaining are listed in Supplementary Table 7 and the staining conditions are described in^59–62^. Morphometrical measurements of alveolar sizes and intra-vascular labeling with isolectin B4 were described in published studies^14,63,64^. Co-localization studies and confocal imaging of lung tissue sections were performed as described^65–67^.

### In vitro angiogenesis in Matrigel

Fetal lung endothelial MFLM-91U cell line was maintained in serum free medium. For angiogenesis assay, 150 µl of growth factor-reduced Matrigel was placed in each well of 8-well cell culture chamber slides (Corning), pre-incubated on ice, and then incubated for 1 h at 37 ^o^C. For each well, 40,000 cells were seeded and incubated in preconditioned media supplemented with recombinant BMP9 protein (25 ng/ml, R&D). Alk1-Fc protein (R&D, 770-MA) was used to inhibit the effect of BMP9/ACVRL1 signaling. To visualize endothelial sprouts, cells were stained with calceinAM fluorescent viability dye at a final concentration of 2 µg/ml for 30 min. The slides were examined and analyzed using Nikon Eclipse Ti2 inverted microscope and NIS Elements AR software.

### Nanoparticle-mediated inhibition of *Acvrl1* in the mouse neonatal lung

C57BL/6 mice were purchased from Jackson laboratory. Postnatal day 2 (P2) mice were intravenously (i.v.) injected with PEI_600_-MA_5_/PEG-OA/Cho nanoparticles carrying *Acvrl1*-specific siRNA or control siRNA (5 μg in a total volume of 25 μl) through the facial vein. Chemical synthesis of PEI_600_-MA_5_/PEG-OA/Cho nanoparticles is described in^14,42,43^. BMP9 ligand (R&D) was injected i.v. at P4 via the facial vein (50 ng per mouse in 25 μl of saline).

PEI_600_-MA_5_/PEG-OA/Cho nanoparticles were used to encapsulate siRNA duplexes. Briefly, PEI_600_ was conjugated with myristic acid to form PEI_600_-MA_5_. PEG2k was conjugated with oleic acid to form PEG2k-OA. To form NHS esters, carboxylate groups were activated by EDC/NHS for 15 min at 40 ^o^C in 95% ethanol buffered with 25 mM MES (pH 6.0). PEI or PEG-NH_2_ were quickly added following NHS esters. The mixture was incubated overnight at 40 ^o^C to form PEI_600_-MA_5_ or PEG-OA. Residual chemicals in PEI_600_-MA_5_ or PEG-OA solutions were removed by dialysis against 90% ethanol or water, respectively. PEI_600_-MA_5_, PEG-OA and cholesterol were mixed in ethanol at a mass ratio of 90:10:10. The residual solvent was removed by rotary evaporation to produce a lipid mixture. To label the nanoparticles, the DyLight 650-NHS ester fluorescence dye was coupled to the amine groups on the nanoparticles (1:100 dye to nanoparticle mass ratio). The lipid mixture was resuspended in 25 mM HEPES buffer (pH 7.4). The siRNA stock solution (100 μM) was used to prepare siRNA-encapsulated lipid nanocomplexes at a polymer/siRNA ratio of 20:1 (wt/wt). Nanoparticle complexes with 5 nmol of encapsulated siRNA were diluted in 25 μl carrier solution (25 mM HEPES buffer and 5% glucose) and injected into P2 mice via the facial vein. For BMP9 treatment in vivo, the dose 50 ng/mouse of recombinant BMP9 (5566-BP/CF, R&D) was injected i.v. to nanoparticle-treated P4 mice. Recombinant BMP9 was prepared as 100 μg/ml stock solution. The stock solution was diluted and kept on ice immediately prior to the BMP9 administration.

### BMP9 treatment of *Foxf1*^*WT/S52F*^ mice and measurements of capillary density using the whole mount lung imaging

*Foxf1*^*WT//S52F*^ and *Foxf1*^*WT-GFP/+*^ mouse lines were described previously^11,14^. *Foxf1*^*WT//S52F*^ mice were maintained in C57BL/6 × 129/J genetic background^14^. Mice were anesthetized for in vivo treatment with recombinant BMP9 protein (R&D). At P2, *Foxf1*^*WT/S52F*^ and *WT* littermates were given i.v. injections of recombinant BMP9 (50 ng/mouse) via the facial vein. Lungs were harvested at P18. Alexa fluor 594-conjugated IsolectinB4 (Thermofisher) was injected i.v. one hour prior to lung harvest to label pulmonary vasculature. Lung was perfused with PBS, inflated with 4% PFA and fixed in 4% PFA overnight. Fixed tissues were washed with PBS three times and incubated in the hydrogel monomer solution A4P0 (4% acrylamide in PBS) supplemented with 0.25% photoinitiator 2,2ʹʹ-Azobis(2-(2-imidazolin-2-yl) propane) dihydrochloride (VA-044, Wako Chemicals, USA) for two days, and then incubated for 6 h at 37 °C to initiate tissue-hydrogel hybridization. After removing excess of hydrogel by washing the tissues with PBS, tissues were cleared with X-clarity system (Logos biosystems) in electrophoretic tissue clearing solution for 6 h. After several washes in PBS for two days, the samples were transferred to RIMS to align the refractive indices of the objective. RIMS consists of 88% w/v Histodenz (D2158, Sigma-Aldrich), pH adjusted to 7.5 with NaOH. Samples were incubated in RIMS overnight and then mounted in fresh RIMS for imaging. The 3D images of pulmonary vasculature were acquired using a NIKON multiphoton confocal microscope. For each treatment, 10 random lung images from 3–5 mice were used for quantification of the microvascular network. The images were captured as Z-series stack containing 11 slices (9 μm thickness). Confocal images were quantified using NIS elements and ImageJ software.

### Measurements of arterial oxygen saturation

Arterial oxygenation was measured using *MouseOx* + pulse oximeter (STARR Life Sciences). The *MouseOx* + photodiode sensor was placed on the shaved skin of the neck, and the measurements were taken for 10 min during stable heart rate and oxygen saturation readings.

### Western blot

MFLM-91U cells were transfected in six-well plates and harvested 48 h after the transfection. After rinsing cells with ice-cold PBS, cells were lysed in cold RIPA buffer (Cell Signaling Technology) supplemented with protease and phosphatase inhibitors. The protein concentrations were determined using Bio-Rad DC assays. Western blot analysis was performed as described^68–70^. Briefly, cell lysates (30 μg of total protein) were separated on 4–12% gradient gels and transferred to PVDF transfer membranes (0.2 μm) (Thermo Scientific) using a wet transfer. After blocking with Odyssey blocking buffer (Li-Cor Biosciences), membranes were incubated with primary antibodies overnight at 4 °C. The membranes were washed 3 times for 10 mins with TBS-T and incubated for 1 h with secondary antibodies from Li-Cor Biosciences. The membranes were washed 3 times for 10 mins with TBS-T and imaged using LI-COR Odyssey CLx.

### Statistical analysis

Statistical analyses were performed using R software and Graphpad Prism 8.0. One-way ANOVA and Student’s *T*-test were used to determine statistical significance. *P* < 0.05 were considered statistically significant. Multiple means were compared using one-way analysis of variance with the post-hoc Tukey’s test when comparing the groups for which a post hoc analysis of each group was required. Non-parametric Mann-Whitney U test was used for datasets with *n* < 6 to determine statistical significance. Values were presented as mean ± standard deviation (SD).

## Supplementary information

Supplementary information

## Data Availability

Single cell RNA sequencing data generated in this study from WT and *Foxf1*^*WT/S52F*^ E18.5 lungs were deposited in GEO database (accession number GSE178184). FOXF1 ChIPseq data from mouse lungs (GSE77951; https://www.ncbi.nlm.nih.gov/geo/query/acc.cgi?acc=GSE54780) and FLI1 ChIPseq (GSE69101) are also available in a public database (http://cistrome.org/db/). Microarray data from ACDMPV lungs were retrieved from GEO database (GSE54780) using GEOquery 2.5.6 R package. The data were analyzed by the R package limma (3.8.3). Source data are provided with this paper.

## Source data

Source Data

## Code availability

Code used for the analysis of scRNAseq data is available in a public repository at https://github.com/WGLUN/acdmodel.
