# Supplementary material for: Endothelial progenitor cells stimulate neonatal lung angiogenesis through FOXF1-mediated activation of BMP9/ACVRL1 signaling
Source: Nat Commun. 2022 Apr 19;13:2080. doi: 10.1038/s41467-022-29746-y (PMC9019054; doi:10.1038/s41467-022-29746-y)
Supplement: Supplementary file 1 — Supplementary information [file 41467_2022_29746_MOESM1_ESM.pdf]

SUPPLEMENTAL MATERIALS

Endothelial Progenitor Cells Stimulate Neonatal Lung Angiogenesis through FOXF1-Mediated Activation of BMP9/ACVRL1 Signaling

Guolun Wang[1], Bingqiang Wen[1], Zicheng Deng[1,2], Yufang Zhang[1], Olena A. Kolesnichenko[1], Vladimir Ustiyan[1], Arun Pradhan[1], Tanya V. Kalin[3,4] and Vladimir V. Kalinichenko[1,3,4,5]

[1]*Center for Lung Regenerative Medicine, Perinatal Institute, Cincinnati Children's Hospital Medical Center, Cincinnati, USA.*
[2]*The Materials Science and Engineering Program, College of Engineering and Applied Science, University of Cincinnati, Cincinnati, USA.*
[3]*Division of Pulmonary Biology, Cincinnati Children's Hospital Medical Center, Cincinnati, USA.*
[4]*Department of Pediatrics, University of Cincinnati, Cincinnati Children's Hospital Medical Center, Cincinnati, USA.*
[5]*Division of Developmental Biology, Cincinnati Children's Hospital Medical Center, Cincinnati, USA.*

SUPPLEMENTAL TABLES

Supplemental Table 1. Mouse genes with decreased expression in *Foxf1*$^{WT/S52F}$ gCAPs compared to *WT* gCAPs, Wilcoxon Rank Sum test implemented via the function FindMarkers in Seurat package was used for data analysis.

Genes	Annotation	p_val_adj	avg_logFC
Scn7a	sodium voltage-gated channel alpha 7	1.26E-05	-1.1595084
Calcrl	calcitonin receptor like receptor	1.72E-04	-1.041414
Id1	Id1	6.47E-06	-0.7609647
Ace	angiotensin I converting enzyme	6.46E-04	-0.7324971
Prx	periaxin	1.37E-02	-0.7135365
Aqp1	aquaporin 1	1.85E-03	-0.6940737
Impdh1	inosine monophosphate dehydrogenase 1	1.02E-02	-0.6768223
Guk1	guanylate kinase 1	4.24E-02	-0.6750678
Clec14a	C-type lectin domain containing 14A	7.80E-05	-0.6744908
Emilin1	elastin microfibril interfacer 1	6.60E-03	-0.6699169
Thbd	thrombomodulin	1.00E-03	-0.6689993
Akap5	A-kinase anchoring protein 5	3.00E-04	-0.6643067
Icam2	intercellular adhesion molecule 2	1.44E-04	-0.662177
Bcam	basal cell adhesion molecule	1.73E-02	-0.6497749
Pltp	phospholipid transfer protein	3.71E-04	-0.6299058
Gja4	gap junction protein alpha 4	4.64E-02	-0.5994817
Pmp22	peripheral myelin protein 22	8.97E-04	-0.5932426
Acvrl1	activin A receptor like type 1	1.56E-03	-0.5744891
Pcdh1	protocadherin 1	9.40E-03	-0.5699518
Pgrmc1	progesterone receptor membrane component 1	1.22E-02	-0.5422058
Emc3	ER membrane protein complex subunit 3	8.14E-03	-0.5385768
Hilpda	hypoxia inducible lipid droplet associated	7.56E-02	-0.5359901
Kitl	KIT ligand	5.83E-02	-0.5286402
Stmn2	stathmin 2	7.81E-03	-0.5242799
Serinc3	serine incorporator 3	3.45E-03	-0.523888
Bcap31	B cell receptor associated protein 31	1.71E-02	-0.5194915
Agrn	agrin	5.60E-03	-0.5180866
Adgre5	adhesion G protein-coupled receptor E5	1.38E-02	-0.5159666
Mpzl1	myelin protein zero like 1	1.76E-02	-0.5081411
Erlec1	endoplasmic reticulum lectin 1	1.76E-02	-0.5072873
Id3	Id3	2.46E-02	-0.5062088

Abhd17c	depalmitoylase	5.64E-03	-0.4982948
Nid1	nidogen 1	2.74E-02	-0.4968771
Ehd4	EH domain containing 4	3.18E-02	-0.4946315
Tmem2	Hyaluronidase	2.05E-02	-0.493116
Rdx	radixin	8.95E-03	-0.4904556
Rcn2	reticulocalbin 2	3.77E-03	-0.487199
Ptprm	protein tyrosine phosphatase receptor type M	2.07E-02	-0.4857461
Cdh5	cadherin 5	2.28E-02	-0.484025
Tbx2	T-box transcription factor 2	1.72E-02	-0.4831394
Cav2	caveolin 2	1.95E-02	-0.4822263
Stab1	stabilin 1	3.90E-02	-0.479902
Cldn3	claudin 3	3.76E-02	-0.4795833
Lyz2	Lysozyme C-2 precursor	1.91E-03	-0.4795814
Rpn2	ribophorin II	3.04E-02	-0.4777088
Slc16a2	solute carrier family 16 member 2	1.76E-02	-0.4760005
Tspan18	tetraspanin 18	1.84E-02	-0.4740073
Saraf	calcium entry associated regulatory factor	1.18E-02	-0.4735024
Tmem176b	transmembrane protein 176B	9.02E-03	-0.4676268
Tor1aip1	torsin 1A interacting protein 1	2.24E-02	-0.4667835
Tspan8	tetraspanin 8	2.17E-02	-0.4640212
Daam1	dishevelled associated activator of morphogenesis 1	2.36E-02	-0.4636584
Casz1	castor zinc finger 1	1.46E-02	-0.4632158
Acap2	ArfGAP with coiled-coil, ankyrin repeat and PH domains 2	2.00E-02	-0.4594823
Cpne8	copine 8	2.50E-02	-0.4582557
Arl8b	ADP ribosylation factor like GTPase 8B	1.19E-02	-0.4569753
Ptpn4	protein tyrosine phosphatase non-receptor type 4	2.74E-03	-0.4567502
Lfng	LFNG O-fucosylpeptide acetylglucosaminyltransferase	2.01E-02	-0.4546993
Itga1	integrin subunit alpha 1	2.56E-02	-0.4539738
Podxl	podocalyxin like	3.02E-02	-0.4539606
Sar1b	secretion associated Ras related GTPase 1B	2.27E-02	-0.4538727
Armcx4	armadillo repeat containing X-linked 4	2.36E-02	-0.4536357
Tceal8	transcription elongation factor A like 8	5.21E-02	-0.4510441
Ptgfrn	prostaglandin F2 receptor inhibitor	7.06E-02	-0.4500174
Yipf3	Yip1 domain family member 3	2.15E-02	-0.4483878
Fam189a2	family with sequence similarity 189 member A2	2.71E-02	-0.4459024
Sftpd	surfactant protein D	3.71E-02	-0.4445538
Krt18	keratin 18	1.94E-02	-0.443645
Tm4sf1	transmembrane 4 L six family member 1	4.46E-02	-0.4435192
Plin2	perilipin 2	4.97E-02	-0.4384208

Tmem213	transmembrane protein 213	1.99E-02	-0.4374779
Pam	peptidylglycine alpha-amidating monooxygenase	6.17E-03	-0.4371594
Faf1	Fas associated factor 1	1.93E-02	-0.4364524
Hsd17b4	hydroxysteroid 17-beta dehydrogenase 4	3.74E-02	-0.4320081
Cd151	CD151 molecule	5.03E-02	-0.4304046
S100a14	S100 calcium binding protein A14	1.27E-02	-0.4297003
Dll4	delta like canonical Notch ligand 4	8.92E-02	-0.4276322
F11r	F11 receptor	1.38E-02	-0.4262241
Chchd10	coiled-coil-helix-coiled-coil-helix domain containing 10	5.32E-02	-0.4254025
Ecscr	endothelial cell surface and apoptosis regulator	5.67E-03	-0.4225633
Tmem100	transmembrane protein 100	4.32E-04	-0.4182826
Ly6e	lymphocyte antigen 6 family member E	2.39E-03	-0.4177506
Kctd10	potassium channel tetramerization domain containing 10	1.57E-02	-0.4173224
Ramp2	receptor activity modifying protein 2	5.21E-04	-0.4162491
Colgalt1	collagen β-galactosyltransferase 1	5.18E-02	-0.4122362
Tmed10	transmembrane p24 trafficking protein 10	2.87E-02	-0.4121418
Nradd	Death domain-containing membrane protein NRADD	5.21E-03	-0.4115611
Tfpi	tissue factor pathway inhibitor	1.74E-02	-0.4100548
Pbxip1	PBX homeobox interacting protein 1	3.45E-02	-0.4094844
Mif4gd	MIF4G domain containing	1.54E-02	-0.4086005
Eif3m	eukaryotic translation initiation factor 3 subunit M	3.94E-02	-0.408302
B2m	β-2-microglobulin	2.16E-02	-0.4065702

Supplemental Table 2. Mouse genes with increased expression in *Foxf1*$^{WT/S52F}$ gCAPs compared to *WT* gCAPs, Wilcoxon Rank Sum test implemented via the function FindMarkers in Seurat package was used for data analysis.

**Genes**	**Annotation**	p_val_adj	avg_logFC
Ndnf	neuron derived neurotrophic factor	2.16E-03	0.76654559
Rbp7	retinol binding protein 7	1.48E-03	0.76318541
Rbp1	retinol binding protein 1	7.07E-06	0.72806533
Pnisr	PNN interacting serine and arginine rich protein	6.02E-05	0.69723989
Ppp1r14a	protein phosphatase 1 regulatory inhibitor subunit 14A	7.01E-06	0.69127027
Kmt2e	lysine methyltransferase 2E	5.94E-06	0.66629169
Ebf1	EBF transcription factor 1	4.55E-03	0.65330788
Tnrc6a	trinucleotide repeat containing adaptor 6A	5.08E-05	0.64319892
Taf1	TATA-box binding protein associated factor 1	1.77E-02	0.64298204
Ubn2	ubinuclein 2	8.96E-07	0.63664304
3830406C13Rik	chromosome 3 open reading frame 14	1.32E-03	0.62265351
Sgk1	serum/glucocorticoid regulated kinase 1	1.13E-04	0.61928485
Col1a1	collagen type I alpha 1 chain	7.06E-03	0.61336275
Rragc	Ras related GTP binding C	1.68E-05	0.6092144
Brd4	bromodomain containing 4	2.15E-04	0.60900764
Srrt	serrate, RNA effector molecule	1.30E-03	0.6026708
Supt20	SPT20 homolog, SAGA complex component	8.42E-03	0.59879275
Bola2	bolA family member 2	6.65E-05	0.59853836
Kcnq1ot1	KCNQ1 opposite strand/antisense transcript 1	4.43E-03	0.59852063
Klhl9	kelch like family member 9	1.07E-04	0.59139999
Sh3bgrl	SH3 domain binding glutamate rich protein like	4.90E-04	0.58110633
Safb2	scaffold attachment factor B2	1.11E-03	0.57904685
Chd2	chromodomain helicase DNA binding protein 2	5.77E-04	0.57751316
Tcf7l1	transcription factor 7 like 1	2.59E-03	0.57257241
N4bp2l2	NEDD4 binding protein 2 like 2	7.74E-04	0.56730607
Prrc2c	proline rich coiled-coil 2C	2.80E-05	0.56160247
Utrn	utrophin	6.07E-03	0.56091269
Coq7	coenzyme Q7, hydroxylase	6.92E-04	0.56073223
Srrm2	serine/arginine repetitive matrix 2	8.69E-04	0.55116625
Tef	TEF transcription factor, PAR bZIP family member	2.85E-02	0.54956929
Palm	paralemmin	5.04E-04	0.5486286
Dusp1	dual specificity phosphatase 1	8.94E-04	0.54594957
Adamts9	ADAM metallopeptidase with thrombospondin motif 9	4.75E-03	0.54253029

Dcaf13	DDB1 and CUL4 associated factor 13	2.39E-03	0.5419161
Ifitm1	interferon induced transmembrane protein 1	8.16E-03	0.53890677
Zfp207	zinc finger protein 207	4.14E-03	0.53345352
Tmpo	thymopoietin	1.73E-03	0.53248504
Zc3h15	zinc finger CCCH-type containing 15	9.95E-06	0.52910164
Pfdn2	prefoldin subunit 2	1.94E-04	0.5289352
Dab2ip	DAB2 interacting protein	2.38E-02	0.52542226
Phb2	prohibitin 2	3.32E-03	0.52272165
Limch1	LIM and calponin homology domains 1	1.56E-04	0.52014723
Atg101	autophagy related 101	2.58E-03	0.51826269
Sqstm1	sequestosome 1	1.24E-02	0.51372186
Pcbd2	pterin-4 alpha-carbinolamine dehydratase 2	6.22E-02	0.51081317
Tmem255a	transmembrane protein 255A	3.21E-04	0.51041205
Snhg9	small nucleolar RNA host gene 9	1.20E-03	0.50963362
Srp72	signal recognition particle 72	6.92E-02	0.50167931
Fkbp8	FKBP prolyl isomerase 8	4.54E-04	0.5008499

Supplemental Table 3. Mouse genes from TGFβ/BMP signaling pathway expression of which is downregulated in *Foxf1*$^{WT/S52F}$ gCAPs, Wilcoxon Rank Sum test implemented via the function FindMarkers in Seurat package was used for data analysis.

**genes**	**Annotation**	**p_val_adj**	**avg_logFC**
Acvrl1	activin A receptor like type 1	1.56E-03	-0.5744891
Calcrl	calcitonin receptor like receptor	1.72E-04	-1.041414
Clec14a	C-type lectin domain containing 14A	7.80E-05	-0.6744908
Emilin1	elastin microfibril interfacer 1	6.60E-03	-0.6699169
Id1	Inhibitor of DNA Binding 1	6.47E-06	-0.7609647
Id3	Inhibitor of DNA Binding 3	2.46E-02	-0.5062088
Pcdh1	protocadherin 1	9.40E-03	-0.5699518
Prx	periaxin	1.37E-02	-0.7135365
Stmn2	stathmin 2	7.81E-03	-0.5242799
Thbd	thrombomodulin	1.00E-03	-0.6689993
Tmem100	transmembrane protein 100	4.32E-04	-0.4182826

Supplemental Table 4. The top 10 genes enriched in *WT* FOXF1^{+} gCAPs compared to *WT* FOXF1^{-} gCAPs, Wilcoxon Rank Sum test implemented via the function FindMarkers in Seurat package was used for data analysis.

Genes	Annotation	avg_logFC	p_val_adj
Foxf1	Forkhead Box F1	1.2614674	1.78E-09
Mki67	marker of proliferation Ki-67	2.15379841	2.55E-13
Cxcl12	C-X-C motif chemokine ligand 12	1.90661208	7.13E-09
Kit	KIT proto-oncogene	1.38418802	4.19E-12
Sparcl1	SPARC like 1	1.21722705	8.21E-14
Clec1a	C-type lectin domain family 1 member A	1.14760479	1.83E-16
Gja4	gap junction protein alpha 4	1.03271656	1.53E-07
Lpl	lipoprotein lipase	0.99784264	1.72E-06
Atp13a3	ATPase 13A3	0.95074354	3.96E-10
Car2	carbonic anhydrase 2	1.1179714	1.18E-11
Guk1	guanylate kinase 1	1.00858445	4.62E-07

Supplemental Table 5. The top 10 genes enriched in *WT* FOXF1^{-} gCAPs compared to *WT* FOXF1^{+} gCAPs, Wilcoxon Rank Sum test implemented via the function FindMarkers in Seurat package was used for data analysis.

Genes	Annotation	avg_logFC	p_val_adj
Fbln5	fibulin 5	2.01204657	4.8562E-21
Adgrg6	adhesion G protein-coupled receptor G6	1.78673214	1.19E-15
Ltbp4	latent TGFbeta binding protein 4	1.76309316	2.6156E-22
Mmp2	matrix metallopeptidase 2	1.49081924	3.22E-18
Car8	carbonic anhydrase 8	1.42904566	2.042E-14
Ackr3	atypical chemokine receptor 3	1.34393242	1.0378E-19
Plac8	placenta associated 8	1.20215519	7.1083E-20
Eln	elastin	1.19761339	4.8213E-21
Fbln2	fibulin 2	1.17566814	4.7209E-05
Hhip	hedgehog interacting protein	1.16070174	2.5673E-12

Supplemental Table 6. Mortality rates in *Foxf1*$^{WT/S52F}$ and *WT* mice after BMP9 treatment.

Mice	Untreated (n=236)	Saline (n=21)		BMP9 (n=42)	
Genotype of mice	*Foxf*$^{WT/S52F}$	*Foxf1*$^{+/+}$(WT)	*Foxf1*$^{WT/S52F}$	*Foxf1*$^{+/+}$(WT)	*Foxf1*$^{WT/S52F}$
Number of mice at P3	236	12	9	25	17
Number of mice at P18	95	11	3	23	10
Mortality rate (P3-P18)	59.75%	8.33%	66.67%	8.00%	41.18%

Supplemental Table 7. Antibody list for immunostaining (IF), Western blot (WB) and Flow cytometry (FC).

Antibody	Dilution	Conjugation	Assay	Company	Catlog No.
Foxf1	1:250	N/A	IF	R&D	AF4798
GFP	1:400	N/A	IF	Thermofisher	A11122
ACVRL1	1:300	N/A	IF	R&D	AF770
Eng(CD105)	1:200	N/A	IF	Thermofisher	14-1051-82
pSmad1	1:250	N/A	IF	Sigma	AB3848
Erg	1:500	N/A	IF	Abcam	ab92513
Emcn	1:300	N/A	IF	Abcam	ab106100
Hopx	1:250	N/A	IF	SantaCruz	sc30216
Fibronectin	1:250	N/A	IF	R&D	NBP1-91258
ACVRL1	1:300	Biotin	FC	R&D	BAF770
Kit	1:100	Superbright 780	FC	Thermofisher	78-1171-82
CD31	1:100	eFluor450	FC	Thermofisher	48-0311-82
CD45	1:100	APC-eFluor780	FC	Thermofisher	47-0451-82
CD140a	1:100	PE-Cy7	FC	Thermofisher	25-1401-82
Eng(CD105)	1:100	APC	FC	Biolegend	120413
CD45	1:100	AF700	FC	Thermofisher	56-0451-82
CD31	1:100	BV605	FC	Biolegend	102427
CD326	1:100	Percp-cy5.5	FC	Biolegend	118220
Smad1	1:500	N/A	WB	CST	9743
pSmad1	1:500	N/A	WB	CST	9516
Smad2	1:500	N/A	WB	SantaCruz	sc-6032
pSmad2	1:500	N/A	WB	SantaCruz	sc-11769
Foxf1	1:600	N/A	WB	R&D	AF4798
Actin	1:1000	N/A	WB	SantaCruz	sc47778

Supplemental Table 8. The Taqman primers used for qRT-PCR analysis.

**Target genes**	**Taqman assay Cat. No.**
Pecam1	Mm01242584_m1
Foxf1	Mm00487497_m1
Acvrl1	Mm00437432_m1
Acvr1	Mm01331069_m1
Acvrl1b	Mm00475713_m1
Bmpr1a	Mm00477650_m1
Bmpr1b	Mm01312643_m1
Tgfbr1	Mm00436964_m1
Tmem100	Mm00471352_m1
Bmpr2	Mm00432134_m1
Eng	Mm00468252_m1
Nkx2-1	Mm00447558_m1
Kit	Mm00445212_m1
β-actin	Mm00607939_s1
Id3	Mm00492575_m1
Id1	Mm03676649_s1
Carcrl	Mm00516986_m1
Clec14a	Mm00482102_s1

Supplemental Table 9. Primers used to construct the luciferase reporter plasmids.

**Primer name**	**Oilgo sequence**
Acvrl1-400~F'	ACTGGTACCAGAGCTGTGTAAGGTACCTACACAAACGTC
Acvrl1-400~R'	TCAGCTAGCCACTGCAACTGTTCAGAGGGTAATAGGCCG
Foxf1-350~F'	TCCGGTACCCGGCCTGGCGCGCAGCGTCGGAGGCC
Foxf1-350~R'	CAGGCTAGCACCAGTGCGCTCCCCACTCACGTTCC
Acvrl1~80-F'	CAATCTAAACAATCTTGATTCCTGTTGCCGGCCTGGCGGGACCCTGAATGGCAGGAAGTAAGGACAAGAGCCTGTTTATG
Acvrl1~80-R'	CTAGCATAAACAGGCTCTTGTCCTTACTTCCTGCCATTCAGGGTCCCGCCAGGCCGGCAACAGGAATCAAGATTGTTTAGATTGGTAC
Acvrl1mt~80-F'	CAATCTCCCCAATCTTGATTCCTGTTGCCGGCCTGGCGGGACCCTGAATGGCAGGAAGTAAGGACAAGAGCCTGGGGATG
Acvrl1mt~80-R'	CTAGCATCCCCAGGCTCTTGTCCTTACTTCCTGCCATTCAGGGTCCCGCCAGGCCGGCAACAGGAATCAAGATTGGGGAGATTGGTAC

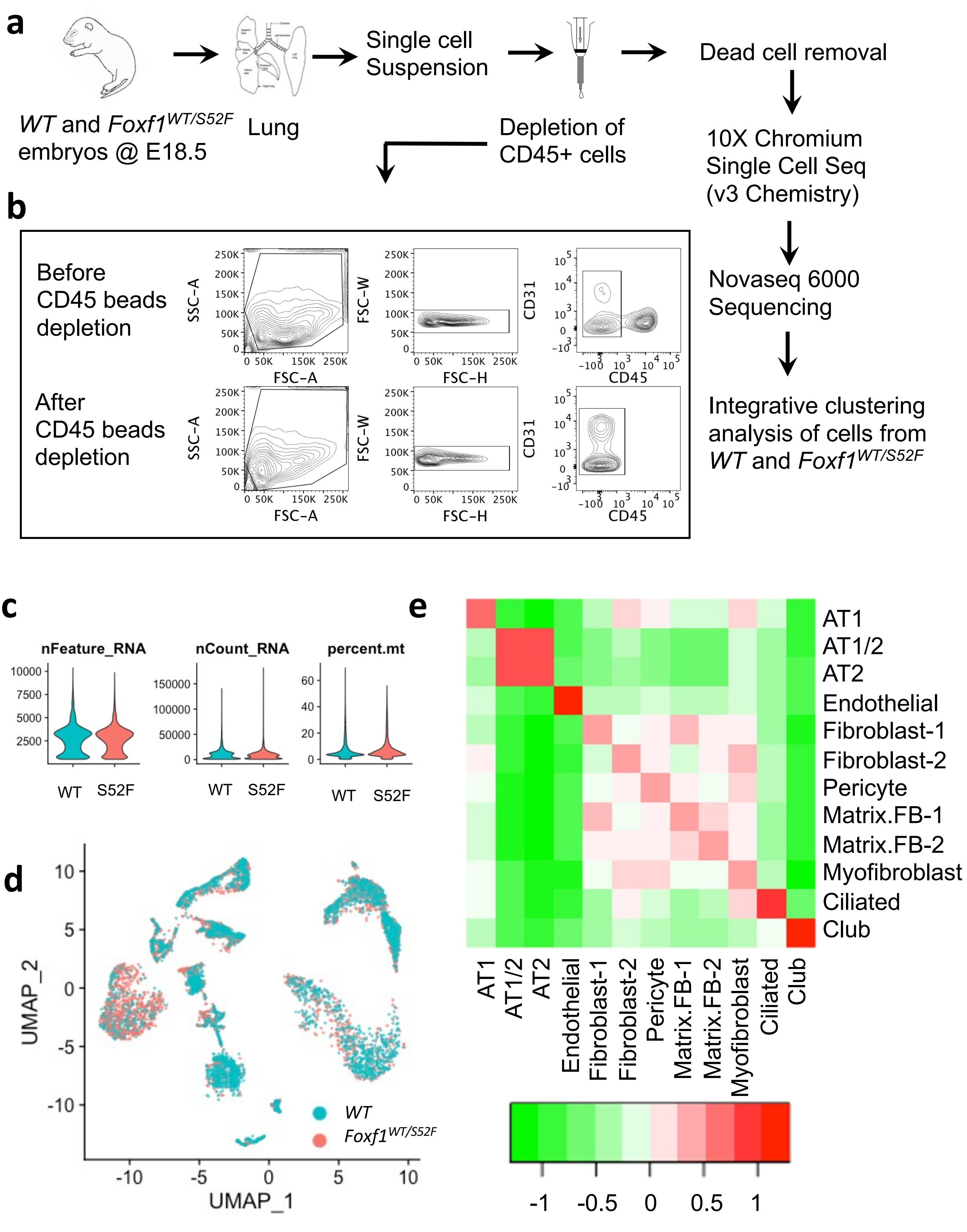
a
WT and Foxf1WT/S52F embryos @ E18.5
Lung
Single cell Suspension
Depletion of CD45+ cells
Dead cell removal
10X Chromium Single Cell Seq (v3 Chemistry)
Novaseq 6000 Sequencing
Integrative clustering analysis of cells from WT and Foxf1WT/S52F
b
Before CD45 beads depletion
After CD45 beads depletion
SSC-A
FSC-A
FSC-W
FSC-H
CD31
CD45
c
nFeature_RNA
nCount_RNA
percent.mt
WT
S52F
d
UMAP_1
UMAP_2
WT
Foxf1WT/S52F
e
AT1
AT1/2
AT2
Endothelial
Fibroblast-1
Fibroblast-2
Pericyte
Matrix.FB-1
Matrix.FB-2
Myofibroblast
Ciliated
Club
-1
-0.5
0
0.5
1

Supplemental Figure 1. Integrated clustering of CD45^{-} cells isolated from *WT* and *Foxf1$^{WT/S52F}$* lungs. *a,* Schematic shows the single cell preparation from WT and *Foxf1$^{WT/S52F}$* E18.5 lungs followed by the library preparation and scRNAseq. *b,* FACS analysis shows the depletion of CD45^{+} hematopoietic cells from lung cell suspensions. Depletion of hematopoietic cells was carried out using CD45-specific immunobeads. *c,* The comparison of raw data quality from scRNAseq analysis of *WT* and *Foxf1$^{WT/S52F}$* E18.5 lungs. *d,* UMAP plot shows CD45^{-} cell clusters in *WT* and *Foxf1$^{WT/S52F}$* lungs. *e,* Correlation plot shows the integrated clustering of 12 pulmonary cell types, which was generated using a correlative matrix. Correlative matrix was produced from the average expression for all features across the clusters.

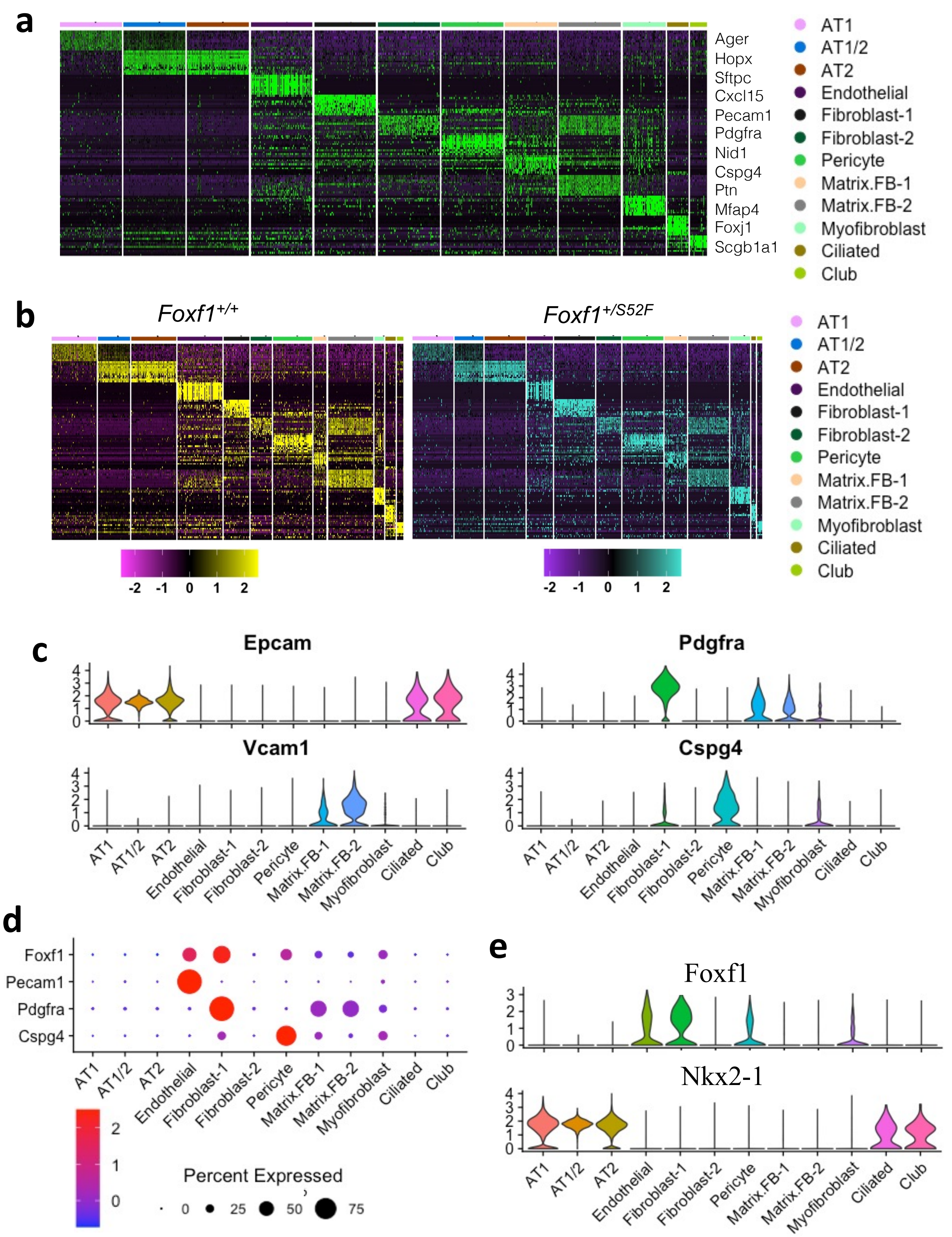
a
Ager
Hopx
Sftpc
Cxcl15
Pecam1
Pdgfra
Nid1
Cspg4
Ptn
Mfap4
Foxj1
Scgb1a1
AT1
AT1/2
AT2
Endothelial
Fibroblast-1
Fibroblast-2
Pericyte
Matrix.FB-1
Matrix.FB-2
Myofibroblast
Ciliated
Club
b
Foxf1+/+
Foxf1+/S52F
-2 -1 0 1 2
c
Epcam
Pdgfra
Vcam1
Cspg4
d
Foxf1
Pecam1
Pdgfra
Cspg4
Percent Expressed
0 25 50 75
e
Foxf1
Nkx2-1

Supplemental Figure 2. Comparison of gene expression signatures between *WT* and *Foxf1*$^{WT/S52F}$ lungs. *a,* Integrated heatmap shows expression of 20 marker genes specific for each cell cluster. Cells from *WT* and *Foxf1*$^{WT/S52F}$ E18.5 lungs were combined together to generate the integrated heatmap. *b,* Parallel heatmaps show expression of 20 marker genes in each cell cluster from *WT* and *Foxf1*$^{WT/S52F}$ E18.5 lungs. *c,* Violin plots show expression of specific mRNAs for epithelial cells (*Epcam*), fibroblasts (*Pdgrfa*), matrix fibroblasts (*Vcam1*) and pericytes (*Cspg4*). Violin plots were generated using a combined pool of cells from *WT* and *Foxf1*$^{WT/S52F}$ lungs. *d-e,* scRNAseq data demonstrate that *Foxf1* mRNA is enriched in clusters of endothelial cells, fibroblast-1, pericytes and myofibroblasts. *Foxf1* is not detected in endoderm-derived epithelial cell lineages, including AT1, AT2, AT1/2, Club cells and Ciliated cells. Epithelial cell clusters are marked by *Nkx2-1* mRNA.

Wang et al, Supplemental Fig. 3

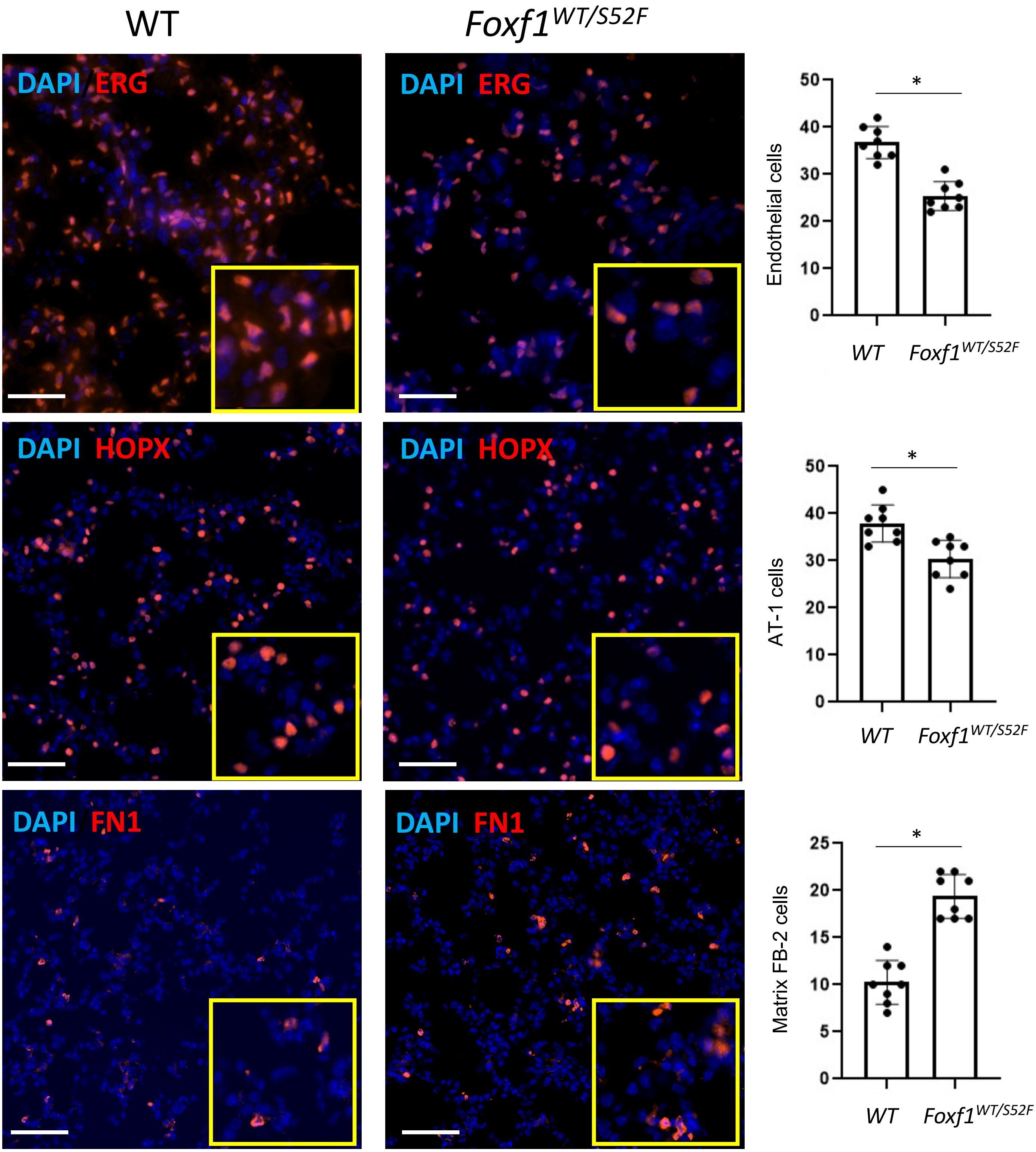

Supplemental Figure 3. *Foxf1$^{WT/S52F}$* lungs exhibit decreased numbers of endothelial and AT1 cells in the alveolar region. Immunostaining of *WT* and *Foxf1$^{WT/S52F}$* E18.5 lungs was performed to identify endothelial cells (ERG antibody), AT1 cells (HOPX antibody) and Matrix. FB-2 cells (FN1 antibody). Lung sections were counterstained with DAPI. Compared to lungs of WT littermates, the percentages of endothelial and AT1 cells are decreased in *Foxf1$^{WT/S52F}$* lungs, whereas the percentage of Matrix. FB-2 cells is increased (n=8 per group), Data were presented as mean ± SD, and T-test (two-tailed) analyses were performed with GraphPad Prism, P values are 0.010006 (Endothelial),0.018039 (AT1),0.01165 (Matrix.FB-2). $p<0.05$ is *, $p<0.01$ is **. Scale bars are 20µm. Source data are provided as a Source Data file.

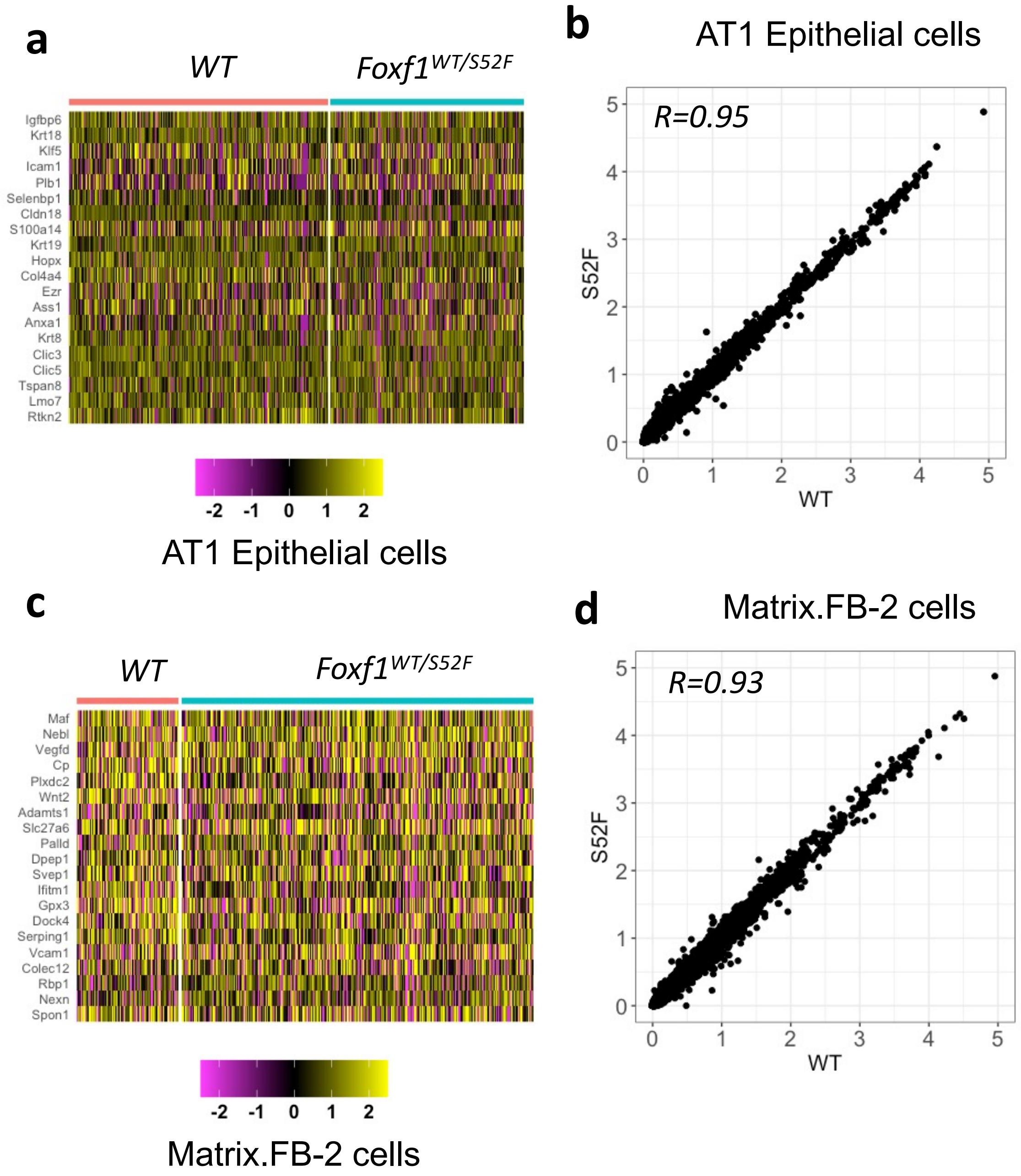

Supplemental Figure 4. Gene signatures of AT1 and Matrix Fibroblasts 2 are similar between *WT* and *Foxf1*$^{WT/S52F}$ lungs. Parallel heatmaps and correlation plots show that gene signatures of AT1 cells (*a*-*b*) and matrix fibroblasts 2 (Matrix.FB-2) (*c*-*d*) are similar between *WT* and *Foxf1*$^{WT/S52F}$ E18.5 lungs.

Wang et al, Supplemental Fig. 5

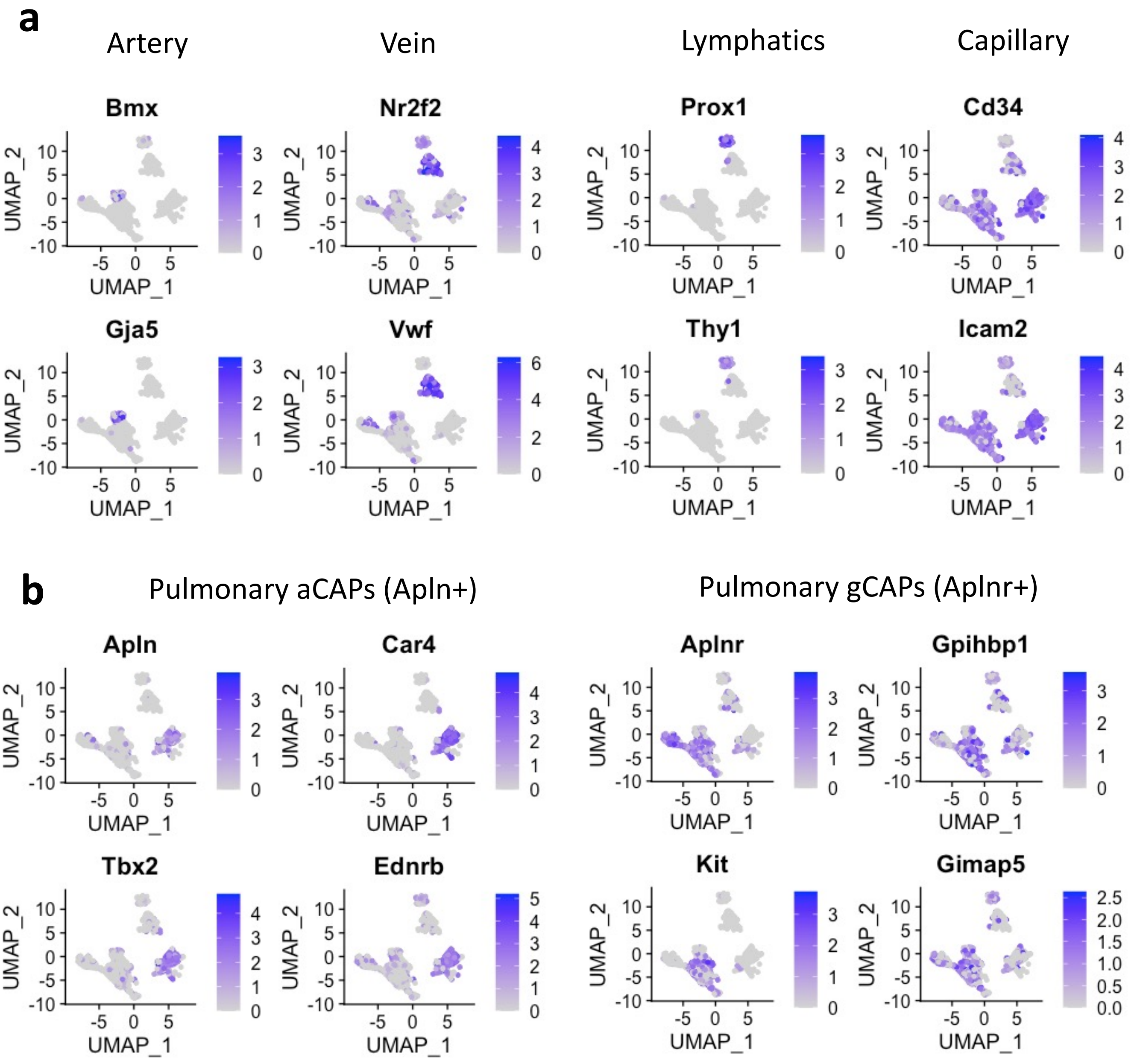

Supplemental Figure 5. Endothelial marker genes identify clusters of arterial, venous, lymphatic and capillary endothelial cells in the mouse lung. *a,* Plots show expression of marker genes that identify distinct endothelial cell clusters in the lung tissue. Cells from *WT* and *Foxf1*$^{WT/S52F}$ E18.5 lungs were combined together prior to UMAP clustering. Arterial endothelial cluster is identified by *Bmx* and *Gja5*. Veinous endothelium is enriched in *Vwf* and *Nr2f2* mRNAs, whereas lymphatic endothelial cells express *Prox1* and *Thy1*. Capillary endothelial cells are enriched in *Icam2* and *Cd34* mRNAs. *b,* Plots show expression of *Car4, Tbx2, Apln* and *Ednrb* in aCAPs (aerocytes). gCAPs (general capillary cells) express *Aplnr*, *Gpihbp1*, *Kit* and *Gimap5*.

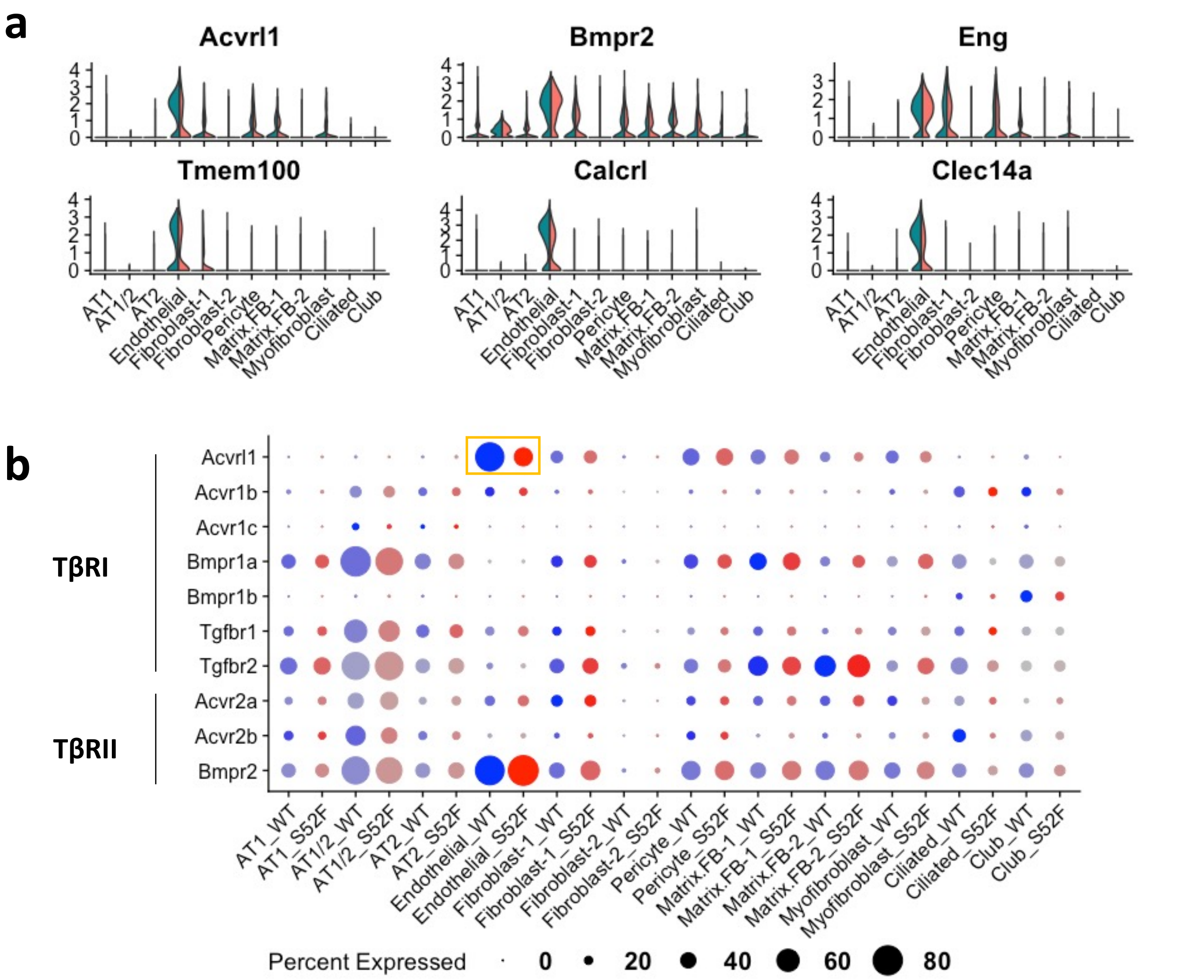

Supplemental Figure 6. *Acvrl1* mRNA is enriched in pulmonary endothelial cells. *a,* Violin plots show an enrichment of *Acvrl1* and its downstream target genes (*Tmem100, Calcrl* and *Clec14a*) in endothelial cell cluster. Expression of *Acvrl1, Tmem100, Calcrl* and *Clec14a* is decreased in endothelial cells from *Foxf1*$^{WT/S52F}$ E18.5 lungs compared to *WT* controls. *Bmpr2* and *Eng* mRNAs are also present in endothelial cells, but their expression is not changed in *Foxf1*$^{WT/S52F}$ lungs. *b,* Expression levels of genes encoding Type I (TβRI) and Type II receptors (TβRII) of the TGFβ signaling pathway in pulmonary cell types. *Acvrl1* mRNA is enriched in endothelial cells. Endothelial *Acvrl1* expression is decreased in *Foxf1*$^{WT/S52F}$ lungs compared to *WT* controls.

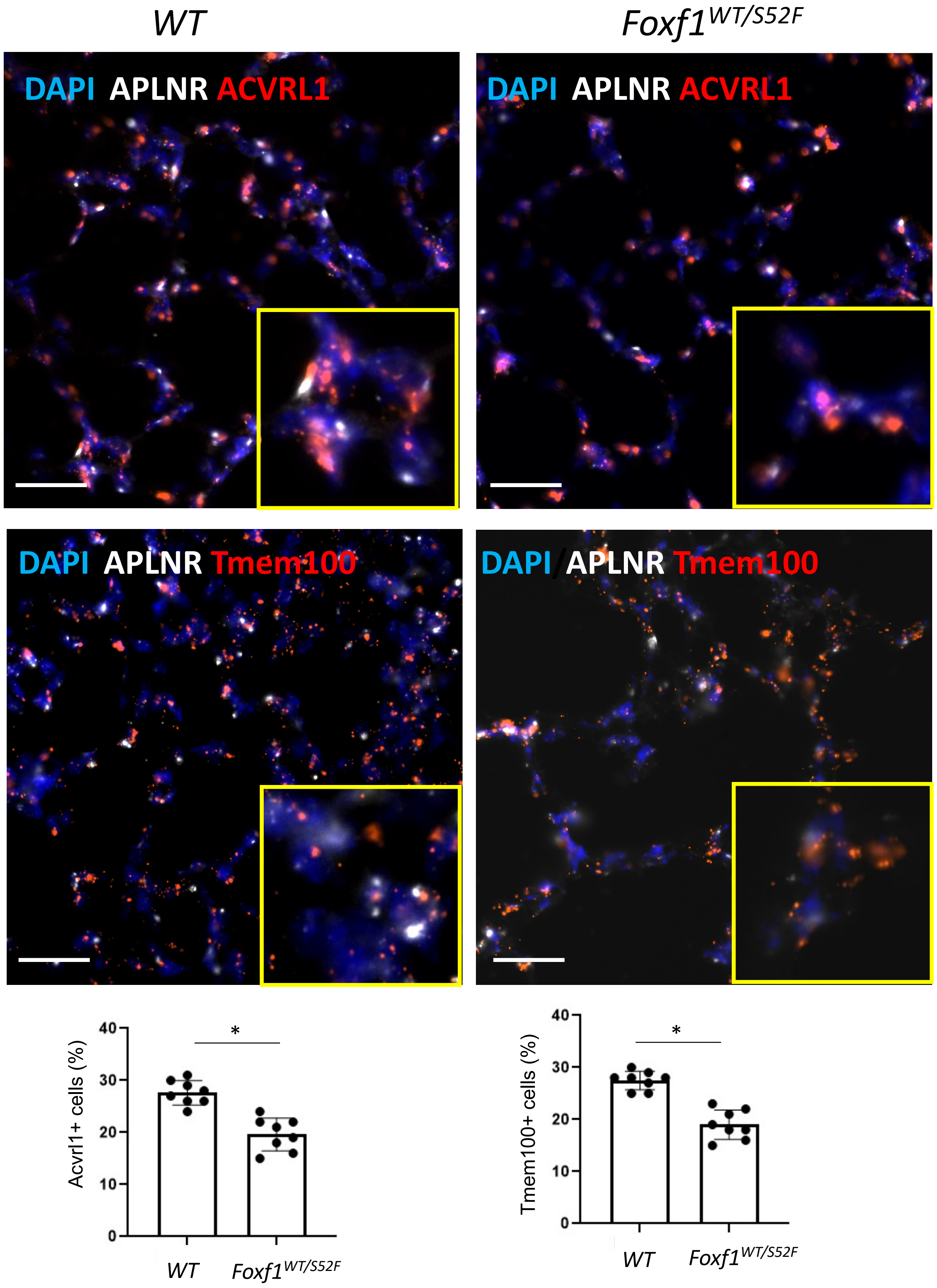
WT
Foxf1 WT/S52F
DAPI APLNR ACVRL1
DAPI APLNR ACVRL1
DAPI APLNR Tmem100
DAPI/APLNR Tmem100
Acvrl1+ cells (%)
40
30
20
10
0
*
WT
Foxf1 WT/S52F
Tmem100+ cells (%)
40
30
20
10
0
*
WT
Foxf1 WT/S52F

Supplemental Figure 7. *Foxf1$^{WT/S52F}$* lungs exhibit decreased numbers of cells expressing ACVRL1 and Tmem100.

RNAscope of *WT* and *Foxf1$^{WT/S52F}$* E18.5 lungs was performed using riboprobes specific *Aplnr*, *Acvrl1* and *Tmem100*. Lung sections were counterstained with DAPI. Compared to lungs of WT littermates, the percentages of cells expressing *Acvrl1* and *Tmem100* are decreased in *Foxf1$^{WT/S52F}$* lungs (n=8 per group), Data were presented as mean ± SD, and student's T-test (two-tailed) analyses were performed, p values are 0.018707 (Acvrl1), 0.01490 (Tmem100), $p<0.05$ is *, $p<0.01$ is **. Scale bars are 20µm.

Wang et al, Supplemental Fig. 8

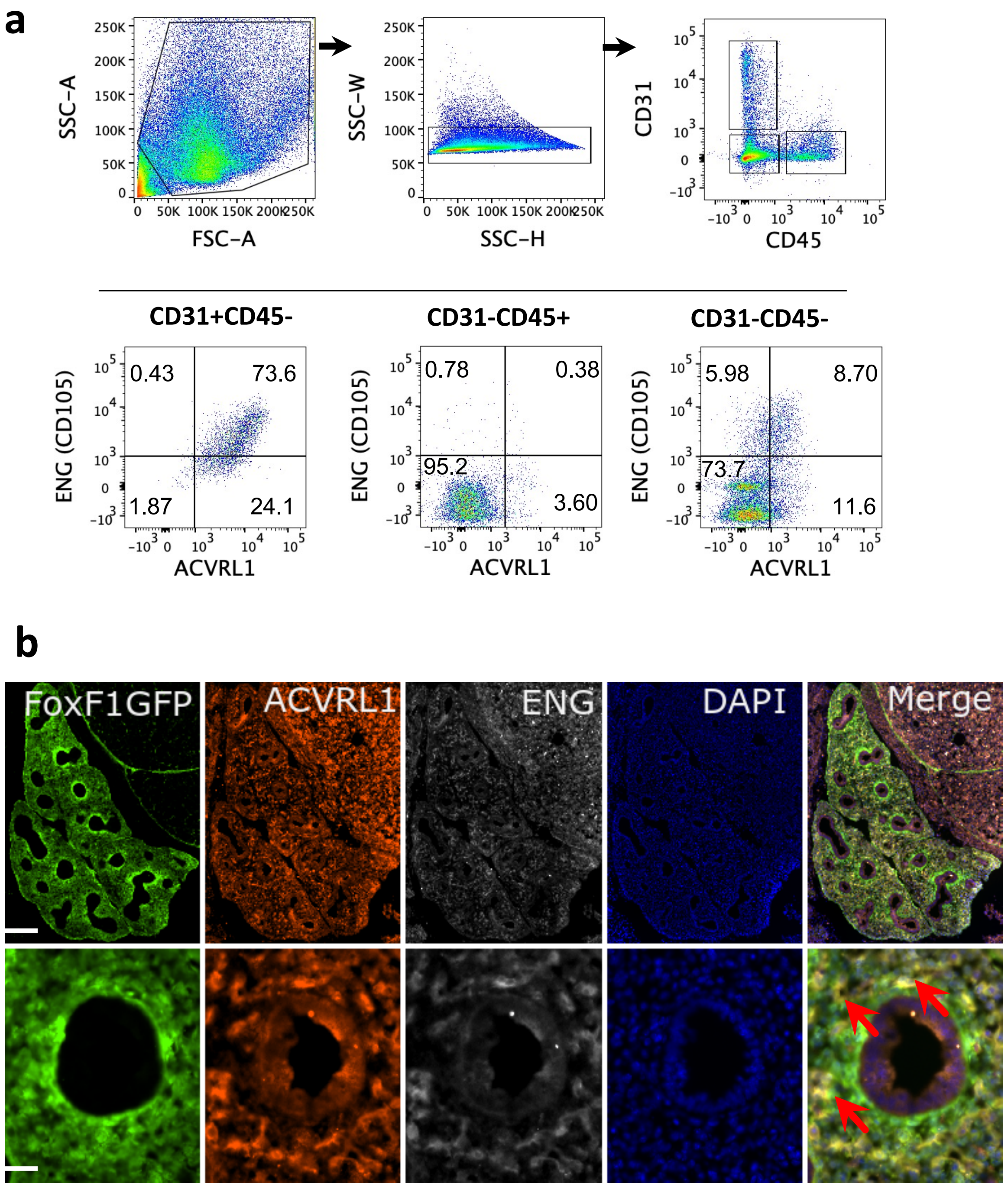

Supplemental Figure 8. ACVRL1 and ENG receptors are co-expressed in FOXF1$^+$ endothelial cells in the mouse embryonic lung. *a,* FACS analysis shows that both ACVRL1 and ENG proteins are detected in CD31$^+$CD45$^-$ pulmonary endothelial cells but not in CD45$^+$CD31$^-$ hematopoietic cells. *b,* Immunostaining shows that *Foxf1-GFP* reporter co-localizes with ACVRL1 and ENG (arrows) in lungs of *Foxf1*$^{WT\text{-}GFP/+}$ E15.5 mouse embryos. DAPI was used to counterstain cell nuclei. The immunofluorescence staining was independently repeated twice with consistent results. Scale bars are 50μm (top images) and 10μm (bottom images).

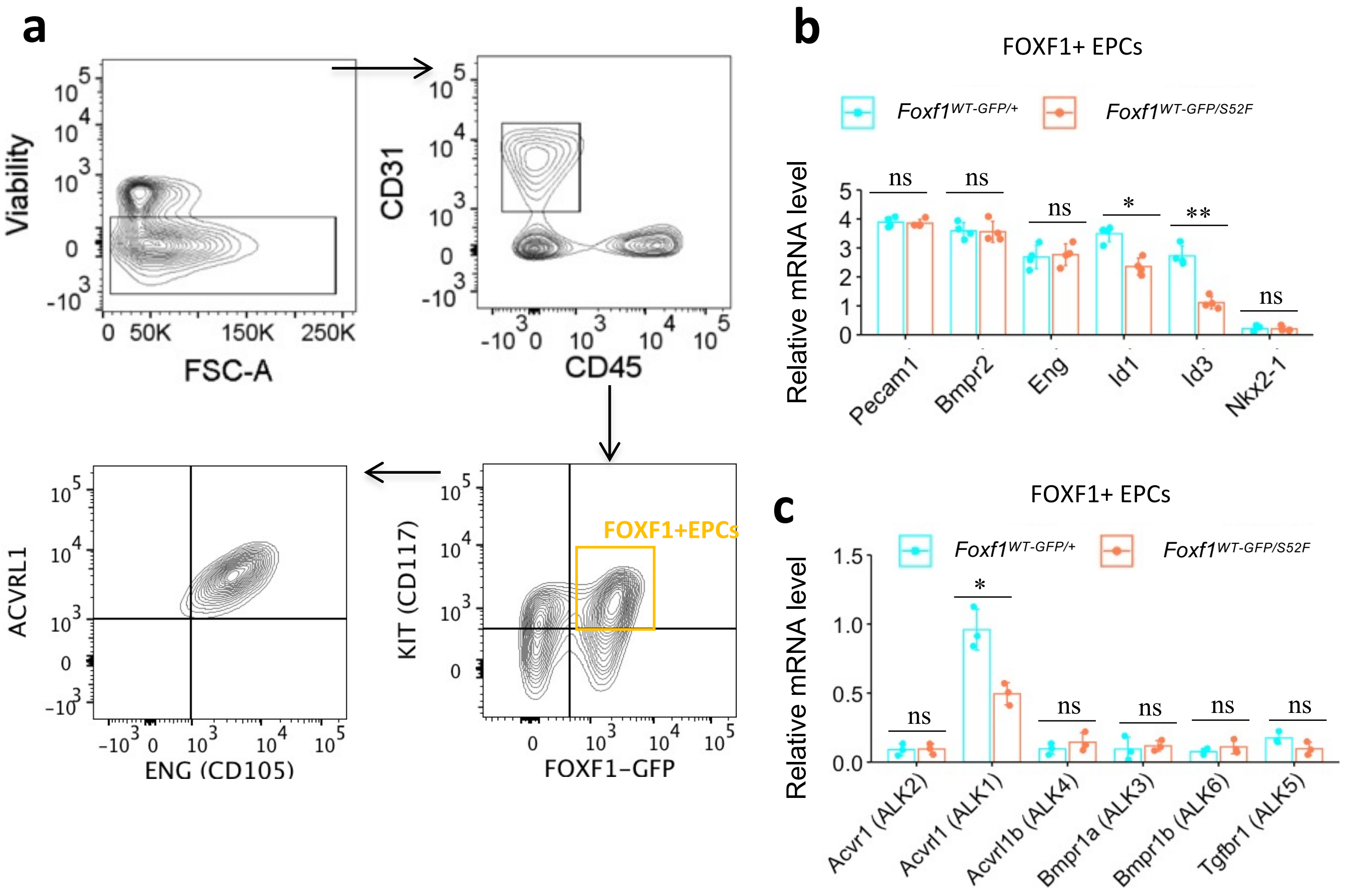

Supplemental Figure 9. Expression of *Id1*, *Id3* and *Acvrl1* is decreased in FOXF1⁺ EPCs isolated from *Foxf1*$^{WT\text{-}GFP/S52F}$ lungs. *a,* FACS gating strategy shows the isolation of FOXF1^{+} gCAPs (FOXF1^{+}cKIT^{+}CD31^{+}CD45^{-} cells; FOXF1^{+} EPCs) from lungs of *Foxf1*$^{WT\text{-}GFP/+}$ reporter mice. FACS-sorted FOXF1^{+} EPCs express ACVRL1 and ENG receptors on the cell surface. *b-c,* qRT-PCR analysis shows decreased *Id1*, *Id3* and *Acvrl1* mRNAs in FACS-sorted FOXF1^{+} EPCs from *Foxf1*$^{WT\text{-}GFP/S52F}$ lungs compared to *Foxf1*$^{WT\text{-}GFP/+}$ controls (n=4). Expression levels were normalized using *β-Actin* mRNA, Data were shown as mean ± SD, and nonparametric Mann Whitney U test were performed were performed, and p value for Pecam1 is 0.77186, Bmpr2 is 0.88336, Eng is 0.78076, Id1 is 0.01341, Id3, 0.00197, Nkx2-1 is 0.99243, Acvrl1 (ALK1) is 0.00892, Acvr1 (ALK2) is 0.88627,Bmpr1a (ALK3) is 0.70214, Acvrl1b (ALK4) is 0.35451, Tgfbr1 (ALK5) is 0.10324, Bmpr1b (ALK6) 0.32937. $p<0.05$ is *, $p<0.01$ is **, ns is not significant.

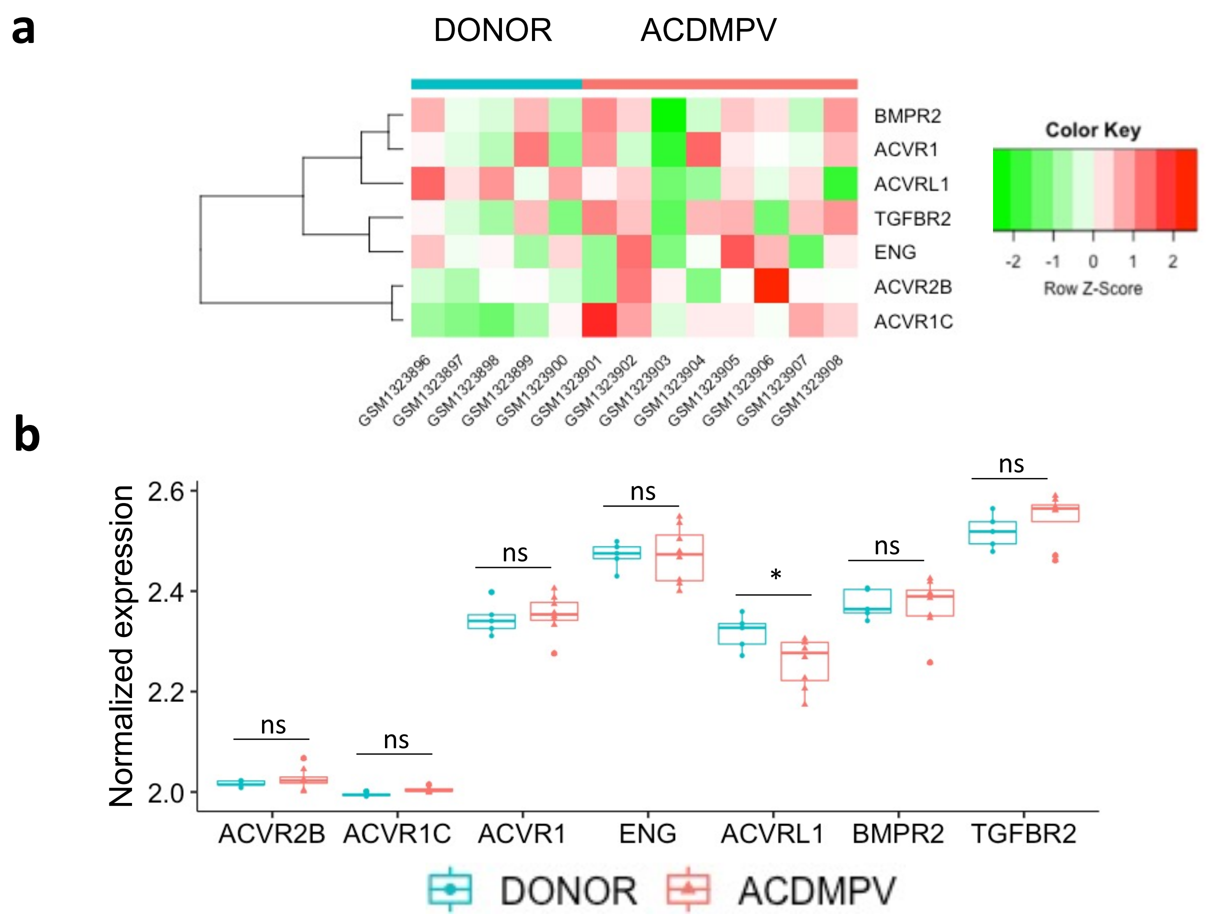

Supplemental Figure 10. *ACVRL1* mRNA is selectively decreased in human ACDMPV lungs. Microarray analysis shows expression of mRNA transcripts encoding the Type I and Type II TGFβ/BMP receptors in ACDMPV (n=8) and donor lungs (n=5). Microarray data are displayed as a heatmap (*a*) and a boxplot (*b*). *ACVRL1* mRNA is selectively decreased in ACDMPV lungs. mRNAs encoding other TGFβ/BMP receptor genes are unchanged, Boxplots: center line, median; box boundary, first and third quartiles; whiskers denote 5th–95th percentile, and student's T-test (two-tailed) were performed. $p<0.05$ is *, $p<0.01$ is **, ns is not significant.

Wang et al, Supplemental Fig. 11

a

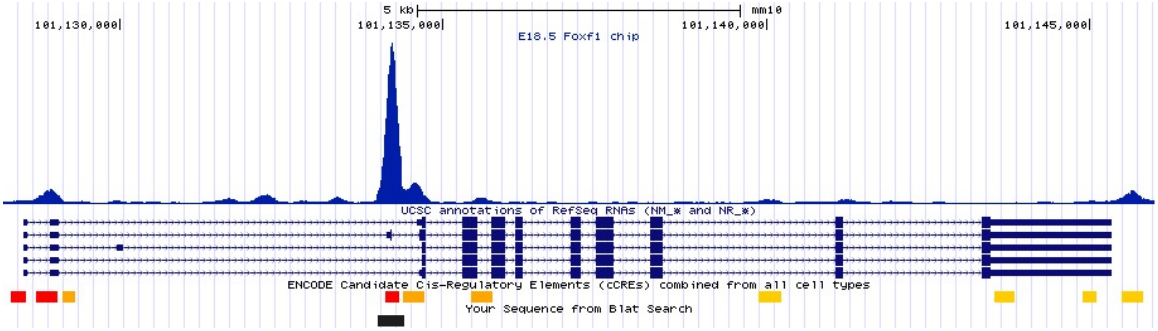

b

ACE400

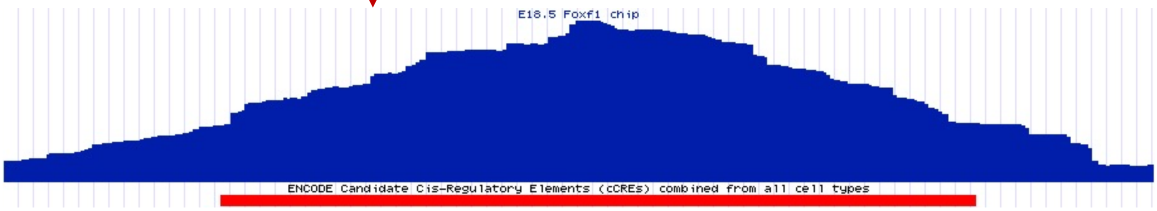

-675bp -275bp

c

CAGAGCTGTGTAAGGTACCTACACAAACGTCACACAGTTG
TCTCTGCTCCTGGTGGTCCCTTTAAACCCCCGTCCCGCTT
CCAGCCTGCCCTGAGCTCCCTGCTGTGCGTGACTTCAGCC
*TGTTCTATCCA***GGCCTCAATCTAAACAATCTTGATTCCTG**
TTGCCGGCCTGGCGGACCCTGAATGGCAGGA*AGTAAGGA*
CAAGAGCCTGTTTATGTTTGAAGCAGCCAGGCTGGGGGTG
GGGAGTGGGGGCACTGGGAAACGGTGGGCAGGGGTGGAGG
CTGGAGCGATGGGCAAACGGCTGAGGACAAGAGATGAGCT
ATGAGAGAGTCGTCCTTCCCACTGTAGCTCTGTCTGTCTG
CAACCCTCCCGGCCTATTACCCTCTGAACAGTTGCAGTGG

Supplemental Figure 11. FOXF1 protein directly binds to ACE400 region in mouse *Acvrl1* promoter. *a,* ChIPseq analysis shows FOXF1-binding regions in mouse *Acvrl1* gene. ChIPseq was performed using lung tissue from mouse E18.5 embryos. The black bar indicates ACE400 DNA region. Based on *ENCODE Registry of candidate cis-Regulatory Elements* (cCREs) of the mouse genome, red bars show promoter-like signatures (PLS), whereas yellow bars indicate enhancer-like signatures (pELS). *b,* The detailed view of ACE400 DNA region in UCSC genome browser. The red bar indicates EM10E0612669 cCRE, which derives from the ChIPseq signal of histone methylation markers in P0 mouse lung. *c,* The ACE400 DNA sequence is obtained from *mm10* genome (chr15:101,134,005-101,134,404). Potential FOXF1-binding sites are indicated with green. Bold letters indicate promoter sequences for NM_001277255 transcript.

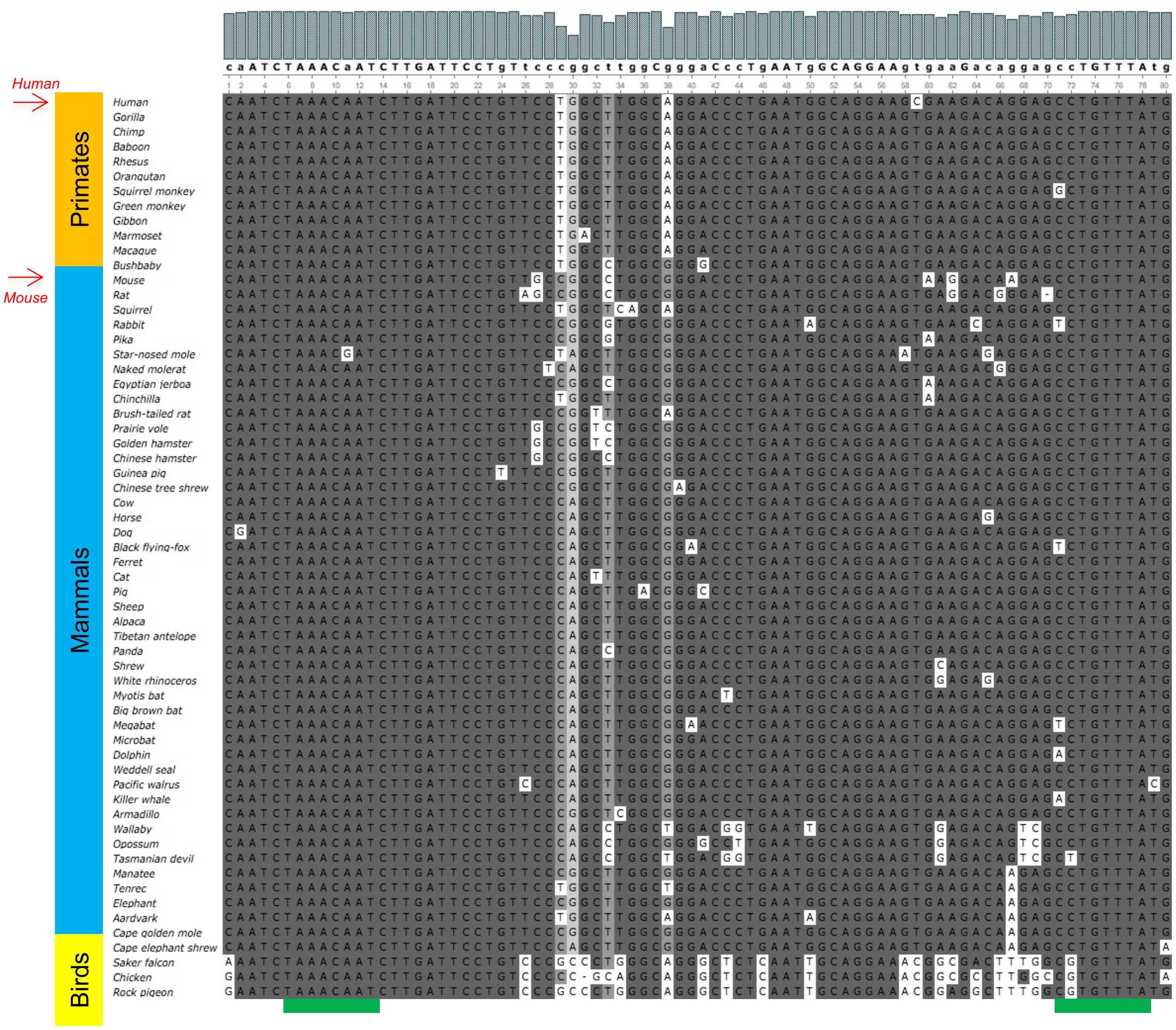

Supplemental Figure 12. ACE80 DNA region is conserved in mammals and birds. DNA sequences show the ACE80 region in 60 vertebrate genomes obtained from UCSC genome browser. Sequences are aligned using the ClustalW2 algorithm. Green bars show two evolutionarily conserved FOXF1-binding sites. Vertebrate genomes are classified into Primates, Birds and other mammals (*Laurasiatheria, Euarchontoglires* and *Afrotheria*).

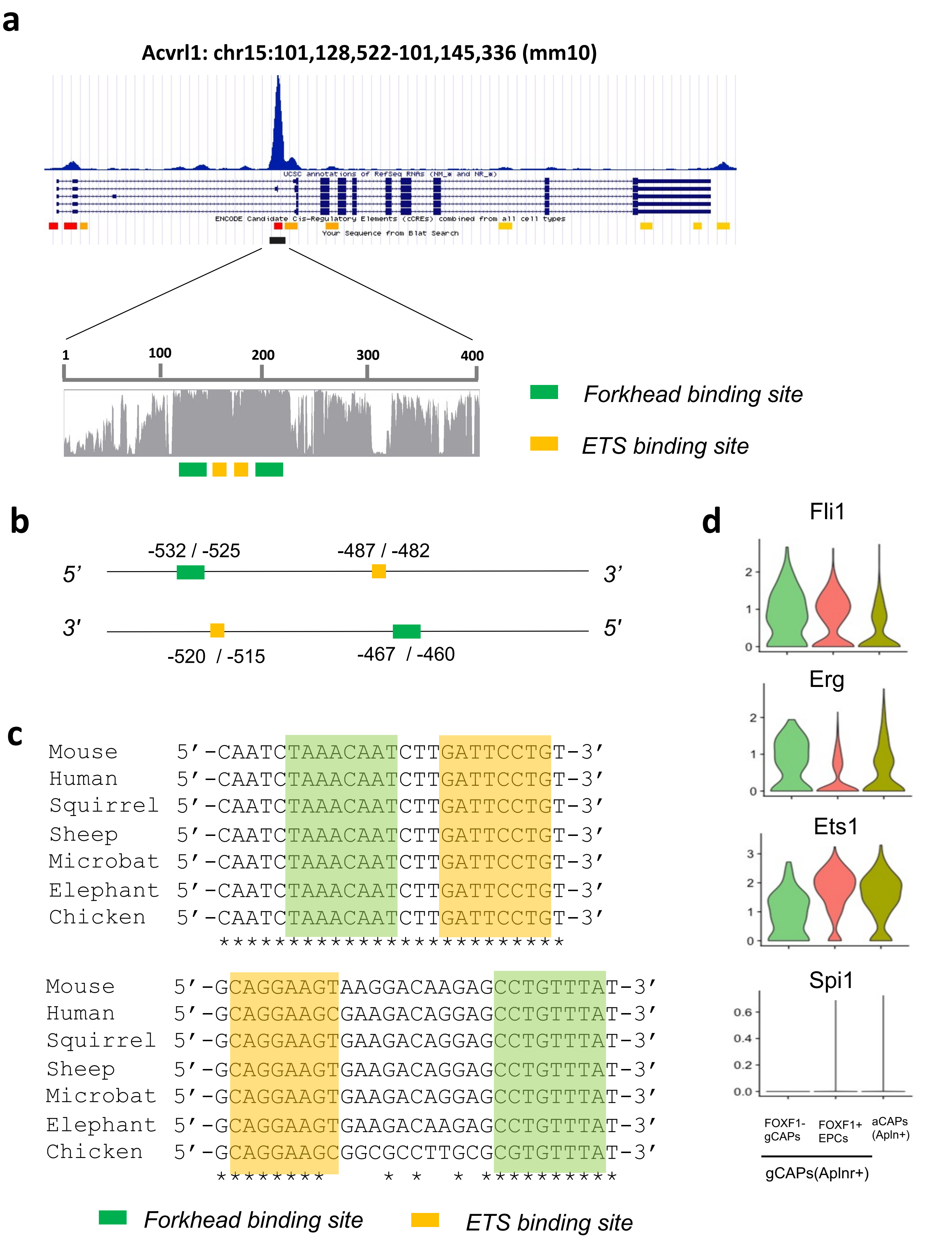
a
Acvrl1: chr15:101,128,522-101,145,336 (mm10)
UCSC annotations of RefSeq RNAs (NM_* and NR_*)
ENCODE Candidate Cis-Regulatory Elements (cCREs) combined from all cell types
Your Sequence from Blat Search
1
100
200
300
400
Forkhead binding site
ETS binding site
b
-532 / -525
-487 / -482
5'
3'
3'
5'
-520 / -515
-467 / -460
c
Mouse 5'-CAATCTAAACAATCTTGATTCCTGT-3'
Human 5'-CAATCTAAACAATCTTGATTCCTGT-3'
Squirrel 5'-CAATCTAAACAATCTTGATTCCTGT-3'
Sheep 5'-CAATCTAAACAATCTTGATTCCTGT-3'
Microbat 5'-CAATCTAAACAATCTTGATTCCTGT-3'
Elephant 5'-CAATCTAAACAATCTTGATTCCTGT-3'
Chicken 5'-CAATCTAAACAATCTTGATTCCTGT-3'
Mouse 5'-GCAGGAAGTAAGGACAAGAGCCTGTTTAT-3'
Human 5'-GCAGGAAGCGAAGACAGGAGCCTGTTTAT-3'
Squirrel 5'-GCAGGAAGTGAAGACAGGAGCCTGTTTAT-3'
Sheep 5'-GCAGGAAGTGAAGACAGGAGCCTGTTTAT-3'
Microbat 5'-GCAGGAAGTGAAGACAGGAGCCTGTTTAT-3'
Elephant 5'-GCAGGAAGTGAAGACAAGAGCCTGTTTAT-3'
Chicken 5'-GCAGGAAGCGGCGCCTTGCGCGTGTTTAT-3'
Forkhead binding site
ETS binding site
d
Fli1
Erg
Ets1
Spi1
FOXF1- gCAPs
FOXF1+ EPCs
aCAPs (Apln+)
gCAPs(Aplnr+)

Supplemental Figure 13. Conserved FOX and ETS binding motifs are present in ACE400 region of *Acvrl1* gene. *a,* ChIPseq analysis of mouse E18.5 lungs shows FOXF1-binding regions in mouse *Acvrl1* gene. The black bar indicates ACE400 DNA region with 2 *Forkhead* (FOX) and 2 ETS binding motifs. Based on *ENCODE Registry of candidate cis-Regulatory Elements* (cCREs) of the mouse genome, red bars show promoter-like signatures (PLS), whereas yellow bars indicate enhancer-like signatures (pELS). *b,* Schematic shows the position of FOX (green) and ETS binding motifs (yellow) in ACE400 DNA. *c,* UCSC genome browser show that FOX and ETS binding motifs in ACE400 are conserved. *d,* Violin plots show expression profiles of ETS transcription factors *Fli1, Erg, Ets1* and *Spi1* in aCAPs, $FOXF1^{-}$ gCAPs and $FOXF1^{+}$ gCAPs ($FOXF1^{+}$ EPCs). scRNAseq analysis was performed using *WT* E18.5 lungs.

Wang et al, Supplemental Fig. 14

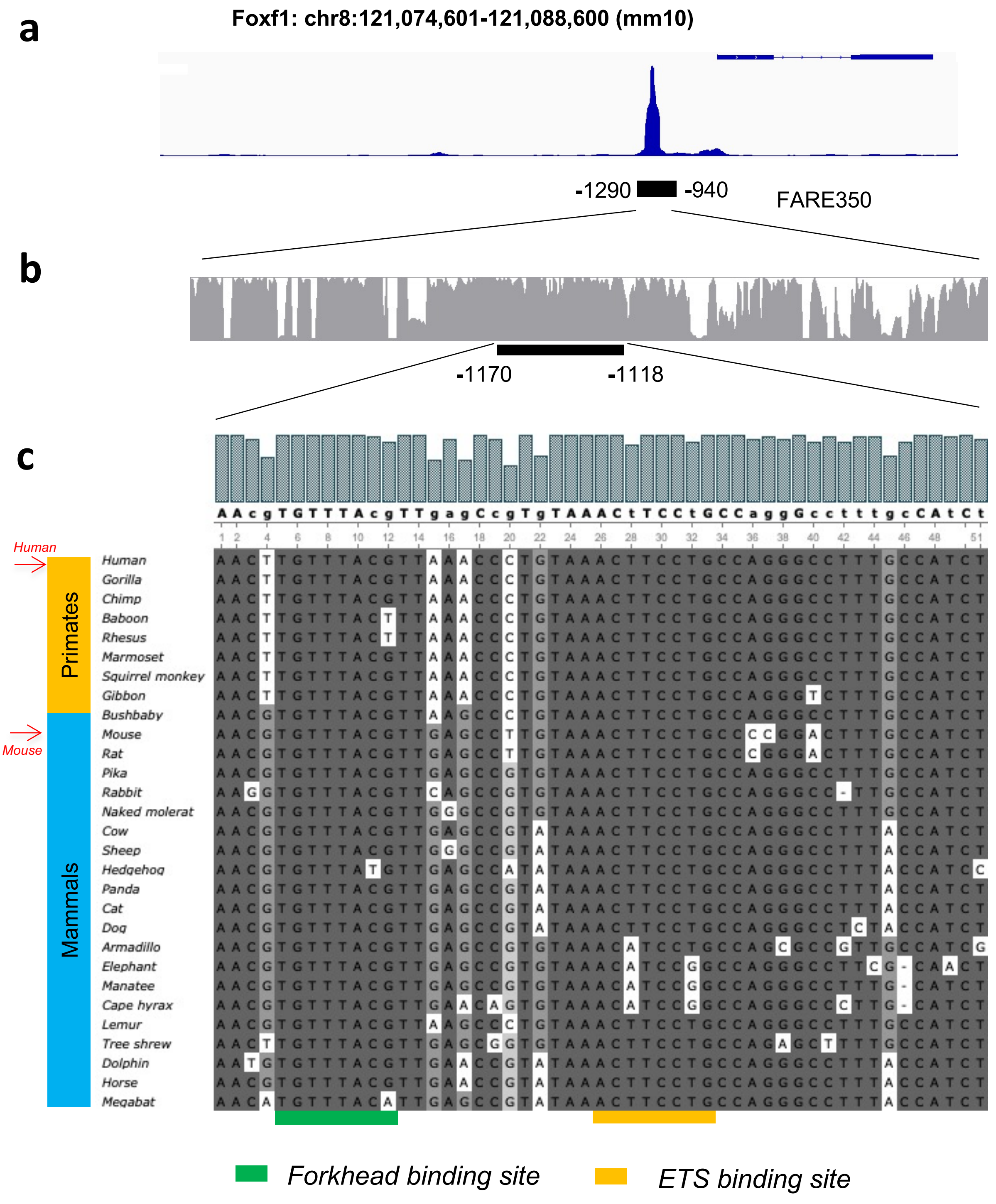

Supplemental Figure 14. Conserved FOX and ETS binding motifs are present in FARE350 region of *Foxf1* gene. *a-b,* ChIPseq analysis shows FOXF1-binding regions in FARE350 region of mouse *Foxf1* gene. *c,* The alignment of DNA sequences shows that FARE350 region is conserved in vertebrate genomes obtained from UCSC genome browser. Sequences are aligned using the ClustalW2 algorithm. Evolutionarily conserved FOX (green) and ETS binding motifs (yellow) are present in FARE350.

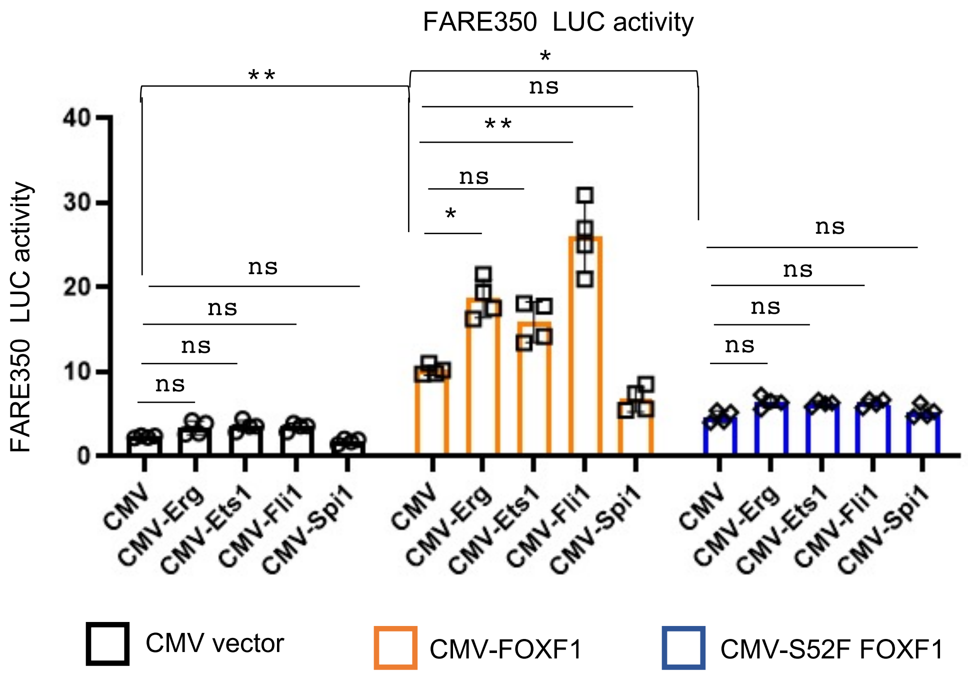

Supplemental Figure 15. FOXF1 synergizes with FLI1 to stimulate *Foxf1* promoter activity. Dual luciferase (LUC) assay shows transcriptional synergy between CMV-FOXF1 and CMV-FLI1 expression vectors to activate the FARE350 LUC reporter. Data were presented as mean ± SD, and one-way ANOVA followed by Tukey's test (two-tailed) were performed. Co-transfection experiments were performed using fetal lung MFLM-91U cells (n=4), p<0.01 is **, p<0.05 is *, ns is not significant.

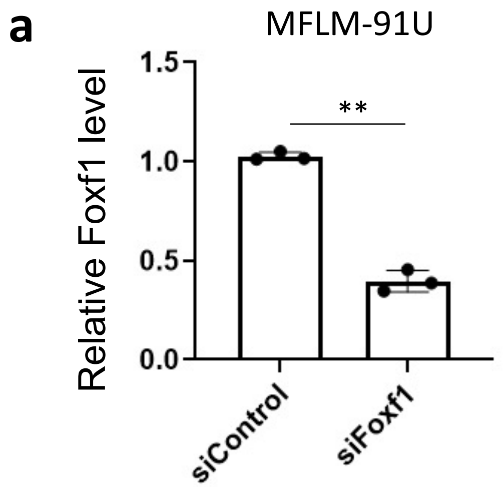
a
MFLM-91U
Relative Foxf1 level
1.5
1.0
0.5
0.0
**
siControl
siFoxf1

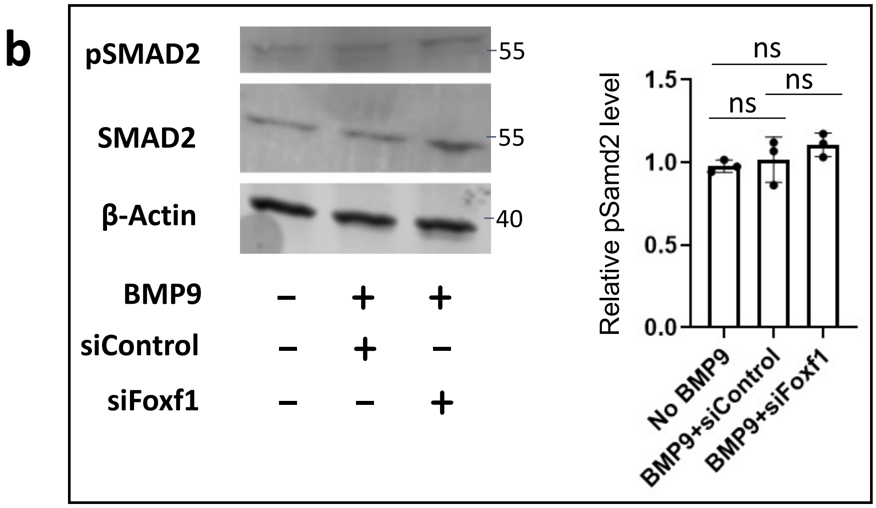
b
pSMAD2
SMAD2
β-Actin
55
55
40
BMP9 − + +
siControl − + −
siFoxf1 − − +
Relative pSamd2 level
1.5
1.0
0.5
0.0
ns
ns
ns
No BMP9
BMP9+siControl
BMP9+siFoxf1

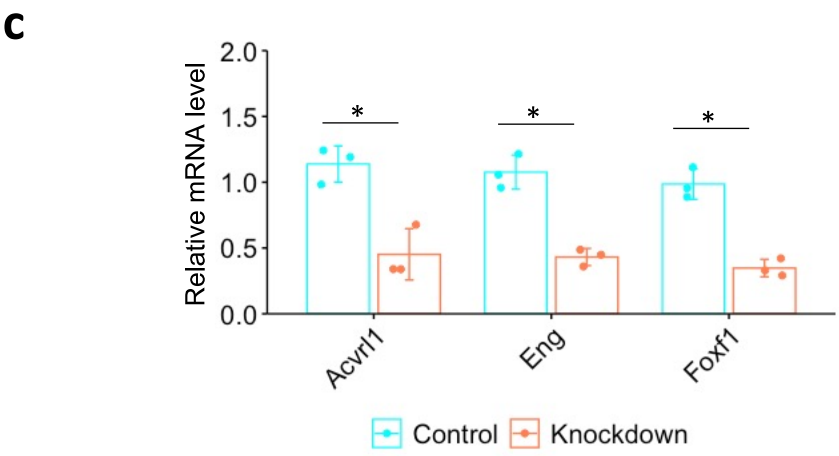
c
Relative mRNA level
2.0
1.5
1.0
0.5
0.0
*
*
*
Acvrl1
Eng
Foxf1
Control
Knockdown

Supplemental Figure 16. BMP9 treatment changes neither pSMAD2 nor total SMAD2 in MFLM-91U cells *in vitro*. *a,* qRT-PCR shows that *Foxf1* mRNA is decreased in fetal lung MFLM-91U cells after transfection with *Foxf1*-specific siRNA (siFoxf1). Scrambled siRNA (siControl) was used as a control. Expression levels were normalized to *β-Actin* mRNA. Data were shown as mean ± SD, and nonparametric Mann Whitney U test were performed were performed, and p value is 0.00047. (n=3 biological replicates) *b,* Western blot shows that BMP9 treatment does not change pSMAD2 and total SMAD2 in MFLM-91U cells. Inhibition of *Foxf1* by siRNA has no effect on pSMAD2 and total SMAD2 in BMP9-treated cells (n=3). Data were presented as mean ± SD, and one-way ANOVA followed by Tukey's test (two-tailed) were performed. *c,* qRT-PCR shows decreased levels of *Acvrl1, Eng* and *Foxf1* mRNAs after siRNA transfection in MFLM-91U cells (n=3). Cells were transfected with siRNAs specific to *Acvrl1, Eng* or *Foxf1*. Scrambled siRNA was used as a control. Expression levels were normalized to *β-Actin* mRNA. Data were shown as mean ± SD, and nonparametric Mann Whitney U test were performed, and p values are 0.075509 (Acvrl1), 0.01534 (Eng), 0.0118 (Foxf1). $p<0.05$ is *, $p<0.01$ is **, ns is not significant.

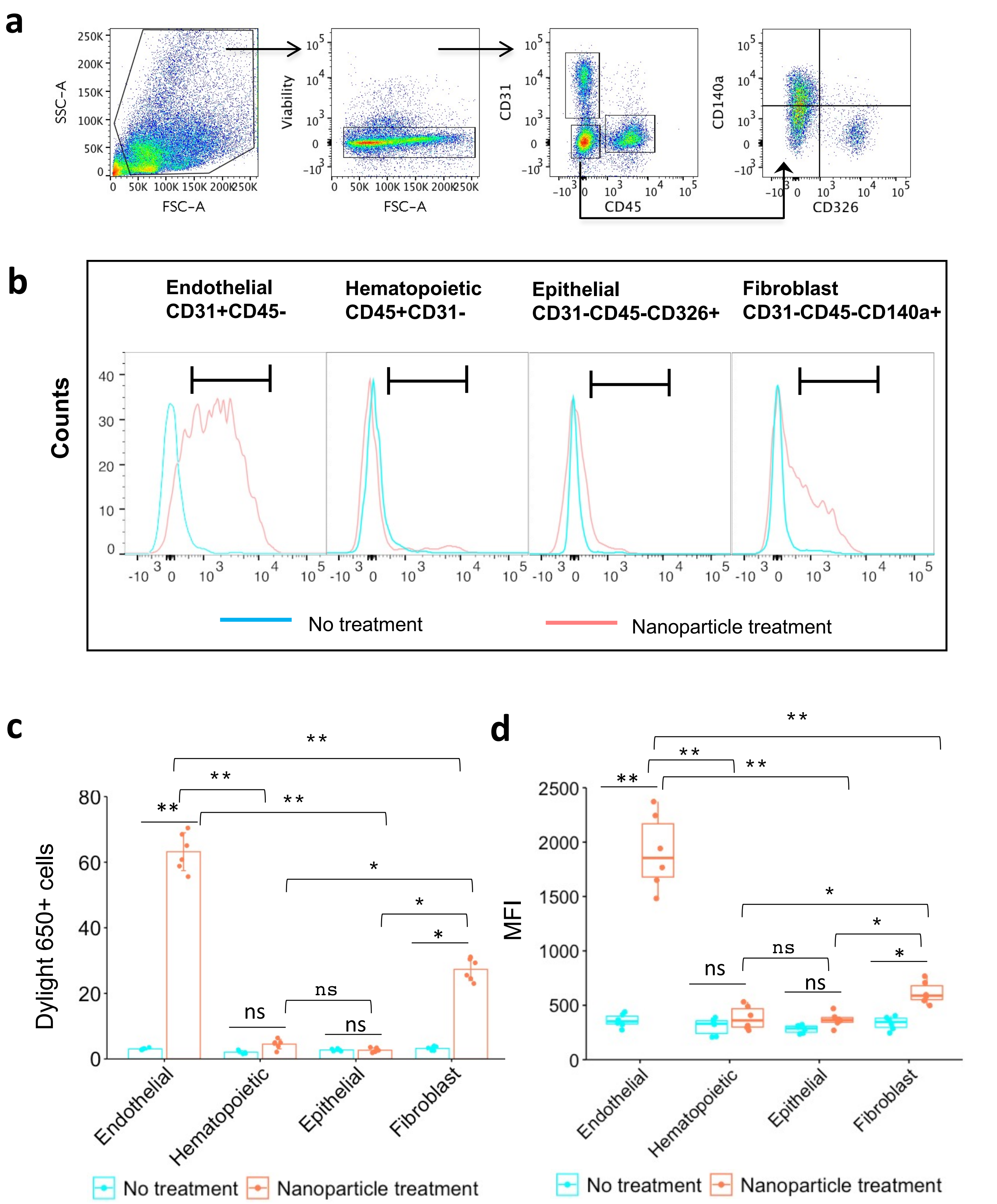
a
SSC-A
FSC-A
Viability
CD31
CD45
CD140a
CD326
b
Endothelial
CD31+CD45-
Hematopoietic
CD45+CD31-
Epithelial
CD31-CD45-CD326+
Fibroblast
CD31-CD45-CD140a+
Counts
No treatment
Nanoparticle treatment
c
Dylight 650+ cells
Endothelial
Hematopoietic
Epithelial
Fibroblast
No treatment
Nanoparticle treatment
d
MFI
Endothelial
Hematopoietic
Epithelial
Fibroblast
No treatment
Nanoparticle treatment

Supplemental Figure 17. PEI_{600}-MA_5/PEG-OA/Cho nanoparticles target endothelial cells and fibroblasts in the neonatal mouse lung. *a,* FACS-gating strategy shows expression of CD31, CD45, CD326 and CD140a in mouse P4 lungs. Pulmonary endothelial cells were identified as $CD31^+CD45^-$ cells, whereas hematopoietic cells were identified as $CD45^+CD31^-$ cells. Epithelial cells ($CD326^+CD31^-CD45^-$) and fibroblasts ($CD140a^+CD31^-CD45^-$) were detected within $CD31^-$ $CD45^-$ cell subset. Dylight 650- labeled PEI_{600}-MA_5/PEG-OA/Cho nanoparticles were injected at P2. Lungs were harvested at P4. *b,* Histograms show the presence of nanoparticles in endothelial cells and fibroblasts (red lines). Untreated mice were used as controls (blue lines). *c-d,* Bar graphs show the percentage of cells containing the nanoparticles in each respiratory cell type (*c* Data were presented as mean ± SD) and the Mean Fluorescence Intensity (MFI) for Dylight 650 (*d* Boxplots: center line, median; box boundary, first and third quartiles; whiskers denote 5th–95th percentile). MFI of Dylight 650 indicates the amounts of nanoparticles that are present in endothelial, hematopoietic, epithelial cells and fibroblasts (n=6 mice per group). The largest percentage of Dylight 650-positive cells and the largest nanoparticle load are detected in endothelial cells. One-way ANOVA followed by Tukey's test (two-tailed) were performed, $p<0.05$ is *, $p<0.01$ is **, ns is not significant.

a

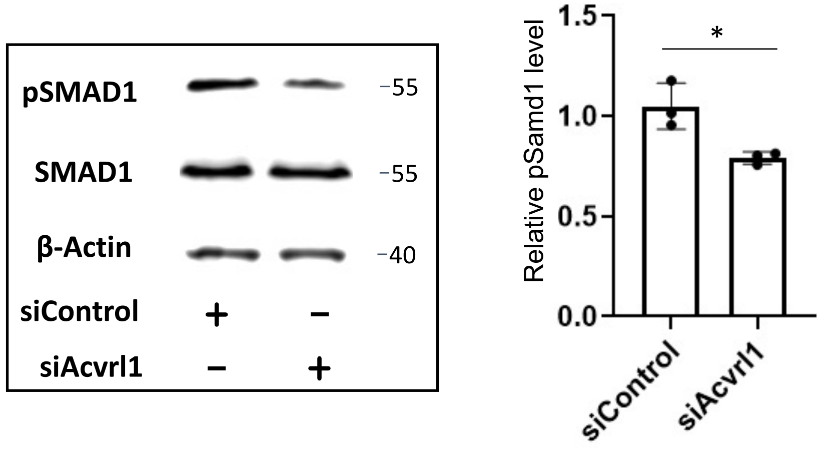
pSMAD1
-55
SMAD1
-55
β-Actin
-40
siControl + −
siAcvrl1 − +
Relative pSamd1 level
1.5
1.0
0.5
0.0
*
siControl
siAcvrl1

b

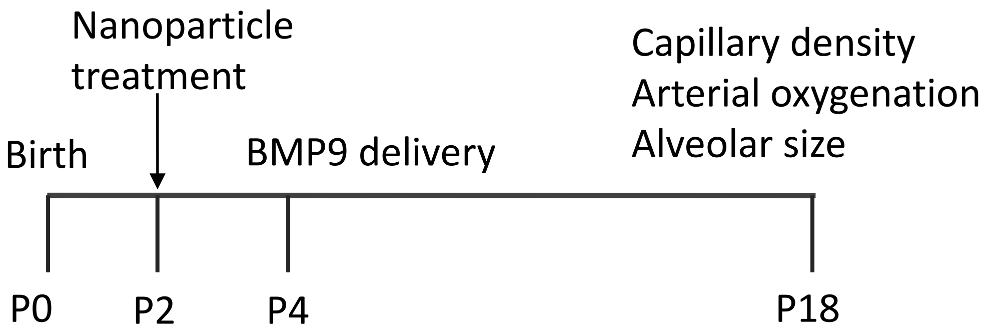
Nanoparticle treatment
Birth
BMP9 delivery
Capillary density
Arterial oxygenation
Alveolar size
P0
P2
P4
P18

c

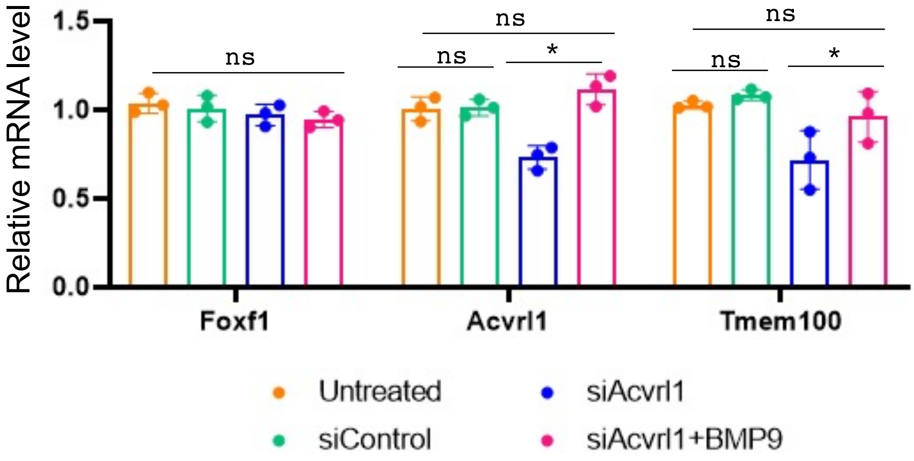
Relative mRNA level
1.5
1.0
0.5
0.0
ns
*
Foxf1
Acvrl1
Tmem100
Untreated
siControl
siAcvrl1
siAcvrl1+BMP9

Supplemental Figure 18. BMP9 treatment increases *Acvrl1* and *Tmem100* mRNAs in purified pulmonary endothelial cells from *siAcvrl1*-treated mice. *a,* Western blow shows that nanoparticle-mediated delivery of *Acvrl1* siRNA (siAcvrl1) decreases pSMAD1 in mouse lungs (n=3). Nanoparticles with scrambled siRNA were used as a control (siControl). Nanoparticles were delivered i.v. to P2 mice. Lungs were harvested at P4. Data were presented as mean ± SD, and T-test (two-tailed) analyses were performed with GraphPad Prism, P value = 0.01979. *b,* Schematic shows treatments of *WT* and *Foxf1*$^{WT/S52F}$ neonatal mice with siRNA-containing nanoparticles at P2 and recombinant BMP9 at P4. Mice were examined at P18. *c,* qRT-PCR shows that BMP9 treatment increases *Acvrl1* and *Tmem100* mRNAs in purified pulmonary endothelial cells from *siAcvrl1*-treated mice (n=3). Data were presented as mean ± SD, and one-way ANOVA followed by Tukey's test (two-tailed) were performed. P values are 0.01867 (Acvrl1), and 0.02043 (Tmem100). $p<0.05$ is *, ns is not significant. Endothelial cells were purified from the lung tissue using immunomagnetic beads as $CD31^{+}CD45^{-}$ cells. BPM9 treatment did not change *Foxf1* mRNA in lung endothelial cells.

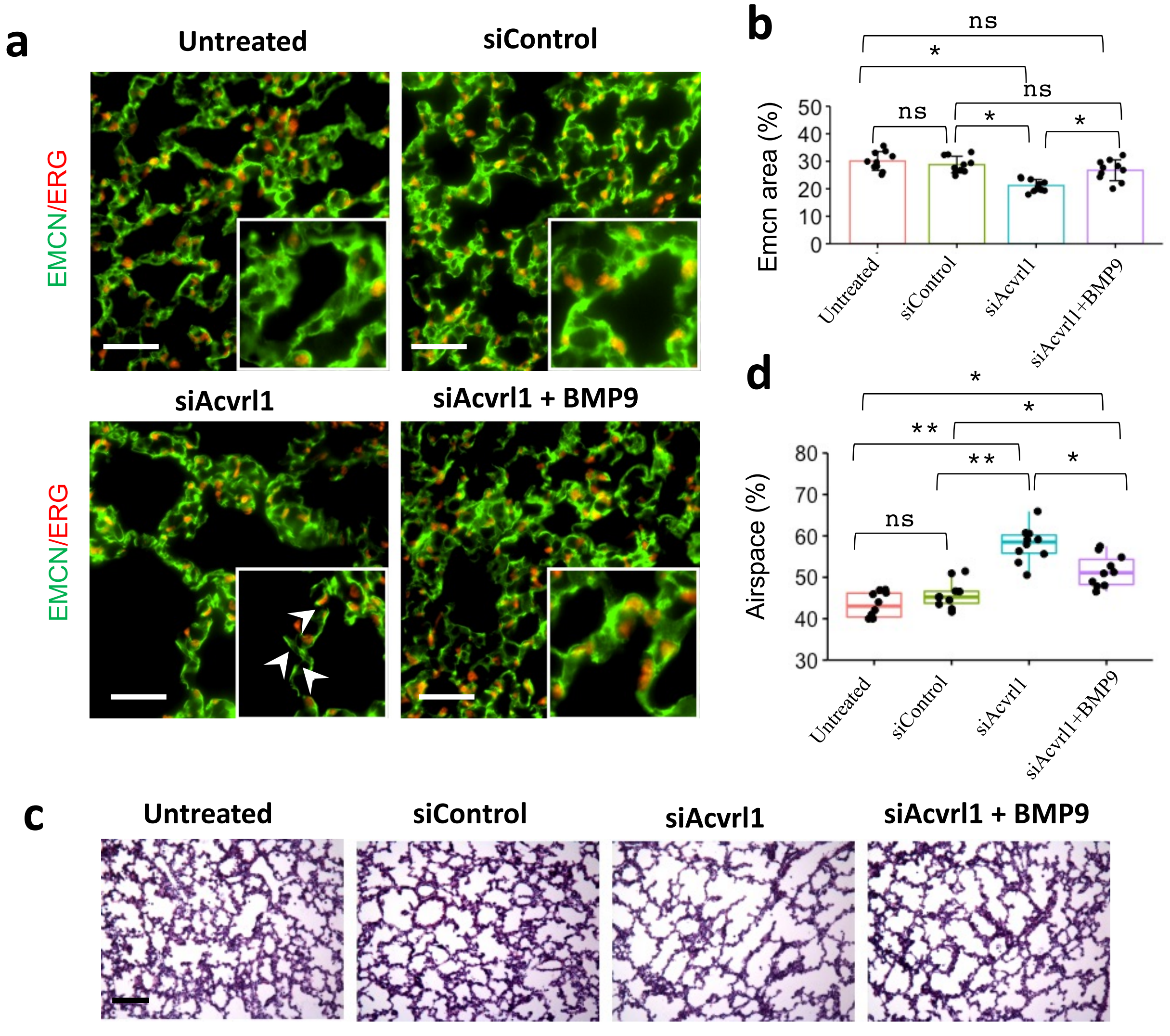
a
Untreated
siControl
siAcvrl1
siAcvrl1 + BMP9
EMCN/ERG
EMCN/ERG
b
Emcn area (%)
Untreated
siControl
siAcvrl1
siAcvrl1+BMP9
ns
*
ns
*
*
ns
d
Airspace (%)
Untreated
siControl
siAcvrl1
siAcvrl1+BMP9
**
**
*
*
*
ns
c
Untreated
siControl
siAcvrl1
siAcvrl1 + BMP9

Supplemental Figure 19. BMP9 treatment increases alveolar capillary density and improves alveologenesis in *siAcvrl1*-treated mice. *a-b,* Alveolar capillary density is decreased after nanoparticle delivery of *Acvrl1* siRNA. Data were presented as mean ± SD. Nanoparticles were delivered at P2. Immunostaining for endothelial markers endomucin (EMCN) and ERG was performed using P18 mouse lungs (n=10). Alveolar capillary density is restored after administration of BMP9. BMP9 treatment was performed at P4. Scale bars are 20μm. *c-d,* Nanoparticle delivery of *Acvrl1* siRNA causes alveolar simplification as shown by increased sizes of alveoli. BMP9 treatment decreases alveolar simplification in *siAcvrl1*-treated mice. Alveolar sizes were measured using H&E-stained lung sections from P18 mice (n=10). Boxplots: center line, median; box boundary, first and third quartiles; whiskers denote 5th–95th percentile. Scale bars are 50μm. (b-d) One-way ANOVA followed by Tukey's multiple comparison test (two-tailed) were performed. $p<0.01$ is **, $p<0.05$ is *, ns is not significant.

Wang et al, Supplemental Fig. 20

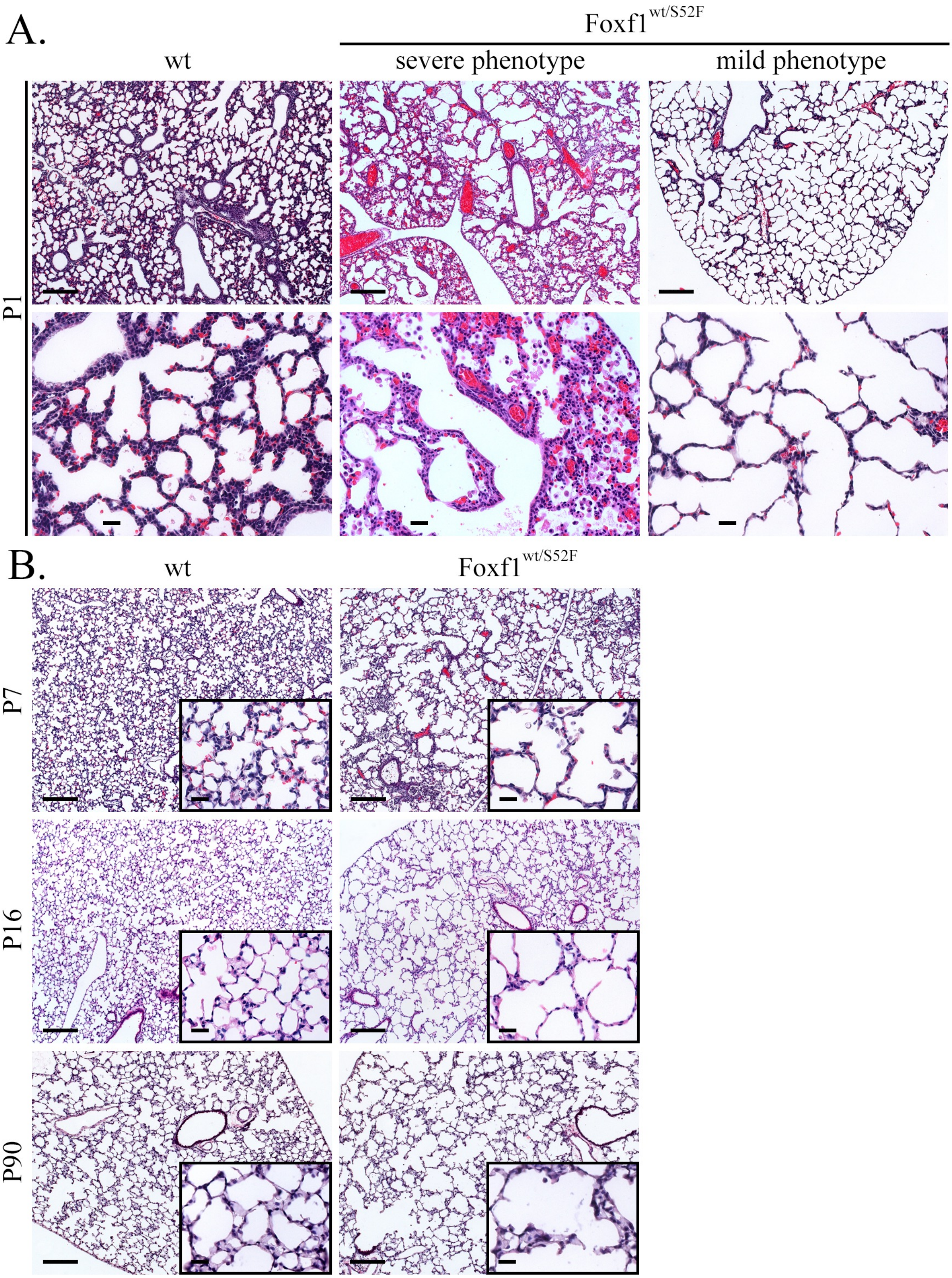

Supplemental Figure 20. H&E staining shows severe and mild phenotypes in *Foxf1*$^{WT/S52F}$ mutant mice. *a,* Newborn *Foxf1*$^{WT/S52F}$ mutant mice with the most severe phenotype exhibit lung inflammation, hemorrhage, and alveolar simplification which cause early neonatal lethality. Newborn *Foxf1*$^{WT/S52F}$ mutant mice with the mild phenotype exhibit alveolar simplification without hemorrhage and inflammation. *b,* H&E staining shows lung histology in wild type (wt) and surviving *Foxf1*$^{WT/S52F}$ littermates at P7, P16 and P90. The experiments were repeated twice with similar results. Scale bars are 200μm for low magnification, and 25μm for high magnification.

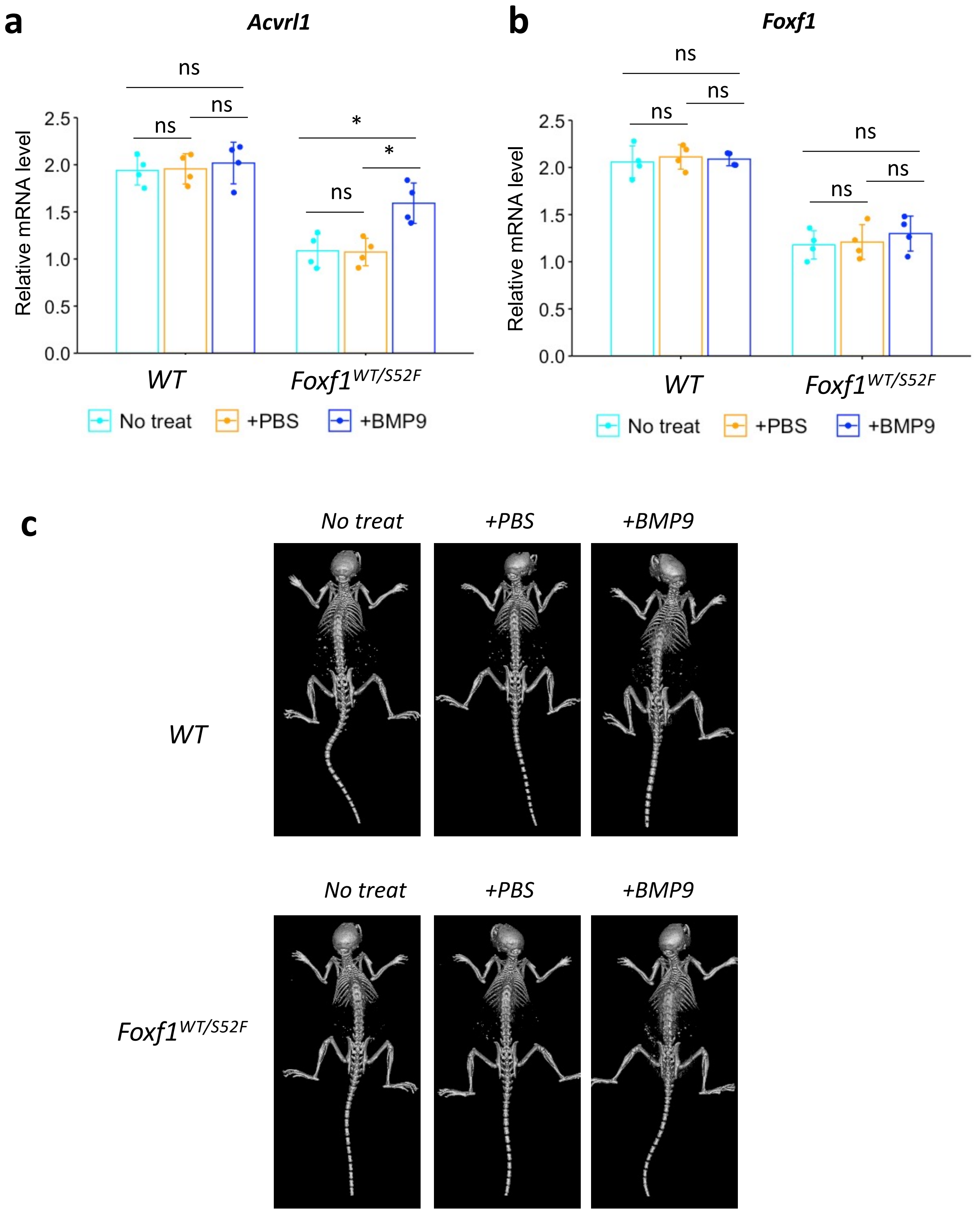
a
Acvrl1
Relative mRNA level
2.5
2.0
1.5
1.0
0.5
0.0
ns
ns
ns
*
*
ns
WT
Foxf1 WT/S52F
No treat
+PBS
+BMP9
b
Foxf1
Relative mRNA level
ns
ns
ns
ns
ns
ns
WT
Foxf1 WT/S52F
No treat
+PBS
+BMP9
c
No treat
+PBS
+BMP9
WT
No treat
+PBS
+BMP9
Foxf1 WT/S52F

Supplemental Figure 21. BMP9 treatment increases *Acvrl1* expression in *Foxf1*$^{WT/S52F}$ lungs. *a,* qRT-PCR shows that BMP9 administration increases *Acvrl1* mRNA in lung tissue of *Foxf1*$^{WT/S52F}$ mutant mice but not in *WT* mice. Nanoparticles were delivered at P3, and lungs were harvested at P18. (n=4 mice) *b*, *Foxf1* mRNA was unaltered after BMP9 treatment of *Foxf1*$^{WT/S52F}$ and *WT* mice. (n=4 mice) (a-b) Data were shown as mean ± SD. One-way ANOVA followed by Tukey's test (two-tailed) were performed, and p values are 0.01456 (BMP9 vs No treat), 0.01869 (BMP9 vs PBS). $p<0.05$ is *, $p<0.01$ is **, ns is not significant. *c,* microCT images show that BMP9 treatment does not cause abnormal ossification or ectopic bone formation in *Foxf1*$^{WT/S52F}$ or *WT* littermates.

Wang et al, Supplemental Fig. 22

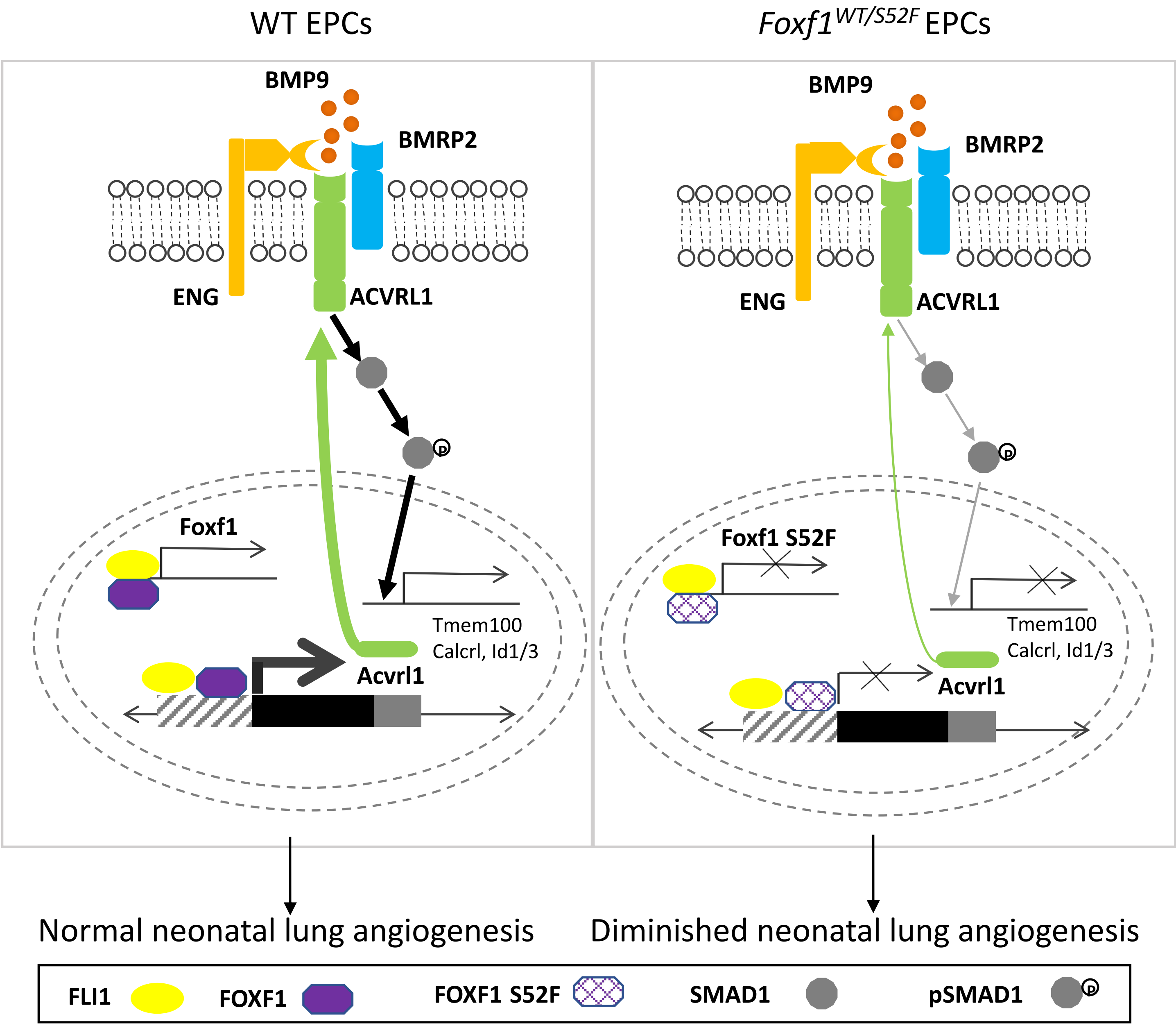

Supplemental Figure 22. Model of FOXF1-mediated regulation of BMP9/ACVRL1/SMAD1 signaling in FOXF1$^+$ EPCs. Diagrams show regulation of the BMP9/ACVRL1 signaling pathway in WT (left panel) and FOXF1 mutant (*Foxf1*$^{WT/S52F}$) EPCs (right panel). FOXF1 synergizes with ETS transcription factor FLI1 to stimulate transcription of *Acvrl1* and *Foxf1* genes in FOXF1$^+$ EPCs. Together with BMPR2 and ENG, ACVRL1 protein forms a receptor complex for BMP9, which activates gene expression through phosphorylation of SMAD1 (pSMAD1). *Foxf1*$^{WT/S52F}$ EPCs exhibit decreased ACVRL1 mRNA and protein, causing decreased cell surface expression of ACVRL1 and reduced BMP9/ACVRL1/pSMAD1 signaling which leads to diminished neonatal lung angiogenesis.

Uncropped blots for Fig7b

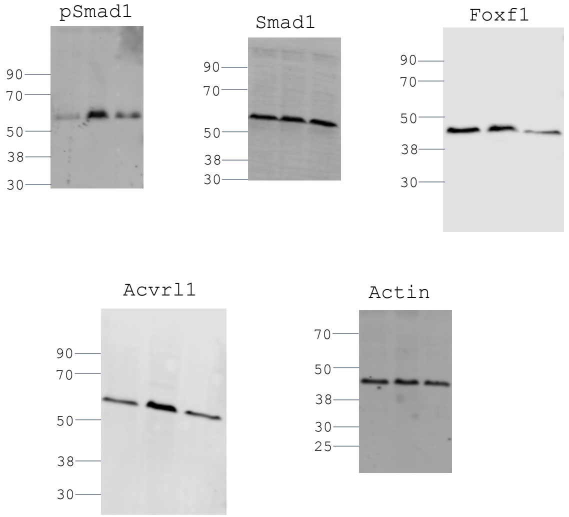

Uncropped blots for
Suppl. Fig16b

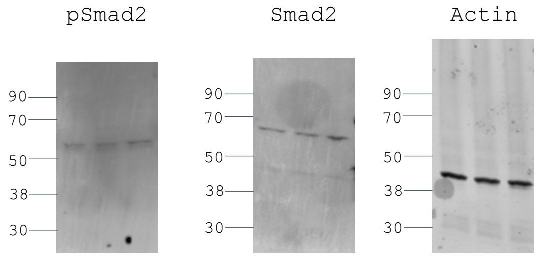

Uncropped blots for
Suppl. Fig18a

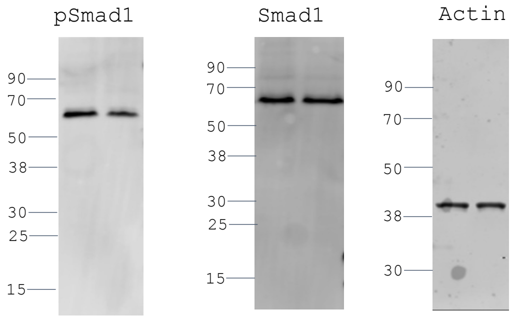

Supplemental Figure 23. The uncropped western blots.
